# Uncovering Latent Structures: A Bayesian Approach to Estimating Q-Matrix and Attribute Hierarchies in Cognitive Diagnostic Models

**DOI:** 10.1017/psy.2026.10093

**Published:** 2026-02-20

**Authors:** Xue Wang, Yinghan Chen, Shiyu Wang

**Affiliations:** 1 Key Laboratory of Applied Statistics of MOE, Key Laboratory of Big Data Analysis of Jilin Province, School of Mathematics and Statistics, Northeast Normal University, Changchun, Jilin, China; 2 Department of Mathematics and Statistics, University of Nevada Reno, USA; 3 Department of Educational Psychology, University of Georgia, Athens, GA, USA

**Keywords:** Bayesian method, cognitive diagnosis models, hierarchical structure, Q-matrix

## Abstract

Cognitive diagnostic models (CDMs) provide fine-grained diagnostic feedback by modeling the relationship between latent attributes and item responses. Two key components required for CDM implementation are the Q-matrix, which links items to attributes, and the attribute hierarchy, which defines prerequisite relationships among attributes. In many practical settings, both structures are specified by experts based on cognitive theory. In this article, we propose a novel Bayesian estimation method that simultaneously learns the Q-matrix, the attribute hierarchy, and the parameters of the deterministic inputs, noisy “and” gate (DINA) model. We develop a Metropolis–Hastings within Gibbs algorithm and integrate a mini-batch strategy to improve computational efficiency. We conducted a series of simulation studies to evaluate the performance of the proposed algorithm under varying conditions, including sample size, test length, hierarchy structure, mini-batch size, and threshold settings. The results demonstrate strong recovery rates for both latent structures and item parameters, confirming the accuracy and robustness of our method. A real data application further illustrates the utility of the proposed framework in uncovering interpretable diagnostic structures. Our findings offer practical guidance for researchers seeking to implement CDMs when both the Q-matrix and attribute hierarchy are unknown.

## 1 Introduction

Cognitive diagnostic models (CDMs) are a family of restricted latent class models that describe how a person’s underlying discrete latent traits, in conjunction with item characteristics, influence their responses to test items. CDMs provide fine-grained diagnostic information about whether an individual has mastered specific skills or attributes. This detailed feedback makes CDMs particularly valuable in a wide range of applications across education, behavioral sciences, and social sciences (Chen et al., 2015; De La Torre & Douglas, 2004, 2008; Tatsuoka, 1984; Von Davier, 2008).

As a class of confirmatory psychometric models, CDMs require several key components to be specified prior to application to a dataset. Two essential latent structures must be defined. The first is the Q-matrix (Tatsuoka, 1983), which specifies the relationship between items and the attributes they measure. The second structure concerns the relationships among the latent attributes themselves. Although many earlier studies did not impose any assumptions about hierarchical structures among attributes, accumulating evidence suggests that hierarchical dependencies are frequently present in practice. For example, in mathematics, multiplication is essentially a simplified form of addition, such as 2×4=2+2+2+2=8 . Therefore, mastering addition is widely regarded as a fundamental prerequisite for mastering multiplication. Recognizing such dependencies, scholars have explored ways to incorporate attribute hierarchies into CDMs. As a result, a growing body of methodological research has aimed to formally integrate hierarchical structures—where mastery of one attribute is required before another—into CDMs (e.g., Leighton et al., 2004; Ma & Xu, 2022; Templin & Bradshaw, 2014).

In many CDM-related studies, the Q-matrix and attribute hierarchy are often fully or partially specified by test developers based on cognitive theory or expert judgment. More recently, data-driven approaches have emerged that estimate the Q-matrix, attribute hierarchy, or both directly from examinees’ response data. The literature on Q-matrix estimation can be broadly divided into two categories: validation and refinement of Q-matrix with partial knowledge (Chiu, 2013; DeCarlo, 2012; de la Torre & Chiu, 2016; Templin & Henson, 2006; Wang et al., 2020), and completely data-driven estimation of unknown Q-matrix (Chen et al., 2015; Chen et al., 2018; Chung, 2019; Liu et al., 2012, 2013; Xu & Shang, 2018). The estimation methods include both frequentist (Chen et al., 2015; Liu et al., 2012, 2013; Xiang, 2013) and Bayesian approaches (Chen et al., 2018; Chung, 2019; Culpepper, 2019; Templin & Henson, 2006).

For the estimation of attribute hierarchy structures, Wang and Lu (2021) introduced two exploratory methods using regularization techniques to learn hierarchies directly from data. Liu et al. (2022) proposed a z-statistic approach to assess the significance of structural parameter estimates, and Zhang et al. (2024) refined this method for improved accuracy. Moreover, Lee and Gu (2024) proposed the latent conjunctive Bayesian networks (LCBNs) to unify the attribute hierarchy and the Bayesian network, and developed a two-step EM algorithm to estimate the hierarchy structure and item parameters. Assuming a known Q-matrix, Chen and Wang (2023) developed a Bayesian framework for estimating attribute hierarchies using a Metropolis-within-Gibbs sampler.

A few studies have sought to estimate both the Q-matrix and the attribute hierarchy jointly. For example, Ma et al. (2023) proposed a penalized likelihood approach to first select significant latent classes and item parameter structures, then recover the number of attributes, the attribute hierarchy, the Q-matrix, and item-level models. These developments reflect a growing interest in flexible, data-driven frameworks for cognitive diagnosis modeling.

The goal of this study is to address the problem of simultaneously learning the Q-matrix, attribute hierarchy, and CDM parameters from examinees’ item response data. Building on the deterministic inputs, noisy “and” gate (DINA) model, we propose a Bayesian estimation method that jointly estimates the attribute hierarchy, Q-matrix, item parameters, and examinees’ latent attribute profiles—without assuming prior knowledge of either the Q-matrix or the attribute hierarchy. Our approach extends the work of Chen and Wang (2023) on attribute hierarchy learning and Chung (2019) on Q-matrix estimation by developing a Metropolis–Hastings within Gibbs (MH-Gibbs) algorithm to estimate all parameters in a unified framework. To improve computational efficiency, we further incorporate a mini-batch strategy, enabling faster estimation while maintaining accuracy. Moreover, compared with Ma et al. (2023), which adopts a two-step procedure to estimate the attribute hierarchy and the Q-matrix, our method allows for the simultaneous estimation of all parameters in a unified framework. Moreover, unlike deterministic methods, our Bayesian approach summarizes the estimation of Q and G based on posterior samples. This enables a more flexible, theory-driven validation process, where experts can evaluate the plausibility of multiple high-probability structures informed by domain knowledge, rather than relying solely on a single point estimate.

In the remainder of this article, we first present the problem setup in Section 2. Section 3 introduces our proposed Bayesian estimation framework and details the development of an MH-Gibbs algorithm for jointly estimating the attribute hierarchy, Q-matrix, item parameters, and examinees’ latent attribute profiles under the DINA model. In Section 4, we provide further discussions on several issues of implementing the algorithm to obtain final point estimation of model parameters, including the selection of mini-batch sample size, the potential label-switching issues, and the determination of cut-off values for finalizing the latent structures. In Section 5, we conduct a comprehensive simulation study to evaluate the performance of the proposed algorithm and investigate the impact of various data conditions. Section 6 applies the proposed method to a real dataset to further demonstrate its practical utility. Finally, Section 7 concludes the article with a summary of key findings and outlines directions for future research.

## 2 Preliminaries: Model setup

### 2.1 DINA model

To introduce DINA model formulation, let i~(i=1,⋯,N) denote examinees, j~(j=1,⋯,J) denote items, and k~(k=1,⋯,K) denote attributes. Whether examinee *i* masters the attribute *k* is denoted as a binary variable $\alpha_{ik}$ , where $\alpha_{ik}=1$ if examinee *i* has mastered the attribute *k* and $\alpha_{ik}=0$ otherwise. We use a *K*-dimensional binary vector, $\alpha_{i}={\left(\alpha_{i1},\cdots ,\alpha_{ik},\cdots ,\alpha_{iK}\right)}^{\text{T}}$ , to represent the latent attribute profile of examinee *i*. Therefore, there are $C=2^{K}$ possible attribute profiles. Let class *c* denote the bijection index of the attribute profile, where c=0,⋯,C−1 .

Furthermore, a J×K -dimensional Q -matrix, $\text{Q}={\left(q_{1},\cdots ,q_{j},\cdots ,q_{J}\right)}^{\text{T}}$ , is introduced to describe which attributes are measured by each item. Here, $q_{j}^{\text{T}}=(q_{j1},\cdots ,q_{jk},\cdots ,q_{jK})$ is the *j*-th row of Q -matrix, corresponding to measured attributes by item *j* with $q_{ik}=1$ if the attribute *k* is required by item *j*, and $q_{ik}=0$ otherwise. Let $Y_{ij}$ denote the binary item response variable for the examinee *i* to the item *j*, where $Y_{ij}$ equals 1 if the *i*th examinee correctly answered the item *j*, and 0 otherwise.

The DINA model assumes an examinee need to master all the required attributes for item *j* to have a high chance to answer that item correctly. To reflect this assumption, the ideal response variable $\eta_{ij}=\prod_{k=1}^{K}\alpha_{ik}^{q_{jk}}$ is defined to formulate the item response model. Here, $\eta_{ij}=1$ indicates that examinee *i* possesses all the required attributes for item *j*; otherwise, $\eta_{ij}=0$ . In addition, two item parameters are introduced for each item *j*: the slipping parameter $s_{j}$ and the guessing parameter $g_{j}$ which can be formally defined by

(1) $$\begin{array}{cc} s_{j} & =P(Y_{ij}=0|\eta_{ij}=1),g_{j}=P(Y_{ij}=1|\eta_{ij}=0). \end{array}$$

Then, the probability of a correct response for the DINA can be expressed as

(2) $$\begin{array}{c} P(Y_{ij}=1|\alpha_{i}=\alpha_{c},g_{j},s_{j})=g_{j}^{1-\eta_{cj}}{\left(1-s_{j}\right)}^{\eta_{cj}}, \end{array}$$

where $\alpha_{c}$ denotes one possible attribute profile value and $\eta_{cj}$ is the corresponding ideal response variable.

### Attribute hierarchy and Q -matrix

2.2

**Figure 1:**
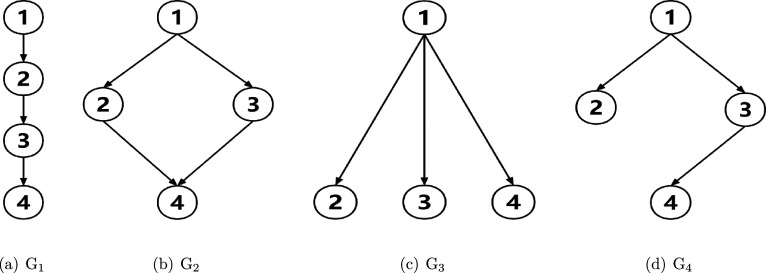
Four attribute hierarchy structures, arranged from left to right: (a) $\backslash textG_{1}$ Linear; (b) $\backslash textG_{2}$ Convergent; (c) $\backslash textG_{3}$ Divergent; and (d) $\backslash textG_{4}$ Unstructured.

One type of latent structure with CDM is the attribute hierarchy, which may be commonly considered when defining attributes within many educational and psychological tests. Based on the nature of assessment, attributes may have different types of dependency, where one attribute may be a prerequisite for another attribute. Four types of popular hierarchical structure are discussed by Leighton et al. (2004), which are presented as four directed acyclic graphs (DAGs) in Figure 1. For a DAG, each node represents an attribute, and a directed edge between two nodes indicates a prerequisite relationship, where the attribute represented by the source node is required for mastering the attribute represented by the target node. For instance, with the linear hierarchy represented by Figure 1(a), the directed edge 1→2 indicates that the mastery of $\alpha_{1}$ is a prerequisite for mastering $\alpha_{2}$ .

In this article, we adopt the same notation for describing the attribute hierarchy as Chen and Wang (2023). Let V and *E* denote the set of attributes and the directed edges, respectively, and G(V;E) denote the attribute hierarchy structure. Mathematically, one G(V;E) can be presented by its corresponding adjacency matrix, G . For example, let G(V;E) represent the linear hierarchy in Figure 1(a). With this structure, V={1,2,3,4} and E={1→2,2→3,3→4} . This particular G(V;E) can be denoted by its adjacency matrix G as

$$\begin{array}{c} \text{G}=\left[\begin{array}{ccccccccccccc} 0 & 1 & 0 & 00 & 0 & 1 & 00 & 0 & 0 & 10 & 0 & 0 & 0 \end{array}\right], \end{array}$$

where $\text{G}(k,k^{'})=1$ represents there is a directed edge k→k' and 0 otherwise. When there is no hierarchical structure between any attributes, all elements in G are 0, and we denote this structure as $\backslash textG_{null}$ . Because of this one-to-one correspondence between a DAG and its adjacency matrix G , we will just use the adjacency matrix G to denote its corresponding hierarchical structure G(V,E) .

In addition to the direct relationships discussed above, there are also indirect dependencies among attributes. To better capture both direct and indirect dependencies between attributes, the reachability matrix R is introduced, where $\text{R}(k,k^{'})=1$ represents that there exists a path from *k* to k' , and it can be obtained through Boolean addition and multiplication of the adjacency matrix (Tatsuoka, 2012). The reachability matrix R of Figure 1(a) can be expressed as

$$\begin{array}{c} \text{R}=\left[\begin{array}{ccccccccccccc} 1 & 1 & 1 & 10 & 1 & 1 & 10 & 0 & 1 & 10 & 0 & 0 & 1 \end{array}\right]. \end{array}$$

Note that different hierarchical structures may lead to the same reachability matrix. For example, consider the structure $\backslash textG^{*}$ , corresponding to $V^{*}=\{1,2,3,4\}$ and $E^{*}=\{1\to 2,2\to 3,3\to 4,1\to 4\}$ . The reachability matrix of $\backslash textG^{*}$ will be the same as that of G for Figure 1(a), meaning that both exhibit the same dependencies between attributes. However, it is clear that the structure of G in Figure 1(a) is simpler. Therefore, our proposed Bayesian estimation method estimates the transitive reduction of G , which refers to a subgraph of G with the fewest edges that maintains the same reachability as G (Chen & Wang, 2023).

#### The impact of G to latent attribute profile distribution

2.2.1

Typically when assuming no attribute hierarchy among *K* attributes, that is, under $\backslash textG_{null}$ , there are $C=2^{K}$ possible attribute profiles. With a specific attribute hierarchical structure, the number of possible attribute profiles is reduced from $2^{K}$ . For example, consider the linear structure $\backslash textG_{1}$ in Figure 1(a), the attribute profile (1,0,1,0) violates the relationship that $\alpha_{2}$ is the prerequisite of $\alpha_{3}$ , thus cannot exist with $\backslash textG_{1}$ . Therefore, the attribute profile space depends on the specific hierarchical structure G . Denote $C_{G}$ the number of permissible attribute profiles under G . Here, we provide a definition of an equivalence class. Let $\alpha_{c}\backslash stackrel\text{G}=\alpha_{\tilde{c}}$ indicate that $\alpha_{c}$ and $\alpha_{\tilde{c}}$ are equivalent under structure G . Specifically, if a mastered attribute (i.e., an attribute with a value of 1) has a prerequisite that has not been mastered, it will be reassigned to 0. For example, as mentioned above, the attribute profile (1,0,1,0) is not permissible under linear structure $\backslash textG_{1}$ and is thus reassigned to (1,0,0,0), that is, $\left(1,0,1,0)\backslash stackrel\backslash textG_{1}=(1,0,0,0\right)$ .

#### Q -matrix under G

2.2.2

Another important latent structure under CDM is the Q -matrix. When attribute hierarchy exists, we assume that the hierarchical structure also affects the space of the Q -matrix. In other words, the q -vector needs to reflect the underlying attribute hierarchy as well. For example, considering the linear structure $\backslash textG_{1}$ , we need to make the notations consistent in Figure 1(a), q=(0,1,0,0) equivalent to q=(1,1,0,0) , indicating that an item measuring $\alpha_{2}$ also needs to measure $\alpha_{1}$ under this linear structure. Therefore, we introduce reduced Q -matrix $\backslash textQ_{r}$ (Tatsuoka, 2012), which including all possible q -vectors under the hierarchical structure. In this case, the total $2^{K}-1$ possible q -vectors under $\backslash textG_{null}$ are reduced to four possible q -vectors under the linear structure $\backslash textG_{1}$ . In this study, a structured Q -matrix constructed from the possible q -vectors in $\backslash textQ_{r}(\text{G})$ is used to reflect the attribute hierarchical structure. The above relationship is shown by Equation (3), where each column in a Q -matrix represents a possible q -vector:

(3) $$\begin{array}{c} \backslash textQ_{all}^{\text{T}}=\left[\begin{array}{cc} 1\\ 0\\ 0\\ 0\\ 1\\ 1\\ 1\\ 0\\ 0\\ 0\\ 1\\ 1\\ 1\\ 0\\ 1 & 0\\ 1\\ 0\\ 0\\ 1\\ 0\\ 0\\ 1\\ 1\\ 0\\ 1\\ 1\\ 0\\ 1\\ 1 & 0\\ 0\\ 1\\ 0\\ 0\\ 1\\ 0\\ 1\\ 0\\ 1\\ 1\\ 0\\ 1\\ 1\\ 1 & 0\\ 0\\ 0\\ 1\\ 0\\ 0\\ 1\\ 0\\ 1\\ 1\\ 0\\ 1\\ 1\\ 1\\ 1 \end{array}\right]\backslash stackrelreduced⟶\left(\begin{array}{cc} 1\\ 1\\ 1\\ 1 & 0\\ 1\\ 1\\ 1 & 0\\ 0\\ 1\\ 1 & 0\\ 0\\ 0\\ 1 \end{array}\right)\backslash stackrel△=\backslash textQ_{r}{\left(\backslash textG_{1}\right)}^{\text{T}}. \end{array}$$

## 3 Bayesian estimation

### 3.1 Bayesian formulation

To start with, denote item response matrix for *N* examinees across *J* items as $\backslash textbfY={\left(Y_{1},\cdots ,Y_{i},\cdots ,Y_{N}\right)}^{\text{T}}$ , where $Y_{i}={\left(y_{i1},\cdots ,y_{ij},\cdots ,y_{iJ}\right)}^{\text{T}}$ , i=1,⋯,N , is the response vector of examinee *i* to *J* items. In addition, let $\backslash textbfA={\left(\alpha_{1},\cdots ,\alpha_{i},\cdots ,\alpha_{N}\right)}^{\text{T}}$ be the N×K matrix of attribute profiles of all examinees. Denote the parameter $\pi =(\pi_{1},\cdots ,\pi_{c},\cdots ,\pi_{C})$ as the latent class probability vector, that is, $\pi_{c}=P(\alpha_{i}=\alpha_{c})$ , satisfying $\sum \pi_{c}=1$ . Let $s={\left(s_{1},\cdots ,s_{J}\right)}^{T}$ and $g={\left(g_{1},\cdots ,g_{J}\right)}^{T}$ denote the slipping and guessing parameters for all items.

Given the conditional independent assumption, the joint distribution of Y given parameters s,g,π,A,Q , and G can be written as

(4) $$\begin{array}{c} p(Y|s,g,\pi ,A,\text{Q},\text{G})=\prod_{i=1}^{N}\prod_{j=1}^{J}\sum_{c=1}^{C}\pi_{c}\sim p(Y_{ij}|\alpha_{i}=\alpha_{c},g_{j},s_{j},q_{j},\text{G}), \end{array}$$

where

(5) p(_Yij|_αi=_αc,_gj,_sj,_qj,\textG)=^(gj1−ηcj(1−sj)ηcj)yij^((1−gj)1−ηcjsjηcj)1−yij,_ηcj=_^∏k=1K_^αckqjk.

To estimate the DINA model item parameters s and g , the examinee parameters A and π , and the two latent structures G and Q , we first outline the joint posterior distribution of all model parameters.

Let $p(s_{j},g_{j})$ , $p(\alpha_{i}|\pi ,\text{G})$ , p(π∣G) , ~p(Q∣G) , and p(G) be the corresponding prior of $\left(s_{j},g_{j}\right)$ , $\alpha_{i}$ , π , Q , and G , respectively. The joint posterior distribution of all model parameters is

(6) $$\begin{array}{c} \begin{array}{ccc} \backslash , & p(s,g,\pi ,A,\text{Q},\text{G}|Y)\\ \backslash , & \;\propto \sim p(Y|s,g,\pi ,A,\text{Q},\text{G})p(s,g)\sim p(A|\pi ,\text{G})\sim p(\pi |\text{G})\sim p(\text{Q}|\text{G})\sim p(\text{G}) & \;\propto \sim p(Y|s,g,\pi ,A,\text{Q},\text{G})\left(\prod_{j=1}^{J}p(s_{j},g_{j})\right)\left(\prod_{i=1}^{N}p(\alpha_{i}|\pi ,\text{G})\right)\sim p(\pi |\text{G})\sim p(\text{Q}|\text{G})\sim p(\text{G}), \end{array} \end{array}$$

where p(Y∣s,g,π,A,Q,G) is the joint distribution function (4). In the following section, we specify the prior distributions of each parameter, followed by the inference of their posterior distributions.

### 3.2 Posterior inference

Firstly, the prior of $s_{j}$ and $g_{j}$ follows the Beta distribution with parameter $a_{s0}$ , $b_{s0}$ , $a_{g0}$ , and $b_{g0}$ :

(7) $$\begin{array}{c} p(s_{j},g_{j})\propto s_{j}^{a_{s0}-1}{\left(1-s_{j}\right)}^{b_{s0}-1}g_{j}^{a_{g0}-1}{\left(1-g_{j}\right)}^{b_{g0}-1}I(0<s_{j}+g_{j}<1). \end{array}$$

Then we can derive that posterior distribution of $s_{j}$ is a Beta distribution with parameter $a_{sj}$ and $b_{sj}$ as following:

(8) $$\begin{array}{c} s_{j}|\backslash textbfY,g,\pi ,A,\text{Q},\text{G}∼\backslash operatornameBeta(a_{sj},b_{sj})I(0<s_{j}<1-g_{j}), \end{array}$$

where

_asj=_^\sumi=1N_\etaij(1_−yij),\quad_bsj=_^\sumi=1N_\etaij_yij.

And the posterior distribution of $g_{j}$ is a Beta distribution with parameter $a_{gj}$ and $b_{gj}$ as following:

(9) $$\begin{array}{c} g_{j}|\backslash textbfY,s,\pi ,A,\text{Q},\text{G}∼\backslash operatornameBeta(a_{gj},b_{gj})I(0<g_{j}<1-s_{j}), \end{array}$$

where

_agj=_^\sumi=1N(1_−ηij)_yij,\quad_bgj=_^\sumi=1N(1_−ηij)(1_−yij).

Following Culpepper (2015), we assign Beta(1,1) priors to both $s_{j}$ and $g_{j}$ in this article, that is, $a_{s0}=b_{s0}=a_{g0}=b_{g0}=1$ .

Secondly, the prior of π under structure $\backslash textG_{null}$ is a Dirichlet distribution with parameter $\delta_{0}$ :

(10) $$\begin{array}{c} p(\pi |\delta_{0})\propto \prod_{c=0}^{C-1}\pi_{c}^{\delta_{0c}},0\leq \pi_{c}\leq 1,\sum \pi_{c}=1. \end{array}$$

However, as discussed in Section 2.2, the existence of a hierarchical structure among attributes imposes constraints on the attribute profiles. Specifically, some attribute profiles become impermissible under the hierarchy structure G . This restriction reduces the set of permissible attribute profiles, thereby influencing the distribution of π . Moreover, since G is allowed to change during the estimation process, the distributions of $\alpha_{i}$ also change accordingly. Therefore, when incorporating a hierarchical structure G , it is necessary to adjust the corresponding priors to reflect the structural constraints imposed by G .

Let $L_{G}$ denote the set of permissible attribute profiles under a specific hierarchical structure G and $C_{G}=|L_{G}|$ denote the number of permissible attribute profiles. Define $\pi_{G}$ as the current latent class probability vector. Thus, the prior of $\pi_{G}$ now follows an aggregated Dirichlet distribution with parameters $\delta_{0}^{G}$ :

(11) $$\begin{array}{c} p(\pi_{G}|\delta_{0}^{G})\propto \prod_{\alpha_{\tilde{c}}\in L_{G}}\pi_{\tilde{c}}^{\delta_{0\tilde{c}}^{G}}, \end{array}$$

where

$$\begin{array}{c} \pi_{\tilde{c}}=\sum_{\alpha_{c}\backslash stackrelG=\alpha_{\tilde{c}};c\in C}\pi_{c},\;\delta_{0\tilde{c}}^{G}=\sum_{\alpha_{c}\backslash stackrelG=\alpha_{\tilde{c}};c\in C}\delta_{c}. \end{array}$$

Based on the prior in Eq. (11) and the joint posterior distribution in Eq. (6), we can derive that posterior distribution of $\pi_{G}$ is a Dirichlet distribution with parameter $\delta_{G}$ as follows:

(12) $$\begin{array}{c} \pi_{G}|\backslash textbfY,s,g,\backslash textbfA,\text{Q},\text{G}∼\backslash operatornameDirichlet(\delta_{G}), \end{array}$$

where

$$\begin{array}{c} \delta_{\tilde{c}}^{G}=\sum_{i=1}^{N}I(\alpha_{i}=\alpha_{\tilde{c}})+\delta_{0\tilde{c}}^{G}. \end{array}$$

We set $\delta_{0}={\left(1,\cdots ,1\right)}_{L}$ in our study, following the setting in Culpepper (2015).

Thirdly, the prior of $\alpha_{i}$ follows a categorical distribution with parameter π under structure $\backslash textG_{null}$ :

(13) $$\begin{array}{c} p\left(\alpha_{i}|\pi \right)=\prod \pi_{c}^{I(\alpha_{i}=\alpha_{c})},c=0,\cdots ,C-1. \end{array}$$

Similar to parameter π , the prior of $\alpha_{i}$ also needs to be adjusted according to the hierarchy structure G . Accordingly, the prior of $\alpha_{i}$ follows a collapsed categorical distribution with parameter $\pi_{G}$ under structure G:

(14) $$\begin{array}{c} p\left(\alpha_{i}|\pi_{G}\right)=\prod_{\tilde{c}\in L_{G}}\pi_{\tilde{c}}^{I(\alpha_{i}=\alpha_{\tilde{c}})}. \end{array}$$

Based on the prior in Eq. (14) and the joint posterior distribution in Eq. (6), we can derive that posterior distribution of $\alpha_{i}$ is a categorical distribution with parameter $\rho_{i}^{G}=(\rho_{i0}^{G},\cdots ,\rho_{iC_{G}-1}^{G})$ as follows:

(15) $$\begin{array}{c} \alpha_{i}|\backslash textbfY,s,g,\pi_{G},\text{Q},\text{G}∼\backslash operatornameCategorical(\rho_{i}^{G}), \end{array}$$

where

$$\begin{array}{c} \rho_{i\tilde{c}}^{G}\propto \pi_{\tilde{c}}^{G}\prod_{j=1}^{J}{\left(g_{j}^{1-\eta_{\tilde{c}j}}{\left(1-s_{j}\right)}^{\eta_{\tilde{c}j}}\right)}^{y_{ij}}{\left({\left(1-g_{j}\right)}^{1-\eta_{\tilde{c}j}}s_{j}^{\eta_{\tilde{c}j}}\right)}^{1-y_{ij}}. \end{array}$$

Fourthly, as for the prior of the Q -matrix, we adopted the approach in Chung (2019) by introducing additional parameters for each Q-matrix entry and made corresponding adjustments. With *K* attributes, there are $H=2^{K}-1$ possible q -vector patterns for each item under structure $\backslash textG_{null}$ , which is denoted as $\epsilon_{H\times K}={\left(\epsilon_{hk}\right)}_{H\times K}$ . Suppose $q_{j}$ following a categorical distribution with parameter $\theta_{0j}=(\theta_{0j1},\cdots ,\theta_{0jH})$ :

(16) $$\begin{array}{c} p(q_{j}|\theta_{0j})\propto \prod_{h=1}^{H}\theta_{0jh}^{I(q_{j}=\epsilon_{h})}, \end{array}$$

$$\begin{array}{c} \theta_{0j}=g(\Phi_{j}),\sim \sim \Phi_{j}={\left(\phi_{j1},\cdots ,\phi_{jH}\right)}^{\text{T}}, \end{array}$$

where $\phi_{jh}=(\phi_{jh1},\cdots ,\phi_{jhK}),h\in (1,\cdots ,H)$ . We define $\phi_{jhk}=P(q_{jk}=1)$ and the prior for $\phi_{jhk}$ is

$$\begin{array}{c} \phi_{jhk}∼\backslash operatornameBeta(1,1). \end{array}$$

Therefore, the conditional posterior for each element $\phi_{jhk}$ is distributed as

(17) $$\begin{array}{c} \phi_{jhk}|q_{jk}∼\backslash operatornameBeta\left(1+q_{jk},2-q_{jk}\right). \end{array}$$

Moreover, the prior for $\theta_{0j}$ is then modeled as

(18) $$\begin{array}{c} \begin{array}{ccc} \backslash boldsymbol & \_ & \theta 0j=g(\_\Phi j)\\ = & ( & g(\_\phi j1),\cdots ,g(\_\phi jH)\propto (\prod_{k=1}^{K}\phi_{j1k}^{\epsilon_{1k}}{\left(1-\phi_{j1k}\right)}^{1-\epsilon_{1k}},\cdots ,\prod_{k=1}^{K}\phi_{jhk}^{\epsilon_{hk}}\\ \;\times {\left(1-\phi_{jhk}\right)}^{1-\epsilon_{hk}},\cdots ,\prod_{k=1}^{K}\phi_{jHk}^{\epsilon_{Hk}}{\left(1-\phi_{jHk}\right)}^{1-\epsilon_{Hk}}) & . \end{array} \end{array}$$

Considering that in the DINA model, restricting α is equivalent to restricting the Q -matrix, we do not impose any additional structural constraints on the Q -matrix during its update process. Therefore, based on the prior in Eq. (18) and the joint posterior distribution in Eq. (6), we can derive that posterior distribution of $q_{j}$ is a categorical distribution with parameter $\theta_{j}=(\theta_{j1},\cdots ,\theta_{jH})$ as follows:

(19) $$\begin{array}{c} q_{j}|\backslash textbfY,s,g,\pi ,\text{G}∼\backslash operatornameCategorical(\theta_{j}), \end{array}$$

where

(20) $$\begin{array}{c} \theta_{jh}=\frac{\gamma_{jh}}{\sum_{h=1}^{H}\gamma_{jh}}, \end{array}$$

(21) $$\begin{array}{c} \gamma_{jh}=p(\phi_{jh})\prod_{i=1}^{N}{\left(g_{j}^{1-\eta_{ij}}{\left(1-s_{j}\right)}^{\eta_{ij}}\right)}^{y_{ij}}{\left({\left(1-g_{j}\right)}^{1-\eta_{ij}}s_{j}^{\eta_{ij}}\right)}^{1-y_{ij}}. \end{array}$$

Finally, for the hierarchical structure G , we adopt a uniform prior, which means for any two possible transitive reduction structure G and $\backslash textG^{*}$ , we have $p(\text{G})=p(\backslash textG^{*})$ . As we are not able to get the closed form of the posterior distribution of G, similar to Chen and Wang (2023), the MH sampling method is utilized to update G . First, we need to sample a new structure $\backslash textG^{*}$ . Note that the new $\backslash textG^{*}$ can only be obtained by either removing an existing edge or adding an edge to the current structure G . Denote *p* as the probability of adding an edge, indicating 1−p as the probability of removing an edge. We first use the probability *p* to determine whether the new structure $\backslash textG^{*}$ involves adding or removing an edge, and then randomly generate a possible $\backslash textG^{*}$ . It is worth to note that this sampling procedure only exists between transitive reduction G s, meaning that each time a new $\backslash textG^{*}$ is sampled, it must have a different reachability matrix from G . According to this rule, each time an edge is added, it must be selected from the edges between two attributes that do not have any direct or indirect relationship. Such a pair of attributes can be found through 0 s in the off-diagonal entries in the reachability matrix. Let $T\left(\text{G},\backslash textG^{*}\right)$ denote the transition probability from G to the proposed new $\backslash textG^{*}$ . Define

(22) $$\begin{array}{c} T\left(\text{G},\backslash textG^{*}\right)=\frac{1}{2\times \text{numberofpairsofnon−connectednodes}}, \end{array}$$

when adding an edge, and

(23) $$\begin{array}{c} T\left(\text{G},\backslash textG^{*}\right)=\frac{1}{\text{numberofexistingedges}}, \end{array}$$

when removing an edge. Then the accept ratio of $\backslash textG^{*}$ is

(24) $$\begin{array}{c} \begin{array}{cccc} r\\ = & \backslash min & \backslash lbrace1,\frac{p\left(\backslash textG^{*}|\backslash textbfY,s,g,A,Q\right)T\left(\backslash textG^{*},\text{G}\right)}{p\left(\text{G}|\backslash textbfY,s,g,A,Q\right)T\left(\text{G},\backslash textG^{*}\right)}\backslash rbra & ce\\ = & \backslash min & \backslash lbrace1,\frac{p\left(\backslash textbfY|\backslash textG^{*},s,g,A,\text{Q}\right)p\left(\backslash textG^{*}\right)T\left(\backslash textG^{*},\text{G}\right)}{p(\backslash textbfY|\text{G},s,g,A,\text{Q})p(G)T\left(\text{G},\backslash textG^{*}\right)}\backslash rbra & ce\\ = & \backslash min & \backslash lbrace1,\frac{p\left(\backslash textbfY|\backslash textG^{*},s,g,A,Q\right)T\left(\backslash textG^{*},\text{G}\right)}{p(\backslash textbfY|\text{G},s,g,A,\text{Q})T\left(\text{G},\backslash textG^{*}\right)}\backslash rbra & ce. \end{array} \end{array}$$

Then, a random number is drawn from a uniform distribution and compared to the acceptance ratio *r* to determine whether to accept the proposed $\backslash textG^{*}$ .

Our proposed MH within Gibbs sampling procedure is summarized in Table 1, and *T* represent the length of the Markov chain. The prespecified probability *p* can be adjusted to improve the acceptance ratio of the newly proposed structure $\backslash textG^{*}$ . In this article, we set p=0.5 .

**Table 1:**
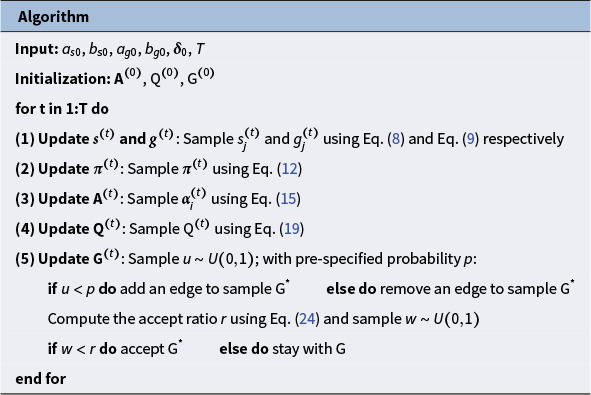
MH-Gibbs sampling procedure Table 1 long description.

## 4 Mini-batch and final estimation for model parameters

**Figure 2:**
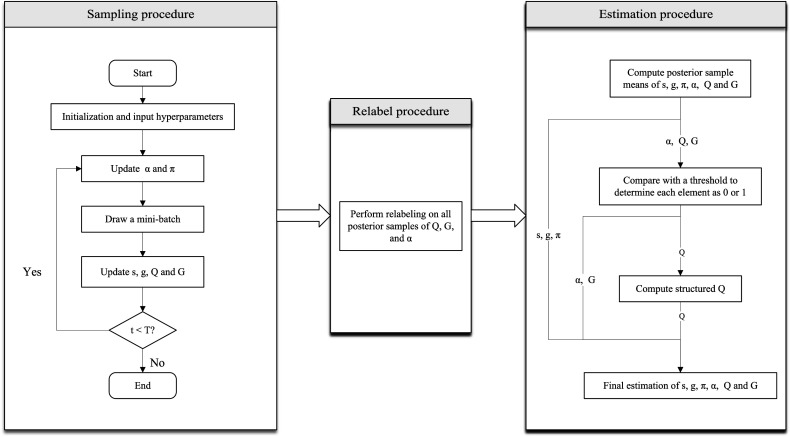
The overview of the complete Bayesian estimation procedure, including the sampling procedure, relabeling procedure, and final parameter estimation. Figure 2 long description.

In this section, we summarize the implementation of the proposed algorithm, including the use of mini-batch method in each iteration, addressing the potential label switch issue, and the way to obtain the final estimation for two latent structures and model parameters after the sampling procedure. The overview of the complete procedure from sampling procedure to final parameter estimation is provided in Figure 2. We provided details in the rest of this section.

### 4.1 Mini-batch method in sampling procedure

We propose to use the mini-batch method, where, during each iteration of the sampling algorithm (Table 1), we randomly select a subset of samples to update s , g , Q , and G . Note that α and π cannot be updated using sub-samples, as they are parameters specific to each individual sample. The mini-batch method is a widely used technique in machine learning, commonly applied in stochastic gradient descent. The method works by updating model parameters using only a subset of the samples during each iteration. In recent years, the mini-batch method has also found applications in the Bayesian field, such as in MH (Wu et al., 2022), Gibbs sampling (De Sa et al., 2018), Hamiltonian Monte Carlo (HMC) (Zou & Gu, 2021), and variational Bayes (Hoffman et al., 2013). This approach offers two main advantages. First, it improves the computational efficiency of the algorithm. Second, the mini-batch method introduces some noise, thereby increasing the diversity of the sampling and helping to prevent rapid convergence to local optima.

When implementing mini-batch with our proposed algorithm, one needs to determine the mini-batch size. Common practice in machine learning involves selecting batch sizes that are powers of two, such as 32, 64, 128, or 256, due to their compatibility with CPU and GPU memory architectures (Keskar et al., 2016). Keskar et al. (2016) have discussed that large-batch sample size tend to converge to sharp minimizers, while small-batch sample size consistently converge to flatter minimizers. This suggests that using a larger batch size does not necessarily lead to better estimation performance. In contrast, a relatively smaller batch size may introduce more stochasticity, which can help the algorithm escape from local optima. Given these considerations, we will specifically explore the impact of different mini-batch size to the model estimation results in the simulation section.

### 4.2 Label switch

Before computing the final estimations from the sampling procedure, it is necessary to address the issue of label switching. In the context of Bayesian methods, label switching is a common concern that may arise both within a single Markov chain and across multiple chains. In this article, the unknown Q-matrix may lead to label switching issues among several attributes, which also impacts α and G . Therefore, it is crucial to impose a relabeling process on the iterative data. Denote the J×K×T -dimension Q as the iteration samples of Q. Following the relabeling method in Erosheva and Curtis (2017), we define $\bar{Q}$ as the average of the sampled Q , which serves as the first reference. For each $\backslash textQ^{\left(t\right)}\in Q$ , we leverage the Hungarian algorithm to address the relabeling issue. Specifically, we construct a K×K cost matrix $C^{\left(t\right)}=\{c_{kk'}^{\left(t\right)}\}$ , where

$$\begin{array}{c} c_{kk'}^{\left(t\right)}=\|q_{k'}^{\left(t\right)}-\bar{q_{k}}{\|}_{1},\;k,k'=1,\cdots ,K,\;t=1,\cdots ,T. \end{array}$$

Let P denote the set of all permutations of {1,⋯,K} . Then, the relabeled $\backslash textQ^{\left(t\right)}$ , denoted as $\backslash textQ_{R}^{\left(t\right)}$ , is obtained by solving the following assignment problem:

(25) $$\begin{array}{c} p^{*}=\backslash arg\min_{p\in P}\sum_{k=1}^{K}c_{k,p(k)}^{\left(t\right)}, \end{array}$$

(26) $$\begin{array}{c} \backslash textQ_{R}^{\left(t\right)}=\backslash textQ_{p^{*}}^{\left(t\right)},\;t=1,\cdots ,T. \end{array}$$

Note that the above steps need to be repeated. In other words, after obtaining the new relabeled $Q_{R}$ , we need to calculate the new average matrix $\bar{\_}QR$ and repeat the relabel procedure until $Q_{R}$ converge. In addition, the same permutation must be applied to both α and G simultaneously, as they are also subject to the same relabel issues involved in Q matrix.

### 4.3 Final estimation summary

#### The estimation of G

4.3.1

The estimation of attribute hierarchy is reflected through the estimation of the adjacency matrix G . We can use the posterior sample mean as a posterior summary statistic for G , denoted as $\hat{\text{G}}$ . As a result, each element $\hat{{\text{G}}_{kk'}}$ takes a value between 0 and 1, which summarizes the probability for a potential edge from attribute *k* to k' . If an explicit structure is needed for the interpretation of probable hierarchy relationships, a carefully chosen threshold can decide on the final structure. Here, we define a threshold *c*, referred to as the cut-off value, to make the final estimation of G . Specifically, for each element of $\hat{\text{G}}$ , if $\hat{{\text{G}}_{kk'}}<c$ , we set $\hat{{\text{G}}_{kk'}}=0$ ; otherwise, $\hat{{\text{G}}_{kk'}}=1$ . Chen and Wang (2023) found that for edges that do not exist and have no indirect relationships between attributes, the estimated values are close to 0. For non-existing edges with indirect relationships between attributes, the estimated values range between 0.2 and 0.4. The specific value of the cut-off value will be explored in the simulation studies.

#### The estimation of Q matrix

4.3.2

After estimating the hierarchical structure, we obtain the estimated Q-matrix through the following two steps. First, similar to the estimation process of $\hat{\text{G}}$ , we compute the posterior sample mean of the Q-matrix, denoted as $\hat{\text{Q}}$ , and determine each element as 0 or 1 based on a threshold of 0.5. Second, given the estimated hierarchical structure $\hat{\text{G}}$ , we construct a structured $\hat{\text{Q}}$ according to the reduced Q-matrix $\backslash textQ_{r}(\hat{\text{G}})$ , which serves as the final estimation of the Q-matrix.

#### 4.3.3 The other model parameters

For the parameters s , g , and π , we can use their posterior sample means as point estimates. And for the attribute profiles α , we compute the posterior means and then dichotomize each element using a threshold of 0.5.

## 5 Simulation studies

The purpose of this set of simulation studies is to evaluate the performance of the proposed MH-Gibbs algorithm in recovering DINA model parameters across the following factors: 1) sample size and test length, 2) mini-batch size, 3) cutoff values for G , 4) attribute profile distribution, and 5) different attribute hierarchy structures.

### 5.1 Design

We consider a set up that a total of K=4 attributes were assessed, and we fix the slipping and guessing parameters in a practical level as $s_{j}=g_{j}=0.2$ . The summary of the five factors considered and their corresponding level is presented in Table 2. These factors were fully crossed, yielding 40 conditions. We conducted four chains in each replication, with the chain length of 20,000 and the burn in value of 10,000, and each simulation repeated 50 times.

**Table 2:** Simulation factors and levels Table 2 long description.

Factor	Levels
Sample size and test length	( N=500 , J=20 )
	( N=1,000 , J=40 )
Minibatch size	$N_{1}=128$ and 256 for N=500
	$N_{1}=128,256$ , and 512 for N=1,000 .
Cutoff value	0.2, 0.3, and 0.4
Attribute profile distribution	Balanced and unbalanced
Attribute hierarchy	$\backslash textG_{1}$ , $\backslash textG_{2}$ , $\backslash textG_{3}$ , and $\backslash textG_{4}$ in Figure 1.

The two test lengths represent relatively short and medium-length assessments. Recognizing that estimating a high-dimensional, unknown Q-matrix necessitates a larger sample size for accuracy, we increased the sample size paired with the increase of test length in our simulation design. This adjustment reflects both practical considerations and the need for a sufficiently large sample size. The four hierarchical structures of the attributes, as well as studied in previous related work (Chen & Wang, 2023), were therefore also considered in our study.

As for the mini-batch size, as noted in Section 3.3, common practice in machine learning involves selecting batch sizes that are powers of two. To balance convergence speed and computational efficiency, we avoided batch sizes that are excessively small or large. Consequently, we chose batch sizes ranging from one-quarter to one-half of the total sample size. Specifically, for N=500 with J=20 , we selected batch sizes of 128 and 256; for N=1,000 with J=40 , we chose batch sizes of 128, 256, and 512.

Given the findings in Chen and Wang (2023), for edges that do not exist and have no indirect relationships between attributes, the estimated values are close to 0. For non-existing edges with indirect relationships between attributes, the estimated values range between 0.2 and 0.4. Therefore, when selecting the cut-off value, we choose 0.2, 0.3, and 0.4.

Finally, following the design in Chen and Wang (2023), we also considered a balanced and unbalanced attribute profile distribution. For the balanced distribution, the attribute profiles were randomly generated from one of its permissible patterns. For the unbalanced distribution, we first generated $\alpha^{*}i_{=}{(\alpha^{*}{i1}_{,}\cdots ,\alpha^{*}{ik}_{,}\cdots ,\alpha^{*}{iK}_{)}}^{\text{T}}$ from a multivariate normal distribution $\alpha^{*}i_{∼}\text{N}(0_{K},\Sigma_{K\times K})$ , where $0_{K}={\left(0,\cdots ,0\right)}_{K\times 1}^{\text{T}}$ and

$$\begin{array}{c} \Sigma_{K\times K}={\left[\begin{array}{ccccccc} 1 & \cdots & \sigma \vdots & \ddots & \vdots \sigma & \cdots & 1 \end{array}\right]}_{K\times K}, \end{array}$$

the off-diagonal elements of $\Sigma_{K\times K}$ are σ=0.5 . Then the relationships between the attribute profile $\alpha_{i}$ and $\alpha_{i}^{*}$ can be expressed as $\alpha_{ik}=1$ if $\alpha_{ik}^{*}>0$ and $\alpha_{ik}=0$ otherwise.

### Q -matrix design

5.2

The establishment of parameter identifiability is a prerequisite for the accurate estimation and reliable interpretation of CDMs. To this end, it is essential to impose a set of constraints on the Q -matrix. Previous studies have discussed the identifiability conditions of parameters in CDMs (Chen et al., 2015; Chen et al., 2018; Gu & Xu, 2023; Ma et al., 2023). In particular, Gu and Xu (2023) explicitly addressed the identifiability conditions when a hierarchical structure exists among attributes and the Q -matrix is unknown. Therefore, in this study, we followed the identifiability conditions proposed by Gu and Xu (2023) when generating the Q -matrix. Specifically, they defined the “densified” version $D^{\text{G}}(\text{Q})$ and the “sparsified” version $S^{\text{G}}(\text{Q})$ of the Q -matrix. The “densified” version $D^{\text{G}}(\text{Q})$ corresponds to what we referred to earlier as the structured Q -matrix in Section 2.2, which is defined as: for any $q_{j,k'}=1$ and k→k' , set $q_{j,k}=1$ . Conversely, the “sparsified” version $S^{\text{G}}(\text{Q})$ is defined as: for any $q_{j,k'}=1$ and k→k' , set $q_{j,k}=0$ . For example, under the structure $\backslash textG_{1}$ , given a *q*-vector q=(0,1,1,0) , the “densified” version $D^{\text{G}}(q)$ becomes (1,1,1,0) , whereas the “sparsified” version $S^{\text{G}}(q)$ becomes (0,0,1,0) . Moreover, under $G_{1}$ , these three versions are equivalent, that is,

$$\begin{array}{c} (0,1,1,0)\;\backslash stackrelG_{1}∼\;(1,1,1,0)\;\backslash stackrelG_{1}∼\;(0,0,1,0). \end{array}$$

Denote the Q -matrix contains the following two components:

Q=\codexFontBold_^QK×K0\codexFontBold_^Q(J−K)×K⋆.

Specifically, we generate the Q-matrix satisfying the following three identifiability conditions: (A) the sub-matrix $\backslash textQ^{0}$ is equivalent to the identity matrix $I_{K}$ , that is, $\backslash textQ^{0}\backslash stackrel\text{G}∼I_{K}$ ; (B) the $S^{\text{G}}(\text{Q})$ has at least three ones in each column; and (C) the $D^{\text{G}}\left(\backslash textQ^{⋆}\right)$ contains *K* distinct column vectors.

Moreover, once we generated Q -matrices that satisfied the identifiability conditions, we uniformly adopted their corresponding “densified” versions, that is, the structured Q-matrices. It is worth noting that during the 50 replications under each simulation condition, we generated 50 distinct Q-matrices. This design may reduce comparability across different conditions. However, it allows us to better evaluate the performance and robustness of our algorithm across different Q-matrices under each condition.

### 5.3 Evaluation criteria

We evaluate the proposed algorithm through the following perspectives. First, the potential scale reduction factor (PSRF), denoted as $\hat{R}$ (Gelman & Rubin, 1992), was used to evaluate the convergence of the Gibbs algorithm across different simulation conditions. When the $\hat{R}$ is less than 1.1, the Markov chain is considered to have reached convergence. For each replication, four Markov chains were run. The chain that achieved the lowest deviance information criterion (DIC; Spiegelhalter et al., 2002), a widely used model selection criterion in the Bayesian framework, was considered the best and was selected for subsequent analysis.

Second, we used the average bias and root mean squared error (RMSE) to evaluate the accuracy of the estimation for the item parameters s , g , and attribute profile membership parameter π . These evaluation indexes are defined as

$$\begin{array}{cccc} & \backslash operatornameABias(s)=\frac{1}{J}\sum_{j=1}^{J}\frac{1}{R}\sum_{r=1}^{R}\left({\hat{s_{j}}}^{\left(r\right)}-s_{j}\right),\;\backslash operatornameARMSE(s)=\frac{1}{J}\sum_{j=1}^{J}\sqrt{\frac{1}{R}\sum_{r=1}^{R}{\left({\hat{s_{j}}}^{\left(r\right)}-s_{j}\right)}^{2}}, & \backslash operatornameABias(g)=\frac{1}{J}\sum_{j=1}^{J}\frac{1}{R}\sum_{r=1}^{R}\left({\hat{g_{j}}}^{\left(r\right)}-g_{j}\right),\;\backslash operatornameARMSE(g)=\frac{1}{J}\sum_{j=1}^{J}\sqrt{\frac{1}{R}\sum_{r=1}^{R}{\left({\hat{g_{j}}}^{\left(r\right)}-g_{j}\right)}^{2}}, & \backslash operatornameABias(\pi )=\frac{1}{C}\sum_{c=1}^{C}\frac{1}{R}\sum_{r=1}^{R}\left({\hat{\pi_{c}}}^{\left(r\right)}-\pi_{c}\right),\;\backslash operatornameARMSE(\pi )=\frac{1}{C}\sum_{c=1}^{C}\sqrt{\frac{1}{R}\sum_{r=1}^{R}{\left({\hat{\pi_{c}}}^{\left(r\right)}-\pi_{c}\right)}^{2}}, \end{array}$$

where *R* represent the replication times, and $\hat{.^{\left(r\right)}}$ represent the estimated value of a parameter in the *r*th replication. In addition, we provide the 95% highest probability density interval (HPDI) of s , g , and π , which is the narrowest interval that contains 95% of the posterior probability, to access the uncertainty related to these parameter estimation.

To evaluate the recovery of attribute profile estimation, we adopted the pattern-wise agreement rate (PAR), $\text{PAR}=\frac{1}{N}\sum_{i=1}^{N}I(\hat{\alpha_{i}}=\alpha_{i})$ , and the attribute-wise agreement rate (AAR), defined as $\text{AAR}(k)=\frac{1}{N}\sum_{i=1}^{N}I(\hat{\alpha_{ik}}=\alpha_{ik}).$ Here, $\hat{\alpha_{i}}$ is the estimated value of $\alpha_{i}$ , and $\hat{\alpha_{ik}}$ is the estimated value of $\alpha_{ik}$ . Higher values of PAR and AAR indicate better recovery of attribute profile.

Third, to assess the recovery of Q-matrix, we calculate the Q-matrix recovery rate for each dimension ( QRR ) and the average Q-matrix recovery rate ( AQRR ) across the Q-matrix as follows:

$$\begin{array}{c} \text{QRR(k)}=\frac{1}{R}\sum_{r=1}^{R}\left(1-\frac{\sum_{j=1}^{J}\left|\hat{q_{jk}^{\left(r\right)}}-q_{jk}\right|}{J}\right),\text{AQRR}=\frac{1}{K}\sum_{k=1}^{K}\text{QRR(K)}, \end{array}$$

where r=1,2,⋯,R and $\hat{q_{jk}^{\left(r\right)}}$ is the estimated value of $q_{jk}$ in the *r*th replication. The higher value of AQRR and ORR indicates better recovery of Q -matrix. Additionally, we also report the number of times the overall Q -matrix was correctly estimated across 50 replication.

Finally, when evaluating the recovery of the hierarchical structure, we considered four criteria: the true positive rate (TPR) and the true negative rate (TNR), based on the adjacency matrix ( G -matrix), as well as the reachability TPR (RTPR) and the reachability TNR (RTNR), based on the reachability matrix (*R*-matrix):

$$\begin{array}{c} \backslash operatornameTPR=\frac{\#\backslash operatornameTrue\sim positive\sim edge}{\#\backslash operatornameTrue\sim positive\sim edge+False\sim negative\sim edge}, \end{array}$$

$$\begin{array}{c} \backslash operatornameTNR=\frac{\#\backslash operatornameTrue\sim negative\sim edge}{\#\backslash operatornameTrue\sim negative\sim edge+False\sim positive\sim edge}, \end{array}$$

$$\begin{array}{c} \backslash operatornameRTPR=\frac{\#\backslash operatornameTrue\sim reachability\sim positive\sim edge}{\#\backslash operatornameTrue\sim reachability\sim positive\sim edge+False\sim reachability\sim negative\sim edge}, \end{array}$$

$$\begin{array}{c} \backslash operatornameRTNR=\frac{\#\backslash operatornameTrue\sim reachability\sim negative\sim edge}{\#\backslash operatornameTrue\sim reachability\sim negative\sim edge+False\sim reachability\sim positive\sim edge}. \end{array}$$

TPR is the proportion of positive edges that are correctly estimated, TNR denotes the proportion of negative edges that are correctly estimated, RTPR measures the proportion of reachability positive edges that are correctly estimated, and RTNR refers to the proportion of reachability negative edges that are correctly estimated. The higher and closer to 1 value of TPR, TNR, RTPR, and RTNR indicate a better recovery of the hierarchical structure G . The number of times the overall G was correctly estimated across 50 replications was also reported.

### 5.4 Simulation results

#### 5.4.1 Convergence analysis

The results across all simulation conditions consistently indicated that the algorithm converged within 10,000 iterations. We report the simulation condition of N=500 , J=20 , and $N_{1}=256$ as an example in Appendix A of the Supplementary Material. Therefore, the following results are based the chain that has the smallest DIC among the four of the last 10,000 steps.

#### 5.4.2 Computational time

We report the average computation time (in seconds) across all replications under each simulation condition in Table 3. Algorithm was operated in R programming language (R Core Team, 2017) on a desktop computer equipped with Intel(R) Core(TM) i5-10400 CPU @ 2.90 GHz, 16 GB RAM. The two R packages “Rcpp” (Eddelbuettel & François, 2011) and “RcppArmadillo” (Eddelbuettel & Sanderson, 2014) were used to enhance the computational efficiency. From the result in Table 3, when N=500 , it takes within 6 minutes across all conditions, and it takes within 19 minutes across all conditions when N=1,000 .

**Table 3:** Average computation time of the proposed algorithm under each condition in seconds Table 3 long description.

	*N*	*J*	*K*	$N_{1}$	$\backslash textG_{1}$	$\backslash textG_{2}$	$\backslash textG_{3}$	$\backslash textG_{4}$
Balanced	500	20	4	128	165.0952	195.3910	223.8264	208.2667
	500	20	4	256	264.6138	296.7557	339.5586	261.4543
	1,000	40	4	128	586.5280	588.8976	656.1260	592.0493
	1,000	40	4	256	718.5412	687.6035	791.3040	724.0883
	1,000	40	4	512	985.2270	972.3729	1,078.9910	1,020.1100
Unbalanced	500	20	4	128	167.1727	173.9886	200.8386	196.8976
	500	20	4	256	251.8998	273.0060	339.1605	306.8017
	1,000	40	4	128	515.7451	536.0071	678.1804	576.9780
	1,000	40	4	256	626.4555	651.6926	807.8230	706.1459
	1,000	40	4	512	857.5112	898.6855	1,095.6220	967.4907

#### The impact of mini-batch sample size and cut-off value of G

5.4.3

We first explore how different levels of mini-batch size and cut off values of G impact the performance of the proposed Gibbs algorithm. The recovery results of the Q -matrix and the hierarchical structure G under different mini-batch sample sizes and cut-off values are documented in Appendix B of the Supplementary Material. Here, we summarize the optimal cut-off value and mini-batch size under various simulation conditions in Table 4. In this table, each cell lists the mini-batch size and cutoff value that resulted in the best recovery of Q and G in a specific simulation condition. For example, for the attribute hierarchy $\backslash textG_{1}$ and sample size is 500 (test length is 20), the mini-batch size of 256, and the cut off value of 0.2 or 0.3 result in the highest recovery of Q and the mini-batch size of 256 and the cut off value of 0.3 resulted in the best recovery of $\backslash textG_{1}$ . From this table, we can see that for the cut-off value, under the condition N=500 , the highest recovery accuracy for both Q and G occurs when the cut-off value is 0.2, while for N=1,000 , both 0.2 and 0.3 yield high accuracy. For the mini-batch sample size, when N=500 , the highest recovery accuracy for both Q and G occurs when $N_{1}=256$ . Among all eight conditions under N=500 , the optimal mini-batch sample size of $N_{1}=128$ appears only once, indicating that $N_{1}=256$ is clearly superior. When N=1,000 , $N_{1}=512$ outperforms $N_{1}=256$ only two times, indicating that the performance of $N_{1}=256$ and $N_{1}=512$ is similar. We speculate that this is because, with larger sample sizes, even if the mini-batch sample size is not very large, the continual sampling process allows the mini-batch samples to cover all the sample information since the ergodicity, leading to more accurate estimates. Based on these findings, we use mini-batch size $N_{1}=256$ when N=500 and $N_{1}=512$ for N=1,000 , and cut off value of 0.2 in the subsequent analysis.

**Table 4:** Summary of the optimal mini-bath size and cut-off value Table 4 long description.

According to the overall recovery of Q				
	N=500		N=1,000	
	Balanced	Unbalanced	Balanced	Unbalanced
$\backslash textG_{1}$	$N_{1}=256c=0.2/0.3$	$N_{1}=256c=0.3$	$N_{1}=512c=0.2/0.3$	$N_{1}=512c=0.2/0.3$
$\backslash textG_{2}$	$N_{1}=256c=0.2$	$N_{1}=256c=0.2/0.3$	$N_{1}=256c=0.2/0.3/0.4$	$N_{1}=512c=0.2/0.3$
$\backslash textG_{3}$	$N_{1}=128c=0.2$	$N_{1}=256c=0.2$	$N_{1}=256c=0.2/0.3/0.4$	$N_{1}=256/512c=0.2/0.3/0.4$
$\backslash textG_{4}$	$N_{1}=256c=0.2/0.3$	$N_{1}=256c=0.2$	$N_{1}=512c=0.2/0.3$	$N_{1}=256/512c=0.2/0.3$
Summary	$N_{1}=128(1\sim \text{time})/256(7\sim \text{times})$		$N_{1}=256(4\sim \text{times})/512(6\sim \text{times})$	
	c=0.2(7~times)/0.3(4~times)		c=0.2(8~times)/0.3(8~times)/0.4(3~times)	
According to the overall recovery of G				
	N=500		N=1,000	
	Balanced	Unbalanced	Balanced	Unbalanced
$\backslash textG_{1}$	$N_{1}=256c=0.3$	$N_{1}=256c=0.3$	$N_{1}=512c=0.3$	$N_{1}=512c=0.2/0.3$
$\backslash textG_{2}$	$N_{1}=256c=0.2$	$N_{1}=256c=0.2$	$N_{1}=256c=0.2/0.3/0.4$	$N_{1}=512c=0.2/0.3$
$\backslash textG_{3}$	$N_{1}=128/256c=0.2$	$N_{1}=256c=0.2$	$N_{1}=256c=0.2/0.3$	$N_{1}=256/512c=0.2/0.3$
$\backslash textG_{4}$	$N_{1}=256c=0.2/0.3$	$N_{1}=256c=0.2$	$N_{1}=512c=0.2$	$N_{1}=256/512c=0.2/0.3$
Summary	$N_{1}=128(1\sim \text{time})/256(8\sim \text{times})$		$N_{1}=256(4\sim \text{times})/512(6\sim \text{times})$	
	c=0.2(6~times)/0.3(3~times)		c=0.2(7~times)/0.3(7~times)/0.4(1~time)	

#### Recovery of G

5.4.4

Table 5 displays the recovery of the hierarchical structure G . Based on the results, we find that when N=500 , the proportion of accurately estimating G exceeds 80% across all replications and conditions, and this proportion increases to over 88% when N=1,000 . The TPR and TFR results indicate that, across all simulation conditions, the proposed algorithm achieves over 90% accuracy, except under $\backslash textG_{1}$ in unbalanced condition, in estimating the hierarchical structure edges between attributes, and the RTPR and RTFR results show that the accuracy in estimating direct or indirect dependencies between attributes is even higher, reaching over 95%. Moreover, Table 6 presents the average posterior sample mean of G ( $\hat{\text{G}}$ ) across 50 replications under the condition N=1,000 , J=20 , where the bold numbers indicate the true edges. The results show that our algorithm yields estimated values above 0.4 for the true edges between attributes. For non-existing edges but with indirect relationships between attributes, the estimated values range between 0.2 and 0.4. For edges that do not exist and have no indirect relationships between attributes, the estimated values are close to 0. This finding indicates that our method performs remarkably well in identifying the reachability relationships among attributes.

**Table 5:** Evaluate the recovery accuracy of the adjacency matrix G Table 5 long description.

	Balanced					
		TPR	TFR	RTPR	RTFR	$\hat{\text{G}}=\text{G}$
$\backslash textG_{1}$	N=500,J=20	0.900	0.972	0.986	0.967	44
	N=1,000,J=40	0.927	0.983	0.976	0.960	44
$\backslash textG_{2}$	N=500,J=20	0.985	0.997	0.996	0.997	49
	N=1,000,J=40	0.910	0.978	0.976	0.969	45
$\backslash textG_{3}$	N=500,J=20	0.973	0.995	0.989	0.991	48
	N=1,000,J=40	0.947	0.988	0.977	0.982	46
$\backslash textG_{4}$	N=500,J=20	1.000	1.000	1.000	1.000	50
	N=1,000,J=40	1.000	1.000	1.000	1.000	50
	Unbalanced					
		TPR	TFR	RTPR	RTFR	$\hat{\text{G}}=\text{G}$
$\backslash textG_{1}$	N=500,J=20	0.853	0.963	0.978	0.957	40
	N=1,000,J=40	0.987	0.997	0.996	0.993	49
$\backslash textG_{2}$	N=500,J=20	0.920	0.980	0.982	0.977	46
	N=1,000,J=40	0.980	0.995	0.996	0.994	49
$\backslash textG_{3}$	N=500,J=20	1.000	1.000	1.000	1.000	50
	N=1,000,J=40	1.000	1.000	1.000	1.000	50
$\backslash textG_{4}$	N=500,J=20	0.960	0.994	0.990	0.993	46
	N=1,000,J=40	1.000	1.000	1.000	1.000	50

*Note*: TPR and TFR are the true positive rate and true negative rate based on G-matrix; RTPR and RTFR are the true positive rate and true negative rate based on reachability matrix (R-matrix). The last column reports the number of times the entire G-matrix was recovered correctly.

**Table 6:** Estimated adjacency matrices $\hat{\text{G}}$ of the proposed algorithm for N=1,000 , J=20 condition Table 6 long description.

	Balanced					Unbalanced			
$\backslash textG_{1}$	0.000	**0.469**	0.376	0.304		0.000	**0.471**	0.377	0.298
	0.000	0.000	**0.473**	0.343		0.000	0.000	**0.473**	0.372
	0.000	0.000	0.000	**0.436**		0.000	0.000	0.000	**0.476**
	0.031	0.040	0.040	0.000		0.007	0.008	0.007	0.000
$\backslash textG_{2}$	0.000	**0.462**	**0.465**	0.300		0.000	**0.454**	**0.454**	0.299
	0.000	0.000	0.000	**0.431**		0.000	0.000	0.000	**0.472**
	0.000	0.000	0.000	**0.420**		0.000	0.000	0.000	**0.469**
	0.030	0.042	0.040	0.000		0.008	0.008	0.007	0.000
$\backslash textG_{3}$	0.000	**0.466**	**0.432**	**0.446**		0.000	**0.470**	**0.473**	**0.479**
	0.001	0.000	0.001	0.011		0.000	0.000	0.000	0.000
	0.000	0.026	0.000	0.010		0.000	0.000	0.000	0.000
	0.000	0.023	0.000	0.000		0.000	0.000	0.000	0.000
$\backslash textG_{4}$	0.000	**0.465**	**0.470**	0.372		0.000	**0.465**	**0.467**	0.372
	0.000	0.000	0.000	0.000		0.000	0.000	0.000	0.000
	0.000	0.000	0.000	**0.470**		0.000	0.000	0.000	**0.473**
	0.000	0.000	0.000	0.000		0.000	0.000	0.000	0.000

*Note*: The bolded entries in the matrix represent the real edges.

In cases where the hierarchical structure was incorrectly estimated, we found that the primary sources of error were the misestimation of directional relationships among attributes and, in some instances, the failure to identify certain edges. Notably, the recovery of structure $\backslash textG_{1}$ was less ideal compared to the other structures when the sample size was 500. Upon further examination, we observed that most of the estimation failures were caused by incorrect directionality within the attribute hierarchy. For instance, in one replication, the true structure 1→2→3→4 was incorrectly estimated as 1→2→4→3 , in which the direction between attributes 3 and 4 was reversed. Similar issues were found under structure $\backslash textG_{3}$ in the balanced condition, where the true edges 1→2 , 1→3 , and 1→4 were sometimes incorrectly estimated as 2/3/4→1 . Moreover, under structure $\backslash textG_{3}$ , many incorrect estimations involved missing edges connected to attribute 1. Regardless of which edge was missing, this consistently led to the lowest QRR value for the first attribute.

In addition, the overall recovery accuracy was the lowest under the linear structure $\backslash textG_{1}$ . To further investigate this, we conducted an additional analysis of the simulation data and found two main contributing factors: (1) a small proportion of items measuring certain attributes in the Q -matrix and (2) an unbalanced distribution of examinees across the permissible attribute mastery patterns. Specifically, under both balanced and unbalanced conditions, estimation failures often occurred when fewer than 15% of items measured the fourth attribute. Moreover, in the unbalanced condition with N=500 , J=20 , the examinees with mastery patterns (1,0,0,0) and (1,1,0,0) accounted for less than 10%, sometimes even below 5%, leading to insufficient information for accurate estimation. When the sample size increased to N=1,000 , this issue was largely mitigated. These findings indicate that both limited item coverage for certain attributes and an unbalanced distribution of attribute mastery patterns can substantially reduce estimation accuracy.

Finally, an interesting finding is that, although the recovery of G shows similar overall accuracy under both balanced and unbalanced attribute profile conditions, there are subtle differences. For example, under a sample size of 500 and test length of 20, the recovery rate under the balanced condition is higher for all structures except $\backslash textG_{3}$ . However, when the sample size increases to 1,000 and test length increases to 40, the recovery rate under the unbalanced condition becomes higher. Additionally, under the balanced attribute profile condition, increasing the sample size and test length does not necessarily improve the recovery of G . In contrast, under the unbalanced attribute profile condition, the increase in sample size and test length lead to better recovery of G .

We conjecture that under the balanced condition, the sample of each attribute profile is already relatively well-balanced even at smaller sample sizes, leading to relatively good recovery rates. Therefore, increasing the sample size from 500 to 1,000 does not result in a significant improvement. In the unbalanced condition, however, the imbalance in the sample of each attribute profile results in relatively poor estimation performance at smaller sample sizes. As the sample size increases, the sample of each attribute profile increases, leading to a noticeable improvement in recovery rates.

#### 5.4.5 Recovery of Q matrix

Table 7 presents the recovery accuracy of the Q -matrix. First, across all conditions, the percentage of replications that can fully recover the whole true Q matrix, ranging from 60\%(30/50) to 100\%(50/50) , and with mean of 92\% . Second, overall, the proposed algorithm generally resulted in the same or higher recovery of Q , calculated as the number of times the estimated Q -matrix matches the true Q -matrix. This improvement was observed with increasing test length and sample size across almost all conditions, except in the balanced condition with the hierarchical structure $\backslash textG_{2}$ . In this case, the number of correct Q estimations decreased from 48 to 45 when moving from N=500 , J=20 to N=1,000 , J=40 .

**Table 7:** Evaluate the recovery accuracy of Q -matrix Table 7 long description.

	Balanced						
		QRR1	QRR2	QRR3	QRR4	AQRR	$\hat{Q}=Q$
$\backslash textG_{1}$	N=500,J=20	1.000	1.000	1.000	0.930	0.983	44
	N=1,000,J=40	1.000	1.000	1.000	0.903	0.976	44
$\backslash textG_{2}$	N=500,J=20	1.000	1.000	1.000	0.984	0.996	48
	N=1,000,J=40	1.000	1.000	1.000	0.918	0.980	45
$\backslash textG_{3}$	N=500,J=20	0.984	0.993	1.000	0.991	0.992	46
	N=1,000,J=40	0.969	0.979	0.998	0.992	0.985	46
$\backslash textG_{4}$	N=500,J=20	1.000	1.000	1.000	0.991	0.998	49
	N=1,000,J=40	1.000	1.000	1.000	1.000	1.000	50
	Unbalanced						
		QRR1	QRR2	QRR3	QRR4	AQRR	$\hat{Q}=Q$
$\backslash textG_{1}$	N=500,J=20	0.996	0.951	1.000	0.953	0.975	30
	N=1,000,J=40	1.000	1.000	1.000	0.984	0.996	49
$\backslash textG_{2}$	N=500,J=20	0.991	0.997	0.996	0.950	0.984	44
	N=1,000,J=40	1.000	1.000	1.000	0.984	0.996	49
$\backslash textG_{3}$	N=500,J=20	1.000	1.000	1.000	1.000	1.000	50
	N=1,000,J=40	1.000	1.000	1.000	1.000	1.000	50
$\backslash textG_{4}$	N=500,J=20	0.989	0.999	0.982	0.993	0.991	45
	N=1,000,J=40	1.000	1.000	1.000	1.000	1.000	50

*Note*: QRR1–QRR4 represent the correct recovery rates for the four attributes, AQRR denotes the element-wise recovery rate of the Q-matrix, and the last column reports the number of times the entire Q-matrix was recovered correctly.

From the element-wise perspective, based on the AQRR value, the element-wise recovery rate was above 97% across all simulation conditions. In the balanced conditions, under structures $\backslash textG_{1}$ , $\backslash textG_{2}$ , and $\backslash textG_{3}$ , the AQRR value showed a slight decrease from N=500 , J=20 to N=1,000 , J=40 , while in other conditions, it showed a slight increase. The AQRR values are similar for sample sizes of 500 and 1,000. Similar to the overall recovery of Q , the element-wise recovery rate does not always improve with an increasing sample size. We speculate that this is because, although the sample size increased from 500 to 1,000, the test length also increased from 20 to 40, meaning the number of elements in Q that required estimation doubled thus increase the difficulty in estimation as well.

In addition, the recovery of Q , whether considering element-wise accuracy or overall accuracy, generally shows similar results and trends under both balanced and unbalanced conditions, with only slight differences. When N=1,000 , the overall number of correct recoveries of Q in the unbalanced condition is slightly higher than in the balanced condition.

Finally, given a sample size, the recovery of Q is similar across the four considered attribute hierarchical structures. However, in terms of the total number of correct recoveries for Q , the unbalanced condition with a sample size of 500 shows only 30 correct recoveries for $\backslash textG_{1}$ (linear structure), which is noticeably lower than in the other conditions. Nevertheless, the corresponding QARR values are similar to those for the other structures. This is mainly closely related to the recovery of G under that condition. Specifically, by comparing Tables 4 and 5, we found that the total number of correct estimations for Q generally aligns closely with that of the hierarchical structure G , with the correct estimations for Q being less than or equal to those for G . This suggests that if G is estimated correctly, there is a high likelihood that Q will also be accurately estimated. Conversely, if G is estimated incorrectly, Q is unlikely to be estimated correctly, since G directly influences the permissible patterns for Q . For unbalanced condition with a sample size of 500 under the hierarchical structure $\backslash textG_{1}$ , only 40 out of 50 replications generate correct estimations of the overall G , which is lower than those from other conditions, thus also leads to the undesired recovery of Q .

#### 5.4.6 Recovery of item parameters and attribute profile

Table 8 presents the ARMSE and ABias of the item parameters s and g , as well as the class membership parameter π . Since the true values of s and g are identical across all items, we further report the average HPDI (AHPDI) for s and g , defined as the mean of the lower and upper bounds of the HPDIs across all items and over 50 replications. And we also report the AHPDI for π over 50 replications in the figures in Appendix C of the Supplementary Material. The results indicate that the parameters s , g , and π exhibit good performance in terms of bias, with bias values close to zero under all simulation conditions. Across all hierarchical structures, as the sample size and test length increase from N=500,J=20 to N=1,000,J=40 , the average RMSE of s and g shows a declining trend, indicating that the accuracy of item parameter estimation improves with larger sample sizes. Simultaneously, the 95% AHPDI for parameters s , g , and π becomes narrower, further demonstrating that parameter estimation accuracy benefits from larger samples. Furthermore, Table 9 reports the recovery accuracy of the attribute profiles α . The values of AAR and PAR also increase as the sample size grows, suggesting that the recovery accuracy of α improves with larger sample sizes.

**Table 8:** Evaluate the estimation accuracy for the item parameters s , g , and the class membership probability parameter π Table 8 long description.

Balanced						
		$\backslash text{ARMSE}_{s}(\backslash text{ABias}_{s})$	$95\backslash \%\backslash text{AHPDI}_{s}$	$\backslash text{ARMSE}_{g}(\backslash text{ABias}_{g})$	$95\%\backslash text{AHPDI}_{g}$	$\backslash text{ARMSE}_{\pi }(\backslash text{ABias}_{\pi })$
$\backslash textG_{1}$	N=500,J=20	0.038(0.009)	[0.116, 0.305]	0.033(0.007)	[0.110, 0.308]	0.023(0.000)
	N=1,000,J=40	0.032(0.008)	[0.143, 0.274]	0.020(0.003)	[0.137, 0.270]	0.026(0.000)
$\backslash textG_{2}$	N=500,J=20	0.037(0.012)	[0.112, 0.316]	0.031(0.007)	[0.109, 0.309]	0.018(0.000)
	N=1,000,J=40	0.033(0.011)	[0.140, 0.287]	0.021(0.004)	[0.138, 0.274]	0.028(0.000)
$\backslash textG_{3}$	N=500,J=20	0.036(0.008)	[0.099, 0.323]	0.058(0.024)	[0.116, 0.337]	0.022(0.000)
	N=1,000,J=40	0.029(0.011)	[0.135, 0.288]	0.040(0.013)	[0.137, 0.274]	0.020(0.000)
$\backslash textG_{4}$	N=500,J=20	0.037(0.010)	[0.109, 0.315]	0.037(0.010)	[0.109, 0.315]	0.014(0.000)
	N=1,000,J=40	0.022(0.006)	[0.133, 0.303]	0.022(0.004)	[0.143, 0.289]	0.006(0.000)
Unbalanced						
		$\backslash text{ARMSE}_{s}(\backslash text{ABias}_{s})$	$95\backslash \%\backslash text{AHPDI}_{s}$	$\backslash text{ARMSE}_{g}(\backslash text{ABias}_{g})$	$95\backslash \%\backslash text{AHPDI}_{g}$	$\backslash text{ARMSE}_{\pi }(\backslash text{ABias}_{\pi })$
$\backslash textG_{1}$	N=500,J=20	0.025(0.003)	[0.130, 0.278]	0.038(0.010)	[0.095, 0.330]	0.013(0.000)
	N=1,000,J=40	0.016(0.002)	[0.151, 0.255]	0.024(0.003)	[0.127, 0.281]	0.007(0.000)
$\backslash textG_{2}$	N=500,J=20	0.023(0.004)	[0.132, 0.279]	0.065(0.025)	[0.086, 0.375]	0.017(0.000)
	N=1,000,J=40	0.017(0.002)	[0.150, 0.255]	0.022(0.002)	[0.130, 0.277]	0.007(0.000)
$\backslash textG_{3}$	N=500,J=20	0.030(0.007)	[0.116, 0.302]	0.030(0.005)	[0.112, 0.302]	0.011(0.000)
	N=1,000,J=40	0.019(0.003)	[0.141, 0.266]	0.019(0.001)	[0.139, 0.265]	0.004(0.000)
$\backslash textG_{4}$	N=500,J=20	0.028(0.006)	[0.122, 0.294]	0.078(0.031)	[0.103, 0.367]	0.015(0.000)
	N=1,000,J=40	0.018(0.003)	[0.146, 0.261]	0.020(0.002)	[0.134, 0.271]	0.003(0.000)

*Note*: ARMSE, ABias, and AHPDI represent the average RMSE, average bias, and average high density posterior interval across all items or all latent classes.

**Table 9:** Evaluate the estimation accuracy for the attribute profile α Table 9 long description.

	Balanced							Unbalanced				
		AAR1	AAR2	AAR3	AAR4	PAR		AAR1	AAR2	AAR3	AAR4	PAR
$\backslash textG_{1}$	N=500,J=20	0.968	0.959	0.964	0.929	0.839		0.976	0.981	0.978	0.949	0.897
	N=1,000,J=40	0.990	0.989	0.989	0.927	0.899		0.995	0.996	0.995	0.983	0.970
$\backslash textG_{2}$	N=500,J=20	0.961	0.948	0.947	0.962	0.837		0.978	0.965	0.968	0.932	0.863
	N=1,000,J=40	0.985	0.984	0.983	0.925	0.8820		.994	0.993	0.994	0.983	0.966
$\backslash textG_{3}$	N=500,J=20	0.933	0.930	0.920	0.917	0.749		0.962	0.939	0.946	0.935	0.824
	N=1,000,J=40	0.952	0.955	0.971	0.966	0.858		0.988	0.981	0.984	0.978	0.938
$\backslash textG_{4}$	N=500,J=20	0.963	0.934	0.937	0.946	0.811		0.967	0.939	0.950	0.937	0.826
	N=1,000,J=40	0.988	0.978	0.981	0.983	0.934		0.993	0.988	0.990	0.988	0.963

*Note*: AAR1 represents the correct classification rate for the first attribute, AAR2 for the second attribute, AAR3 for the third attribute, and AAR4 for the fourth attribute. PAR stands for the pattern-wise agreement rate.

## 6 Real data application

In this section, we demonstrated the proposed algorithm through a data set collected from the Examination for the Certificate of Proficiency in English (ECPE). ECPE is a test developed and scored by the English Language Institute of the University of Michigan. This data set is based on 2,922 examinees’s responses to 28 multiple-choice questions. Three skills are designed to be measured in ECPE: (1) knowledge of morphosyntactic rules, (2) cohesive rules, and (3) lexical rules (Buck & Tatsuoka, 1998; Henson & Templin, 2007).

### 6.1 Analysis procedures

Given the total sample size, we chose three mini-batch sample sizes $N_{1}=512$ , 1,024, and 1,500 to implement the proposed algorithm. A total of four Markov chains were conducted for each selected batch size, and the chain length was set as 20,000 and burn in value is 10,000. In addition, the prior for each parameter is consistent with the settings used in the simulation study. Our purpose is to investigate whether the proposed algorithm can successfully recover the linear hierarchical structure 3→2→1 among the three skills in the ECPE test, as identified in previous research (Templin & Bradshaw, 2014; Wang & Lu, 2021).

### 6.2 Results

First of all, the $\hat{R}$ values of s and g under $N_{1}=512$ condition are 1.000 and 1.000, respectively, under $N_{1}=1,024$ condition are 1.020 and 1.040, respectively, and under $N_{1}=1,500$ condition are 1.010 and 1.020, respectively. All $\hat{R}$ values are all below 1.1, indicating that the Markov chains have converged. Table 10 shows the $\hat{G}$ , and the DIC values under different mini-batch conditions.

**Table 10:** Estimated adjacency matrix $\hat{\text{G}}$ and the DIC value under different mini-batch conditions Table 10 long description.

	$N_{1}=512$				$N_{1}=1,024$				$N_{1}=1,500$		
$\hat{\text{G}}$	0.000	0.053	0.052		0.000	0.029	0.006		0.000	0.065	0.000
	**0.408**	0.000	0.170		**0.212**	0.000	0.017		**0.426**	0.000	0.019
	**0.374**	**0.364**	0.000		**0.340**	**0.362**	0.000		**0.407**	**0.489**	0.000
DIC	83,271.820				82,280.660				82,837.410		

*Note*: The bold values represent the edges that are considered to exist with strong statistical evidence.

#### 6.2.1 The estimated G

In terms of $\hat{G}$ values, the estimated values for the true edges in real data are somehow lower than those in the simulations. For example, the estimated value for edge 2→1 is only 0.212 (i.e., $\hat{G}(2,1)=0.212$ ) under the $N_{1}=1,024$ condition. Yet results also show that the estimates for non-existent edges are close to zero, that is, all edges other than 2→1 , 3→1 , and 3→2 have estimates close to zero, indicating their absence. In addition, the results under both $N_{1}=512$ and $N_{1}=1,500$ consistently reveal the same hierarchical structure. Accordingly, our estimated hierarchical structure is 3→2→1 which is consistent with previous studies mentioned above. Moreover, based on the DIC values, we selected the mini-batch size of 1,024, which provided the minimum DIC value, for further analysis.

#### 6.2.2 The estimated Q

Finally, based on the result under $N_{1}=1,024$ condition, Table 11 presents both the expert labeled structured Q-matrix and our estimated structured Q-matrix, with highlighted elements indicating discrepancies between them. The differences occurred in items 9, 17, and 21, and mostly on the first two attributes. This is because attribute 3 is a prerequisite for attributes 1 and 2, and thus all its values are 1. The element-wise agreement of expert labeled Q and our estimated Q is 0.952, indicating that the element-wise consistency rate with the expert-labeled Q-matrix reaches 95.2%, further demonstrating the effectiveness of our algorithm.

**Table 11:** The expert labeled Q -matrix and the estimated structured Q -matrix of from ECPE data Table 11 long description.

Item	Expert labeled structured \textbfQ			Estimated structured \textbfQ		
1	1	1	1	1	1	1
2	0	1	1	0	1	1
3	1	1	1	1	1	1
4	0	0	1	0	0	1
5	0	0	1	0	0	1
6	0	0	1	0	0	1
7	1	1	1	1	1	1
8	0	1	1	0	1	1
9	0	0	1	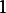	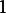	1
10	1	1	1	1	1	1
11	1	1	1	1	1	1
12	1	1	1	1	1	1
13	1	1	1	1	1	1
14	1	1	1	1	1	1
15	0	0	1	0	0	1
16	1	1	1	1	1	1
17	0	1	1	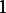	1	1
18	0	0	1	0	0	1
19	0	0	1	0	0	1
20	1	1	1	1	1	1
21	1	1	1	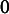	1	1
22	0	0	1	0	0	1
23	0	1	1	0	1	1
24	0	1	1	0	1	1
25	1	1	1	1	1	1
26	0	0	1	0	0	1
27	1	1	1	1	1	1
28	0	0	1	0	0	1

*Note*: The highlighted elements represent the differences between the true structured Q-matrix and the estimated structured Q-matrix.

## 7 Discussion

In this study, we proposed a Bayesian estimation method for the simultaneous estimation of the Q-matrix, the attribute hierarchy, and the parameters of the DINA model when both the Q-matrix and the attribute hierarchy are unknown. To implement this, we developed an MH-Gibbs sampling algorithm. To enhance computational efficiency, we incorporated a mini-batch approach and investigated the impact of mini-batch sample size on estimation accuracy. A series of simulation studies were conducted to evaluate the performance of the proposed algorithm across various conditions, including mini-batch sample size, cutoff values for G, different attribute hierarchy structures, sample size, and test length. Overall, the simulation results support the accuracy and computational efficiency of our algorithm. Given a specified cut-off value and mini-batch size, the proposed algorithm demonstrates strong performance across most conditions. Except for the $\backslash textG_{1}$ structure with N=500 under the unbalanced condition, the overall recovery rates for both the Q-matrix and the adjacency matrix G exceed 88%, with several conditions achieving a perfect 100% recovery. At the element-wise level, the algorithm achieves recovery rates above 97.5% for the Q-matrix and over 95% for the adjacency matrix G. These results underscore the robustness and reliability of the proposed estimation method in jointly recovering both the Q-matrix and the attribute hierarchy structure, even when both are unknown. A real data application further demonstrates the validity of the estimated latent structures, G and Q, obtained from the proposed method.

We next provide further discussion on the major findings from our simulation studies and real data application, which may offer practical guidelines for applying the proposed algorithm to datasets with varying structures. First, regarding the selection of mini-batch size, our results suggest that using a mini-batch size between one-quarter and one-half of the total sample size generally yields accurate parameter estimates. However, we recommend that users test multiple mini-batch sizes when analyzing real data and select the optimal setting based on model fit indices such as the DIC. This recommendation stems from the fact that our simulations and empirical analyses were conducted on datasets with sample sizes ranging from approximately 500 to 3,000, and performance may vary in other contexts.

Next, regarding the selection of the cut-off value for G, our simulation and real data analyses suggest that a threshold between 0.2 and 0.3 generally yields accurate estimation of the attribute hierarchy under the DINA model. We observed that with larger sample sizes, the estimated values for existing edges tend to be higher, indicating increased precision as more information becomes available. Importantly, even when the estimated values for connected (i.e., direct and indirect) edges are relatively small (around 0.2), those for non-connected edges are typically close to zero. This distinction allows for reliable differentiation between edges with and without dependencies. While we used a cut-off of 0.2 in this study, we caution that this is not a universal threshold; users are encouraged to determine an appropriate cut-off based on the characteristics of their own data.

Finally, an interesting finding is that although G and the corresponding Q in some simulation conditions exhibit the least desirable recovery when the sample size is 500, the item parameters and attribute profiles under this condition still perform similarly to those of the other attribute hierarchical structure. This is consistent with the finding from Wang (2018) that the classification results from CDM can maintain at relatively high accuracy for a missspecified Q -matrix that satisfy certain conditions.

We conclude by discussing several limitations of the current study and potential directions for future research. First, to determine the final structure of the Q-matrix, we used a fixed threshold of 0.5. However, in the real-world ECPE dataset, we observed that some estimated values were very close to this cutoff (e.g., between 0.49 and 0.51), raising concerns about the appropriateness of using a strict threshold. Identifying a more flexible or data-driven approach for determining this threshold could improve Q-matrix recovery accuracy and represents an important area for future investigation.

Second, in this article, we set cut-off value as 0.2 on the ECPE data set since the posterior estimates of the adjacency matrix G clearly distinguish between existing and non-existing edges. However, this may not hold for other data sets. A straightforward strategy we currently consider is to try several different cut-off values, fit the model under each, and then use model selection criteria such as DIC to determine the most appropriate attribute hierarchy structure. However, constructing a theoretically grounded, data-driven procedure for determining the cut-off value would be an important direction for our future research, which could further improve and refine our proposed method.

Third, although many CDMs have been developed—such as the DINA model (Junker & Sijtsma, 2001), the deterministic inputs, noisy “or” gate (DINO) model (Templin & Henson, 2006), the generalized DINA (GDINA) model (De La Torre, 2011), the log-linear cognitive diagnosis model (LCDM) (Henson et al., 2009), and the general diagnostic model (GDM) (Von Davier, 2008)—this study focuses on developing an estimation method based on the DINA model. The DINA model is one of the most widely used CDMs and has gained substantial popularity in educational and psychological measurement due to its interpretability and simplicity. We plan to extend our algorithm to accommodate more general CDMs, such as the LCDM, GDM, and GDINA models. However, such an extension may involve the following two main challenges. First, when an attribute hierarchy is present, under the DINA model’s response mechanism, it is sufficient to impose constraints on either the attribute profiles or the Q-matrix. However, this property may not hold for other CDMs. Second, in the DINA model, the item parameters have explicit posterior forms, which allows the algorithm to be implemented within a Gibbs sampling framework. In contrast, this property may not be satisfied in other CDMs, posing another challenge for extending our method.

Additionally, the current study focused only on a fixed number of attributes *K*. Therefore, extending our method to scenarios where *K* is unknown would be an important direction for future research. Finally, although our use of the mini-batch approach significantly improves computational efficiency, the average runtime remains between 14 and 18 minutes for a sample size of N=1,000 . Thus, we aim to further optimize our implementation to improve scalability and computational efficiency, for example, by exploring potential enhancements in the Q-matrix updating procedure (Gu & Dunson, 2023).

## Supporting information

10.1017/psy.2026.10093.sm001 Wang et al. supplementary material Wang et al. supplementary material

## Data Availability

Publicly available dataset was analyzed in this study. The dataset can be found as follows: https://cran.r-project.org/web/packages/CDM/index.html.

## Supplementary material

The supplementary material for this article can be found at https://doi.org/10.1017/psy.2026.10093.

## Table 1 Long description

The table is organized with column headers at the top, including Step, Description, and Output. Each row details a sequential action in the M H dash Gibbs sampling process. The first row describes initializing parameters and states. Subsequent rows outline iterative steps such as sampling from conditional distributions, updating variables, and recording outputs. The final row summarizes the completion of the sampling procedure. All technical terms and variable names are presented exactly as in the table.

Navigate back to Table 1.

## Figure 2 Long description

The left panel, titled Sampling procedure, starts with Initialization and input hyperparameters, then Update alpha and pi, followed by Draw a mini-batch, then Update s, g, Q and G. A decision diamond checks if t is less than T; if Yes, the process loops back to Update alpha and pi, if No, it ends. An arrow leads to the center panel, Relabel procedure, which contains Perform relabeling on all posterior samples of Q, G, and alpha. Another arrow leads to the right panel, Estimation procedure. This begins with Compute posterior sample means of s, g, pi, alpha, Q and G. Arrows split: one path sends alpha, Q, G to Compare with a threshold to determine each element as 0 or 1, then Q to Compute structured Q, then Q to Final estimation of s, g, pi, alpha, Q and G. Another path sends s, g, pi directly to Final estimation. The flowchart uses arrows to indicate the sequence and dependencies between steps.

Navigate back to Figure 2.

## Table 2 Long description

From the top row, the left column lists simulation factors and the right column lists their levels. The first factor is sample size and test length, with levels N equals 500 and J equals 20, and N equals 1,000 and J equals 40. The next factor is minibatch size, with levels N sub 1 equals 128 and 256 for N equals 500, and N sub 1 equals 128, 256, and 512 for N equals 1,000. The cutoff value factor has levels 0.2, 0.3, and 0.4. Attribute profile distribution has levels balanced and unbalanced. Attribute hierarchy has levels G sub 1, G sub 2, G sub 3, and G sub 4, as referenced in Figure 1.

Navigate back to Table 2.

## Table 3 Long description

The table contains two main sections: Balanced and Unbalanced. Each section lists parameter combinations for N, J, K, and N sub 1. For Balanced: N values are 500 or 1,000, J is 20 or 40, K is 4, N sub 1 is 128, 256, or 512. For Unbalanced: N is 500 or 1,000, J is 20 or 40, K is 4, N sub 1 is 128, 256, or 512. For each row, computation times are given for G sub 1, G sub 2, G sub 3, and G sub 4. For example, in the Balanced section with N 500, J 20, K 4, N sub 1 128, the times are G sub 1 165.0952, G sub 2 195.3910, G sub 3 223.8264, G sub 4 208.2667. As N and N sub 1 increase, computation times generally rise. The highest times are observed for N 1,000, N sub 1 512, with G sub 3 reaching 1,078.9910 in Balanced and 1,095.6220 in Unbalanced. Unbalanced conditions tend to have slightly lower computation times than Balanced for the same parameter sets.

Navigate back to Table 3.

## Table 4 Long description

The table is divided into two main sections by the overall recovery metric, first for Q, then for G. Each section has columns for N equals 500 and N equals 1,000, each split into balanced and unbalanced. For Q: G sub 1, N sub 1 equals 256 c equals 0.2 or 0.3 (balanced, N equals 500), N sub 1 equals 256 c equals 0.3 (unbalanced, N equals 500), N sub 1 equals 512 c equals 0.2 or 0.3 (balanced and unbalanced, N equals 1,000). G sub 2, N sub 1 equals 256 c equals 0.2 (balanced, N equals 500), N sub 1 equals 256 c equals 0.2 or 0.3 (unbalanced, N equals 500), N sub 1 equals 256 c equals 0.2 or 0.3 or 0.4 (balanced, N equals 1,000), N sub 1 equals 512 c equals 0.2 or 0.3 (unbalanced, N equals 1,000). G sub 3, N sub 1 equals 128 c equals 0.2 (balanced, N equals 500), N sub 1 equals 256 c equals 0.2 (unbalanced, N equals 500), N sub 1 equals 256 c equals 0.2 or 0.3 or 0.4 (balanced, N equals 1,000), N sub 1 equals 256 or 512 c equals 0.2 or 0.3 or 0.4 (unbalanced, N equals 1,000). G sub 4, N sub 1 equals 256 c equals 0.2 or 0.3 (balanced, N equals 500), N sub 1 equals 256 c equals 0.2 (unbalanced, N equals 500), N sub 1 equals 512 c equals 0.2 or 0.3 (balanced, N equals 1,000), N sub 1 equals 256 or 512 c equals 0.2 or 0.3 (unbalanced, N equals 1,000). Summary for Q: N sub 1 equals 128 (1 time) or 256 (7 times) for N equals 500, N sub 1 equals 256 (4 times) or 512 (6 times) for N equals 1,000. c equals 0.2 (7 times) or 0.3 (4 times) for N equals 500, c equals 0.2 (8 times), 0.3 (8 times), 0.4 (3 times) for N equals 1,000. For G: G sub 1, N sub 1 equals 256 c equals 0.3 (balanced and unbalanced, N equals 500), N sub 1 equals 512 c equals 0.3 (balanced, N equals 1,000), N sub 1 equals 512 c equals 0.2 or 0.3 (unbalanced, N equals 1,000). G sub 2, N sub 1 equals 256 c equals 0.2 (balanced and unbalanced, N equals 500), N sub 1 equals 256 c equals 0.2 or 0.3 or 0.4 (balanced, N equals 1,000), N sub 1 equals 512 c equals 0.2 or 0.3 (unbalanced, N equals 1,000). G sub 3, N sub 1 equals 128 or 256 c equals 0.2 (balanced, N equals 500), N sub 1 equals 256 c equals 0.2 (unbalanced, N equals 500), N sub 1 equals 256 c equals 0.2 or 0.3 (balanced, N equals 1,000), N sub 1 equals 256 or 512 c equals 0.2 or 0.3 (unbalanced, N equals 1,000). G sub 4, N sub 1 equals 256 c equals 0.2 or 0.3 (balanced, N equals 500), N sub 1 equals 256 c equals 0.2 (unbalanced, N equals 500), N sub 1 equals 512 c equals 0.2 (balanced, N equals 1,000), N sub 1 equals 256 or 512 c equals 0.2 or 0.3 (unbalanced, N equals 1,000). Summary for G: N sub 1 equals 128 (1 time) or 256 (8 times) for N equals 500, N sub 1 equals 256 (4 times) or 512 (6 times) for N equals 1,000. c equals 0.2 (6 times) or 0.3 (3 times) for N equals 500, c equals 0.2 (7 times), 0.3 (7 times), 0.4 (1 time) for N equals 1,000.

Navigate back to Table 4.

## Table 5 Long description

From top to bottom, the table is split into Balanced and Unbalanced sections. Each section lists G 1, G 2, G 3, and G 4, with two rows per group: N equals 500, J equals 20 and N equals 1,000, J equals 40. Columns, from left to right, are group, sample size, T P R, T F R, R T P R, R T F R, and number of exact G-matrix recoveries. For Balanced: G 1 has T P R 0.900 and 0.927, T F R 0.972 and 0.983, R T P R 0.986 and 0.976, R T F R 0.967 and 0.960, recoveries 44 and 44. G 2: T P R 0.985 and 0.910, T F R 0.997 and 0.978, R T P R 0.996 and 0.976, R T F R 0.997 and 0.969, recoveries 49 and 45. G 3: T P R 0.973 and 0.947, T F R 0.995 and 0.988, R T P R 0.989 and 0.977, R T F R 0.991 and 0.982, recoveries 48 and 46. G 4: all metrics 1.000, recoveries 50 for both sample sizes. For Unbalanced: G 1 has T P R 0.853 and 0.987, T F R 0.963 and 0.997, R T P R 0.978 and 0.996, R T F R 0.957 and 0.993, recoveries 40 and 49. G 2: T P R 0.920 and 0.980, T F R 0.980 and 0.995, R T P R 0.982 and 0.996, R T F R 0.977 and 0.994, recoveries 46 and 49. G 3: all metrics 1.000, recoveries 50 for both sample sizes. G 4: T P R 0.960 and 1.000, T F R 0.994 and 1.000, R T P R 0.990 and 1.000, R T F R 0.993 and 1.000, recoveries 46 and 50. T P R and T F R are true positive and true negative rates based on the G-matrix; R T P R and R T F R are rates based on the reachability matrix. The last column is the count of exact G-matrix recoveries.

Navigate back to Table 5.

## Table 6 Long description

The table consists of four main row groups labeled G sub 1, G sub 2, G sub 3, and G sub 4, each followed by three unlabeled rows. Columns are divided into Balanced and Unbalanced groups, each with four columns of values. For G sub 1, Balanced values are 0.000, bold 0.469, 0.376, 0.304; Unbalanced values are 0.000, bold 0.471, 0.377, 0.298. The next three rows under G sub 1 show Balanced: 0.000, 0.000, bold 0.473, 0.343; 0.000, 0.000, 0.000, bold 0.436; 0.031, 0.040, 0.040, 0.000. Unbalanced: 0.000, 0.000, bold 0.473, 0.372; 0.000, 0.000, 0.000, bold 0.476; 0.007, 0.008, 0.007, 0.000. For G sub 2, Balanced: 0.000, bold 0.462, bold 0.465, 0.300; 0.000, 0.000, 0.000, bold 0.431; 0.000, 0.000, 0.000, bold 0.420; 0.030, 0.042, 0.040, 0.000. Unbalanced: 0.000, bold 0.454, bold 0.454, 0.299; 0.000, 0.000, 0.000, bold 0.472; 0.000, 0.000, 0.000, bold 0.469; 0.008, 0.008, 0.007, 0.000. For G sub 3, Balanced: 0.000, bold 0.466, bold 0.432, bold 0.446; 0.001, 0.000, 0.001, 0.011; 0.000, 0.026, 0.000, 0.010; 0.000, 0.023, 0.000, 0.000. Unbalanced: 0.000, bold 0.470, bold 0.473, bold 0.479; 0.000, 0.000, 0.000, 0.000; 0.000, 0.000, 0.000, 0.000; 0.000, 0.000, 0.000, 0.000. For G sub 4, Balanced: 0.000, bold 0.465, bold 0.470, 0.372; 0.000, 0.000, 0.000, 0.000; 0.000, 0.000, 0.000, bold 0.470; 0.000, 0.000, 0.000, 0.000. Unbalanced: 0.000, bold 0.465, bold 0.467, 0.372; 0.000, 0.000, 0.000, 0.000; 0.000, 0.000, 0.000, bold 0.473; 0.000, 0.000, 0.000, 0.000. Bolded values in each group represent the real edges as noted in the table footnote.

Navigate back to Table 6.

## Table 7 Long description

The table is divided into Balanced and Unbalanced sections. Each section lists four groups, G sub 1 to G sub 4, with two sample sizes per group: N equals 500, J equals 20 and N equals 1,000, J equals 40. For each group and sample size, columns report QRR1, QRR2, QRR3, QRR4, AQRR, and the number of times the entire Q-matrix was recovered correctly. In the Balanced section: G sub 1, N equals 500, J equals 20 has QRR1 to QRR3 at 1.000, QRR4 at 0.930, AQRR at 0.983, and 44 correct recoveries; N equals 1,000, J equals 40 has QRR1 to QRR3 at 1.000, QRR4 at 0.903, AQRR at 0.976, and 44 correct recoveries. G sub 2, N equals 500, J equals 20 has QRR1 to QRR3 at 1.000, QRR4 at 0.984, AQRR at 0.996, and 48 correct recoveries; N equals 1,000, J equals 40 has QRR1 to QRR3 at 1.000, QRR4 at 0.918, AQRR at 0.980, and 45 correct recoveries. G sub 3, N equals 500, J equals 20 has QRR1 at 0.984, QRR2 at 0.993, QRR3 at 1.000, QRR4 at 0.991, AQRR at 0.992, and 46 correct recoveries; N equals 1,000, J equals 40 has QRR1 at 0.969, QRR2 at 0.979, QRR3 at 0.998, QRR4 at 0.992, AQRR at 0.985, and 46 correct recoveries. G sub 4, N equals 500, J equals 20 has QRR1 to QRR3 at 1.000, QRR4 at 0.991, AQRR at 0.998, and 49 correct recoveries; N equals 1,000, J equals 40 has all QRRs at 1.000, AQRR at 1.000, and 50 correct recoveries. In the Unbalanced section: G sub 1, N equals 500, J equals 20 has QRR1 at 0.996, QRR2 at 0.951, QRR3 at 1.000, QRR4 at 0.953, AQRR at 0.975, and 30 correct recoveries; N equals 1,000, J equals 40 has all QRRs at 1.000 except QRR4 at 0.984, AQRR at 0.996, and 49 correct recoveries. G sub 2, N equals 500, J equals 20 has QRR1 at 0.991, QRR2 at 0.997, QRR3 at 0.996, QRR4 at 0.950, AQRR at 0.984, and 44 correct recoveries; N equals 1,000, J equals 40 has all QRRs at 1.000 except QRR4 at 0.984, AQRR at 0.996, and 49 correct recoveries. G sub 3, N equals 500, J equals 20 and N equals 1,000, J equals 40 both have all QRRs at 1.000, AQRR at 1.000, and 50 correct recoveries. G sub 4, N equals 500, J equals 20 has QRR1 at 0.989, QRR2 at 0.999, QRR3 at 0.982, QRR4 at 0.993, AQRR at 0.991, and 45 correct recoveries; N equals 1,000, J equals 40 has all QRRs at 1.000, AQRR at 1.000, and 50 correct recoveries. QRR1 to QRR4 are correct recovery rates for four attributes, AQRR is the element-wise recovery rate, and the last column is the number of times the entire Q-matrix was recovered correctly.

Navigate back to Table 7.

## Table 8 Long description

The table is divided into Balanced and Unbalanced sections. Each section lists four groups, G 1 to G 4, with two sample size conditions per group: N equals 500, J equals 20 and N equals 1,000, J equals 40. Columns, from left to right, are group, sample size, ARMSE sub s open parenthesis ABias sub s close parenthesis, 95 percent AHPDI sub s, ARMSE sub g open parenthesis ABias sub g close parenthesis, 95 percent AHPDI sub g, and ARMSE sub pi open parenthesis ABias sub pi close parenthesis. For Balanced: G 1, N equals 500, J equals 20, ARMSE sub s is 0.038 open parenthesis 0.009 close parenthesis, 95 percent AHPDI sub s is open bracket 0.116, 0.305 close bracket, ARMSE sub g is 0.033 open parenthesis 0.007 close parenthesis, 95 percent AHPDI sub g is open bracket 0.110, 0.308 close bracket, ARMSE sub pi is 0.023 open parenthesis 0.000 close parenthesis. G 1, N equals 1,000, J equals 40, ARMSE sub s is 0.032 open parenthesis 0.008 close parenthesis, 95 percent AHPDI sub s is open bracket 0.143, 0.274 close bracket, ARMSE sub g is 0.020 open parenthesis 0.003 close parenthesis, 95 percent AHPDI sub g is open bracket 0.137, 0.270 close bracket, ARMSE sub pi is 0.026 open parenthesis 0.000 close parenthesis. G 2, N equals 500, J equals 20, ARMSE sub s is 0.037 open parenthesis 0.012 close parenthesis, 95 percent AHPDI sub s is open bracket 0.112, 0.316 close bracket, ARMSE sub g is 0.031 open parenthesis 0.007 close parenthesis, 95 percent AHPDI sub g is open bracket 0.109, 0.309 close bracket, ARMSE sub pi is 0.018 open parenthesis 0.000 close parenthesis. G 2, N equals 1,000, J equals 40, ARMSE sub s is 0.033 open parenthesis 0.011 close parenthesis, 95 percent AHPDI sub s is open bracket 0.140, 0.287 close bracket, ARMSE sub g is 0.021 open parenthesis 0.004 close parenthesis, 95 percent AHPDI sub g is open bracket 0.138, 0.274 close bracket, ARMSE sub pi is 0.028 open parenthesis 0.000 close parenthesis. G 3, N equals 500, J equals 20, ARMSE sub s is 0.036 open parenthesis 0.008 close parenthesis, 95 percent AHPDI sub s is open bracket 0.099, 0.323 close bracket, ARMSE sub g is 0.058 open parenthesis 0.024 close parenthesis, 95 percent AHPDI sub g is open bracket 0.116, 0.337 close bracket, ARMSE sub pi is 0.022 open parenthesis 0.000 close parenthesis. G 3, N equals 1,000, J equals 40, ARMSE sub s is 0.029 open parenthesis 0.011 close parenthesis, 95 percent AHPDI sub s is open bracket 0.135, 0.288 close bracket, ARMSE sub g is 0.040 open parenthesis 0.013 close parenthesis, 95 percent AHPDI sub g is open bracket 0.137, 0.274 close bracket, ARMSE sub pi is 0.020 open parenthesis 0.000 close parenthesis. G 4, N equals 500, J equals 20, ARMSE sub s is 0.037 open parenthesis 0.010 close parenthesis, 95 percent AHPDI sub s is open bracket 0.109, 0.315 close bracket, ARMSE sub g is 0.037 open parenthesis 0.010 close parenthesis, 95 percent AHPDI sub g is open bracket 0.109, 0.315 close bracket, ARMSE sub pi is 0.014 open parenthesis 0.000 close parenthesis. G 4, N equals 1,000, J equals 40, ARMSE sub s is 0.022 open parenthesis 0.006 close parenthesis, 95 percent AHPDI sub s is open bracket 0.133, 0.303 close bracket, ARMSE sub g is 0.022 open parenthesis 0.004 close parenthesis, 95 percent AHPDI sub g is open bracket 0.143, 0.289 close bracket, ARMSE sub pi is 0.006 open parenthesis 0.000 close parenthesis. For Unbalanced: G 1, N equals 500, J equals 20, ARMSE sub s is 0.025 open parenthesis 0.003 close parenthesis, 95 percent AHPDI sub s is open bracket 0.130, 0.278 close bracket, ARMSE sub g is 0.038 open parenthesis 0.010 close parenthesis, 95 percent AHPDI sub g is open bracket 0.095, 0.330 close bracket, ARMSE sub pi is 0.013 open parenthesis 0.000 close parenthesis. G 1, N equals 1,000, J equals 40, ARMSE sub s is 0.016 open parenthesis 0.002 close parenthesis, 95 percent AHPDI sub s is open bracket 0.151, 0.255 close bracket, ARMSE sub g is 0.024 open parenthesis 0.003 close parenthesis, 95 percent AHPDI sub g is open bracket 0.127, 0.281 close bracket, ARMSE sub pi is 0.007 open parenthesis 0.000 close parenthesis. G 2, N equals 500, J equals 20, ARMSE sub s is 0.023 open parenthesis 0.004 close parenthesis, 95 percent AHPDI sub s is open bracket 0.132, 0.279 close bracket, ARMSE sub g is 0.065 open parenthesis 0.025 close parenthesis, 95 percent AHPDI sub g is open bracket 0.086, 0.375 close bracket, ARMSE sub pi is 0.017 open parenthesis 0.000 close parenthesis. G 2, N equals 1,000, J equals 40, ARMSE sub s is 0.017 open parenthesis 0.002 close parenthesis, 95 percent AHPDI sub s is open bracket 0.150, 0.255 close bracket, ARMSE sub g is 0.022 open parenthesis 0.002 close parenthesis, 95 percent AHPDI sub g is open bracket 0.130, 0.277 close bracket, ARMSE sub pi is 0.007 open parenthesis 0.000 close parenthesis. G 3, N equals 500, J equals 20, ARMSE sub s is 0.030 open parenthesis 0.007 close parenthesis, 95 percent AHPDI sub s is open bracket 0.116, 0.302 close bracket, ARMSE sub g is 0.030 open parenthesis 0.005 close parenthesis, 95 percent AHPDI sub g is open bracket 0.112, 0.302 close bracket, ARMSE sub pi is 0.011 open parenthesis 0.000 close parenthesis. G 3, N equals 1,000, J equals 40, ARMSE sub s is 0.019 open parenthesis 0.003 close parenthesis, 95 percent AHPDI sub s is open bracket 0.141, 0.266 close bracket, ARMSE sub g is 0.019 open parenthesis 0.001 close parenthesis, 95 percent AHPDI sub g is open bracket 0.139, 0.265 close bracket, ARMSE sub pi is 0.004 open parenthesis 0.000 close parenthesis. G 4, N equals 500, J equals 20, ARMSE sub s is 0.028 open parenthesis 0.006 close parenthesis, 95 percent AHPDI sub s is open bracket 0.122, 0.294 close bracket, ARMSE sub g is 0.078 open parenthesis 0.031 close parenthesis, 95 percent AHPDI sub g is open bracket 0.103, 0.367 close bracket, ARMSE sub pi is 0.015 open parenthesis 0.000 close parenthesis. G 4, N equals 1,000, J equals 40, ARMSE sub s is 0.018 open parenthesis 0.003 close parenthesis, 95 percent AHPDI sub s is open bracket 0.146, 0.261 close bracket, ARMSE sub g is 0.020 open parenthesis 0.002 close parenthesis, 95 percent AHPDI sub g is open bracket 0.134, 0.271 close bracket, ARMSE sub pi is 0.003 open parenthesis 0.000 close parenthesis. ARMSE, ABias, and AHPDI are defined as average root mean squared error, average bias, and average high density posterior interval across all items or latent classes.

Navigate back to Table 8.

## Table 9 Long description

The table has rows for four groups labeled G sub 1 to G sub 4, each with two sample sizes N equals 500, J equals 20 and N equals 1,000, J equals 40. Columns are divided into balanced and unbalanced scenarios. For balanced, columns are AAR1, AAR2, AAR3, AAR4, and PAR. For unbalanced, columns are AAR1, AAR2, AAR3, AAR4, and PAR. For G sub 1, N equals 500, J equals 20, balanced values are 0.968, 0.959, 0.964, 0.929, 0.839; unbalanced are 0.976, 0.981, 0.978, 0.949, 0.897. For G sub 1, N equals 1,000, J equals 40, balanced are 0.990, 0.989, 0.989, 0.927, 0.899; unbalanced are 0.995, 0.996, 0.995, 0.983, 0.970. For G sub 2, N equals 500, J equals 20, balanced are 0.961, 0.948, 0.947, 0.962, 0.837; unbalanced are 0.978, 0.965, 0.968, 0.932, 0.863. For G sub 2, N equals 1,000, J equals 40, balanced are 0.985, 0.984, 0.983, 0.925, 0.8820; unbalanced are 0.994, 0.993, 0.994, 0.983, 0.966. For G sub 3, N equals 500, J equals 20, balanced are 0.933, 0.930, 0.920, 0.917, 0.749; unbalanced are 0.962, 0.939, 0.946, 0.935, 0.824. For G sub 3, N equals 1,000, J equals 40, balanced are 0.952, 0.955, 0.971, 0.966, 0.858; unbalanced are 0.988, 0.981, 0.984, 0.978, 0.938. For G sub 4, N equals 500, J equals 20, balanced are 0.963, 0.934, 0.937, 0.946, 0.811; unbalanced are 0.967, 0.939, 0.950, 0.937, 0.826. For G sub 4, N equals 1,000, J equals 40, balanced are 0.988, 0.978, 0.981, 0.983, 0.934; unbalanced are 0.993, 0.988, 0.990, 0.988, 0.963. AAR1 to AAR4 represent correct classification rates for each attribute, PAR is the pattern-wise agreement rate. Higher values are generally observed for larger sample sizes and unbalanced scenarios.

Navigate back to Table 9.

## Table 10 Long description

The table is divided into three column groups by mini-batch size: N sub 1 equals 512, N sub 1 equals 1,024, and N sub 1 equals 1,500. Each group contains three columns of estimated adjacency matrix values for G hat, with bold values marking statistically significant edges. For N sub 1 equals 512, the first row is 0.000, 0.053, 0.052; the second row is bold 0.408, 0.000, 0.170; the third row is bold 0.374, bold 0.364, 0.000. For N sub 1 equals 1,024, the first row is 0.000, 0.029, 0.006; the second row is bold 0.212, 0.000, 0.017; the third row is bold 0.340, bold 0.362, 0.000. For N sub 1 equals 1,500, the first row is 0.000, 0.065, 0.000; the second row is bold 0.426, 0.000, 0.019; the third row is bold 0.407, bold 0.489, 0.000. The D I C values for each group are 83,271.820 for N sub 1 equals 512, 82,280.660 for N sub 1 equals 1,024, and 82,837.410 for N sub 1 equals 1,500. Bold values represent edges with strong statistical evidence.

Navigate back to Table 10.

## Table 11 Long description

The table consists of 28 rows, each corresponding to an item numbered 1 to 28, and six main data columns grouped into two sets of three. The first set, labeled expert labeled structured Q-matrix, contains three columns of binary values for each item. The second set, labeled estimated structured Q-matrix, also contains three columns of binary values. For each item, the values in the expert labeled and estimated matrices are compared side by side. Highlighted cells indicate where the value in the expert labeled matrix differs from the estimated matrix. For example, in item 2, the first expert labeled value is 0 while the corresponding estimated value is also 0, but the second and third values are both 1 in both matrices. In item 4, the third value is 1 in both matrices, but the first and second values are 0 in both. Items such as 9, 14, 17, and 21 contain highlighted cells, marking discrepancies between the expert labeled and estimated matrices. The note below the table clarifies that highlighted elements represent differences between the true structured Q-matrix and the estimated structured Q-matrix.

Navigate back to Table 11.
