# Supplementary material for: Uncovering Latent Structures: A Bayesian Approach to Estimating Q-Matrix and Attribute Hierarchies in Cognitive Diagnostic Models
Source: Psychometrika. 2026 Feb 20;91(2):669–94. doi: 10.1017/psy.2026.10093 (PMC13294626; doi:10.1017/psy.2026.10093)
Supplement: Wang et al. supplementary material [file S0033312326100933sup001.zip › Appendix/Appendix.pdf]

APPENDIX FOR UNCOVERING LATENT STRUCTURES:A BAYESIAN APPROACH TO ESTIMATING Q-MATRIX AND ATTRIBUTE HIERARCHIES IN COGNITIVE DIAGNOSTIC MODELS

1. Appendix A: Convergency analysis in simulation studies

We use the simulation condition of $N = 500$, $J = 20$, $N_1 = 256$ as an example to demonstrate the convergence of our proposed MH within Gibss algorithm in the considered simulation conditions. Figures 1 and 2 present the convergence curves of $\hat{R}$ for the parameters $\boldsymbol{s}$ and $\boldsymbol{g}$ under the 4 hierarchical structures. The horizontal line represents $\hat{R} = 1.1$, and it is evident that under all simulation conditions, both $\boldsymbol{s}$ and $\boldsymbol{g}$ have reached convergence. Note that we cannot compute the $\hat{R}$ for $\boldsymbol{\pi}$, Q, and G, as their distributions or structures change dynamically during the iterations along with the changing attribute hierarchy.

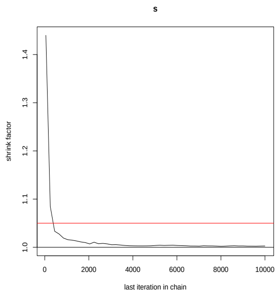

(a) G_1, Balanced

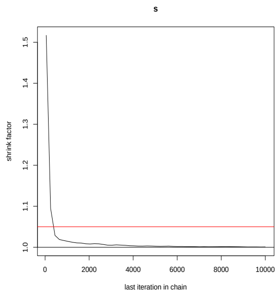

(b) G_2, Balanced

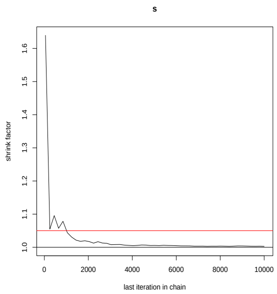

(c) G_3, Balanced

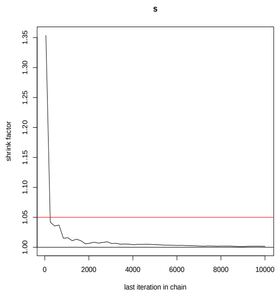

(d) G_4, Balanced

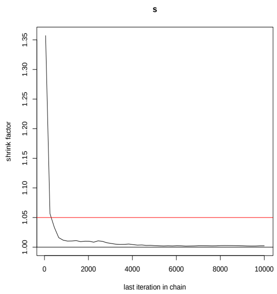

(e) G_1, Unbalanced

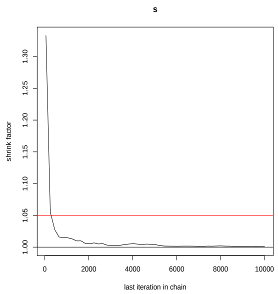

(f) G_2, Unbalanced

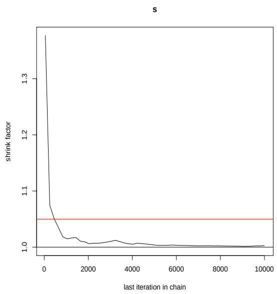

(g) G_3, Unbalanced

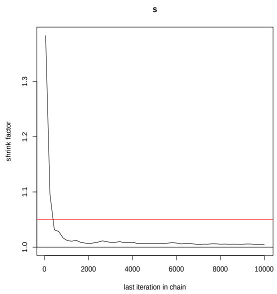

(h) G_4, Unbalanced

Figure 1: $\hat{R}$ for the slipping parameter $\boldsymbol{s}$ under $N = 500$, $J = 20$ condition.

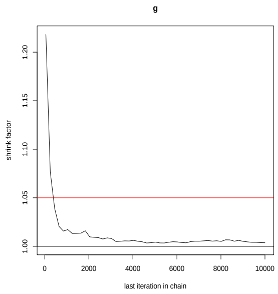

(a) G_1, Balanced

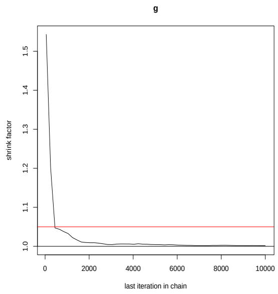

(b) G_2, Balanced

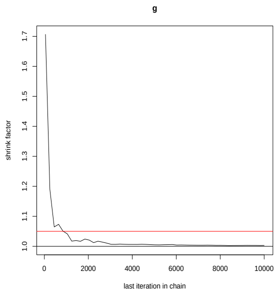

(c) G_3, Balanced

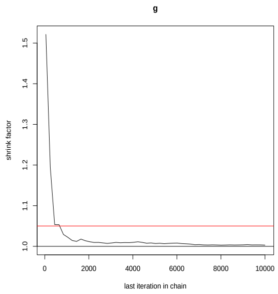

(d) G_4, Balanced

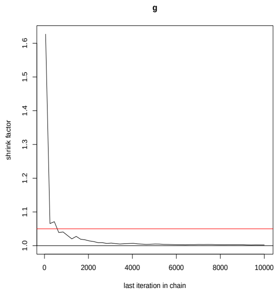

(e) G_1, Unbalanced

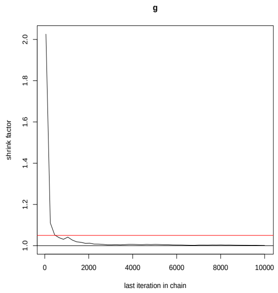

(f) G_2, Unbalanced

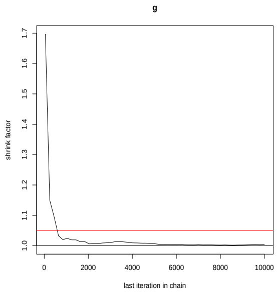

(g) G_3, Unbalanced

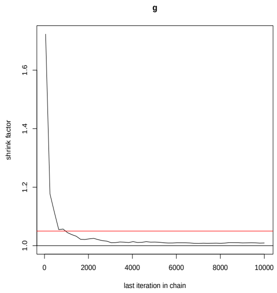

(h) G_4, Unbalanced

Figure 2: $\hat{R}$ for the guessing parameter $\boldsymbol{g}$ under $N = 500$, $J = 20$ condition.

2. Appendix B

Table 1: Evaluate the recovery accuracy of **Q** and **G** under different sample size and cut value.

	N1	cut value	QRR1	QRR2	QRR3	QRR4	QARR	$\hat{Q} = Q$	TPR	TFR	RTPR	RTFR	$\hat{G} = G$
**Balanced/G1**													
$N = 500$	128	0.2	1.000	0.981	0.968	0.867	0.954	37	0.800	0.952	0.960	0.930	38
	128	0.3	1.000	0.987	0.963	0.857	0.952	37	0.873	0.969	0.944	0.917	38
	128	0.4	0.984	0.990	0.969	0.891	0.959	37	0.840	0.983	0.910	0.980	36
	256	0.2	1.000	1.000	1.000	0.930	0.983	44	0.900	0.972	0.986	0.967	44
	256	0.3	1.000	1.000	1.000	0.939	0.985	44	0.947	0.986	0.982	0.977	45
	256	0.4	0.985	0.992	0.998	0.950	0.981	40	0.907	0.991	0.940	0.997	40
$N = 1000$	128	0.2	1.000	1.000	0.988	0.797	0.946	35	0.780	0.946	0.952	0.913	35
	128	0.3	1.000	1.000	0.988	0.797	0.946	35	0.780	0.946	0.952	0.913	35
	128	0.4	0.994	1.000	0.988	0.800	0.945	35	0.887	0.972	0.924	0.930	35
	256	0.2	1.000	0.995	1.000	0.801	0.949	35	0.767	0.940	0.954	0.910	35
	256	0.3	1.000	0.995	1.000	0.803	0.950	36	0.813	0.952	0.942	0.897	35
	256	0.4	0.999	0.996	1.000	0.812	0.952	36	0.887	0.977	0.922	0.957	35
	512	0.2	1.000	1.000	1.000	0.903	0.976	44	0.927	0.983	0.976	0.960	44
	512	0.3	1.000	1.000	1.000	0.902	0.976	44	0.947	0.988	0.976	0.960	45
	512	0.4	0.991	0.997	1.000	0.911	0.975	41	0.927	0.989	0.954	0.977	41
**Unbalanced/G1**													
	N1	cut value	QRR1	QRR2	QRR3	QRR4	QARR	$\hat{Q} = Q$	TPR	TFR	RTPR	RTFR	$\hat{G} = G$
$N = 500$	128	0.2	0.985	0.811	0.836	0.880	0.878	4	0.313	0.835	0.870	0.770	5
	128	0.3	0.987	0.927	0.934	0.928	0.944	8	0.660	0.940	0.850	0.967	20
	128	0.4	0.842	0.857	0.918	0.935	0.888	1	0.240	0.985	0.510	1.000	0
	256	0.2	0.996	0.951	1.000	0.953	0.975	30	0.853	0.963	0.978	0.957	40
	256	0.3	0.995	0.979	1.000	0.958	0.983	33	0.920	0.982	0.968	0.977	42
	256	0.4	0.950	0.957	1.000	0.969	0.969	26	0.800	0.988	0.866	1.000	28
$N = 1000$	128	0.2	1.000	0.929	0.950	0.887	0.941	23	0.660	0.917	0.940	0.890	27
	128	0.3	0.998	0.963	0.973	0.889	0.956	24	0.873	0.966	0.944	0.953	37
	128	0.4	0.930	0.942	0.964	0.909	0.936	22	0.720	0.995	0.810	1.000	27
	256	0.2	1.000	0.983	0.998	0.924	0.976	43	0.900	0.977	0.976	0.960	43
	256	0.3	1.000	0.983	0.998	0.922	0.976	43	0.913	0.980	0.972	0.953	43
	256	0.4	0.991	0.979	0.998	0.931	0.975	43	0.920	0.989	0.956	0.983	43
	512	0.2	1.000	1.000	1.000	0.984	0.996	49	0.987	0.997	0.996	0.993	49
	512	0.3	1.000	1.000	1.000	0.982	0.996	49	0.993	0.998	0.994	0.990	49
	512	0.4	0.998	0.996	1.000	0.987	0.995	47	0.980	0.998	0.984	1.000	47

Table 2: Evaluate the recovery accuracy of **Q** and **G** under different sample size and cut value.

	N1	cut value	QRR1	QRR2	QRR3	QRR4	QARR	$\hat{Q}=Q$	TPR	TFR	RTPR	RTFR	$\hat{G}=G$
**Balanced/G2**													
$N=500$	128	0.2	1.000	0.999	0.984	0.880	0.966	40	0.830	0.960	0.967	0.951	40
	128	0.3	0.997	0.999	0.988	0.878	0.966	39	0.845	0.968	0.958	0.949	38
	128	0.4	0.967	0.998	0.990	0.884	0.960	35	0.815	0.978	0.893	0.963	31
	256	0.2	1.000	1.000	1.000	0.984	0.996	48	0.985	0.997	0.996	0.997	49
	256	0.3	1.000	1.000	1.000	0.984	0.996	48	0.980	0.997	0.993	0.997	48
	256	0.4	0.987	0.993	1.000	0.985	0.991	42	0.935	0.998	0.964	1.000	41
$N=1000$	128	0.2	1.000	1.000	1.000	0.850	0.963	39	0.800	0.952	0.951	0.949	39
	128	0.3	1.000	1.000	1.000	0.850	0.963	39	0.810	0.955	0.953	0.951	39
	128	0.4	0.999	1.000	1.000	0.852	0.963	39	0.880	0.972	0.929	0.960	38
	256	0.2	1.000	1.000	1.000	0.945	0.986	46	0.930	0.983	0.982	0.983	46
	256	0.3	1.000	1.000	1.000	0.945	0.986	46	0.940	0.987	0.980	0.980	46
	256	0.4	0.999	1.000	1.000	0.948	0.987	46	0.955	0.995	0.971	0.991	46
	512	0.2	1.000	1.000	1.000	0.918	0.980	45	0.910	0.978	0.976	0.969	45
	512	0.3	1.000	1.000	1.000	0.915	0.979	45	0.930	0.985	0.971	0.963	45
	512	0.4	0.995	1.000	0.998	0.920	0.978	42	0.910	0.988	0.953	0.983	42
**Unbalanced/G2**													
	N1	cut value	QRR1	QRR2	QRR3	QRR4	QARR	$\hat{Q}=Q$	TPR	TFR	RTPR	RTFR	$\hat{G}=G$
$N=500$	128	0.2	0.989	0.956	0.975	0.785	0.926	25	0.675	0.917	0.938	0.874	30
	128	0.3	0.965	0.968	0.983	0.842	0.940	25	0.695	0.947	0.876	0.926	26
	128	0.4	0.785	0.976	0.981	0.915	0.914	11	0.375	1.000	0.647	1.000	10
	256	0.2	0.991	0.997	0.996	0.950	0.984	44	0.920	0.980	0.982	0.977	46
	256	0.3	0.979	0.999	0.998	0.955	0.983	42	0.920	0.983	0.976	0.986	44
	256	0.4	0.963	0.995	0.997	0.971	0.982	37	0.905	0.998	0.947	0.997	39
$N=1000$	128	0.2	1.000	0.989	0.989	0.789	0.942	34	0.745	0.938	0.944	0.923	36
	128	0.3	1.000	0.989	0.999	0.789	0.944	35	0.770	0.947	0.942	0.929	36
	128	0.4	0.997	0.990	0.999	0.792	0.944	35	0.825	0.967	0.893	0.943	33
	256	0.2	1.000	1.000	1.000	0.884	0.971	42	0.865	0.967	0.969	0.963	43
	256	0.3	1.000	1.000	1.000	0.883	0.971	42	0.885	0.973	0.964	0.957	43
	256	0.4	0.997	1.000	1.000	0.888	0.971	42	0.905	0.982	0.944	0.971	43
	512	0.2	1.000	1.000	1.000	0.984	0.996	49	0.980	0.995	0.996	0.994	49
	512	0.3	1.000	1.000	1.000	0.983	0.996	49	0.990	0.998	0.993	0.991	49
	512	0.4	0.994	0.995	1.000	0.988	0.994	43	0.955	1.000	0.978	1.000	43

Table 3: Evaluate the recovery accuracy of $\mathbf{Q}$ and $\mathbf{G}$ under different sample size and cut value.

**Balanced/G3**													
	N1	cut value	QRR1	QRR2	QRR3	QRR4	QARR	$\hat{Q}=Q$	TPR	TFR	RTPR	RTFR	$\hat{G}=G$
$N=500$	128	0.2	0.991	0.997	0.980	1.000	0.992	47	0.967	0.994	0.991	0.989	48
	128	0.3	0.990	0.999	0.983	1.000	0.993	46	0.953	0.997	0.986	0.996	47
	128	0.4	0.943	0.999	0.983	1.000	0.981	42	0.873	0.997	0.946	0.996	42
	256	0.2	0.984	0.993	1.000	0.991	0.992	46	0.973	0.995	0.989	0.991	48
	256	0.3	0.956	1.000	1.000	0.991	0.987	43	0.920	0.997	0.966	0.996	44
	256	0.4	0.906	1.000	1.000	0.991	0.974	39	0.840	0.997	0.931	0.996	40
$N=1000$	128	0.2	1.000	1.000	0.984	0.985	0.992	48	0.973	0.994	1.000	0.991	48
	128	0.3	1.000	1.000	0.984	0.985	0.992	48	0.973	0.994	1.000	0.991	48
	128	0.4	1.000	1.000	0.984	0.985	0.992	48	0.987	0.994	0.994	0.991	48
	256	0.2	1.000	1.000	0.983	1.000	0.996	49	0.987	0.997	1.000	0.996	49
	256	0.3	1.000	1.000	0.983	1.000	0.996	49	0.987	0.997	1.000	0.996	49
	256	0.4	1.000	1.000	0.983	1.000	0.996	49	0.993	0.997	0.997	0.996	49
	512	0.2	0.969	0.979	0.998	0.992	0.985	46	0.947	0.988	0.977	0.982	46
	512	0.3	0.969	0.979	0.998	0.992	0.985	46	0.947	0.991	0.977	0.987	46
	512	0.4	0.960	0.979	0.998	0.992	0.982	41	0.907	0.991	0.960	0.987	41
**Unbalanced/G3**													
	N1	cut value	QRR1	QRR2	QRR3	QRR4	QARR	TPR	TFR	RTPR	RTFR		
$N=500$	128	0.2	1.000	0.956	0.986	0.971	0.978	42	0.927	0.983	0.994	0.967	42
	128	0.3	0.998	0.963	0.997	0.979	0.984	44	0.960	0.994	0.986	0.978	45
	128	0.4	0.989	0.967	0.997	0.985	0.984	43	0.913	0.994	0.963	0.991	42
	256	0.2	1.000	1.000	1.000	1.000	1.000	50	1.000	1.000	1.000	1.000	50
	256	0.3	0.998	1.000	1.000	1.000	1.000	49	0.993	1.000	0.997	1.000	49
	256	0.4	0.994	1.000	1.000	1.000	0.998	45	0.967	1.000	0.986	1.000	45
$N=1000$	128	0.2	1.000	0.984	1.000	0.984	0.992	48	0.980	0.995	0.997	0.989	48
	128	0.3	1.000	0.984	1.000	0.984	0.992	48	0.980	0.995	0.997	0.989	48
	128	0.4	1.000	0.985	1.000	0.984	0.992	48	0.987	0.994	0.994	0.991	48
	256	0.2	1.000	1.000	1.000	1.000	1.000	50	1.000	1.000	1.000	1.000	50
	256	0.3	1.000	1.000	1.000	1.000	1.000	50	1.000	1.000	1.000	1.000	50
	256	0.4	1.000	1.000	1.000	1.000	1.000	50	1.000	1.000	1.000	1.000	50
	512	0.2	1.000	1.000	1.000	1.000	1.000	50	1.000	1.000	1.000	1.000	50
	512	0.3	1.000	1.000	1.000	1.000	1.000	50	1.000	1.000	1.000	1.000	50
	512	0.4	0.999	1.000	1.000	1.000	1.000	48	0.987	1.000	0.994	1.000	48

Table 4: Evaluate the recovery accuracy of **Q** and **G** under different sample size and cut value.

	N1	cut value	QRR1	QRR2	QRR3	QRR4	QARR	$\hat{Q}=Q$	TPR	TFR	RTPR	RTFR	$\hat{G}=G$
**Balanced/G4**													
$N=500$	128	0.2	1.000	0.999	0.979	0.958	0.984	43	0.953	0.986	0.998	0.980	43
	128	0.3	1.000	1.000	0.979	0.958	0.984	43	0.947	0.988	0.995	0.985	44
	128	0.4	0.988	1.000	0.983	0.958	0.982	44	0.913	0.991	0.955	0.990	42
	256	0.2	1.000	1.000	1.000	0.991	0.998	49	1.000	1.000	1.000	1.000	50
	256	0.3	1.000	1.000	1.000	0.991	0.998	49	1.000	1.000	1.000	1.000	50
	256	0.4	0.980	1.000	0.998	0.991	0.992	42	0.940	0.994	0.970	1.000	42
$N=1000$	128	0.2	1.000	1.000	1.000	0.903	0.976	44	0.880	0.972	0.985	0.970	44
	128	0.3	1.000	1.000	1.000	0.903	0.976	44	0.880	0.972	0.985	0.970	44
	128	0.4	0.998	1.000	1.000	0.903	0.976	44	0.960	0.982	0.970	0.970	44
	256	0.2	1.000	0.985	1.000	0.982	0.992	48	0.973	0.994	0.995	0.988	48
	256	0.3	1.000	0.985	1.000	0.982	0.992	48	0.973	0.994	0.995	0.988	48
	256	0.4	1.000	0.986	1.000	0.982	0.992	48	0.980	0.994	0.990	0.990	47
	512	0.2	1.000	1.000	1.000	1.000	1.000	50	1.000	1.000	1.000	1.000	50
	512	0.3	1.000	1.000	1.000	1.000	1.000	49	0.993	1.000	0.998	1.000	49
	512	0.4	0.993	1.000	1.000	1.000	0.998	45	0.967	1.000	0.985	1.000	45
**Unbalanced/G4**													
	N1	cut value	QRR1	QRR2	QRR3	QRR4	QARR	$\hat{Q}=Q$	TPR	TFR	RTPR	RTFR	$\hat{G}=G$
$N=500$	128	0.2	0.984	0.991	0.956	0.947	0.970	37	0.853	0.968	0.975	0.958	39
	128	0.3	0.962	0.997	0.969	0.948	0.969	35	0.860	0.983	0.958	0.983	37
	128	0.4	0.904	0.997	0.969	0.953	0.956	28	0.760	0.986	0.877	0.985	29
	256	0.2	0.989	0.999	0.982	0.993	0.991	45	0.960	0.994	0.990	0.993	46
	256	0.3	0.986	0.999	0.983	0.993	0.990	42	0.953	0.995	0.983	0.998	43
	256	0.4	0.960	0.999	0.980	0.993	0.983	38	0.900	0.991	0.958	0.998	38
$N=1000$	128	0.2	1.000	1.000	0.986	0.942	0.982	44	0.927	0.982	0.993	0.978	44
	128	0.3	1.000	1.000	0.986	0.942	0.982	44	0.920	0.982	0.990	0.978	44
	128	0.4	1.000	1.000	0.988	0.942	0.982	45	0.967	0.988	0.978	0.983	45
	256	0.2	1.000	1.000	1.000	1.000	1.000	50	1.000	1.000	1.000	1.000	50
	256	0.3	1.000	1.000	1.000	1.000	1.000	50	1.000	1.000	1.000	1.000	50
	256	0.4	1.000	1.000	1.000	1.000	1.000	50	1.000	1.000	1.000	1.000	50
	512	0.2	1.000	1.000	1.000	1.000	1.000	50	1.000	1.000	1.000	1.000	50
	512	0.3	1.000	1.000	1.000	1.000	1.000	50	1.000	1.000	1.000	1.000	50
	512	0.4	0.997	1.000	1.000	1.000	0.999	48	0.987	1.000	0.993	1.000	48

3. Appendix C

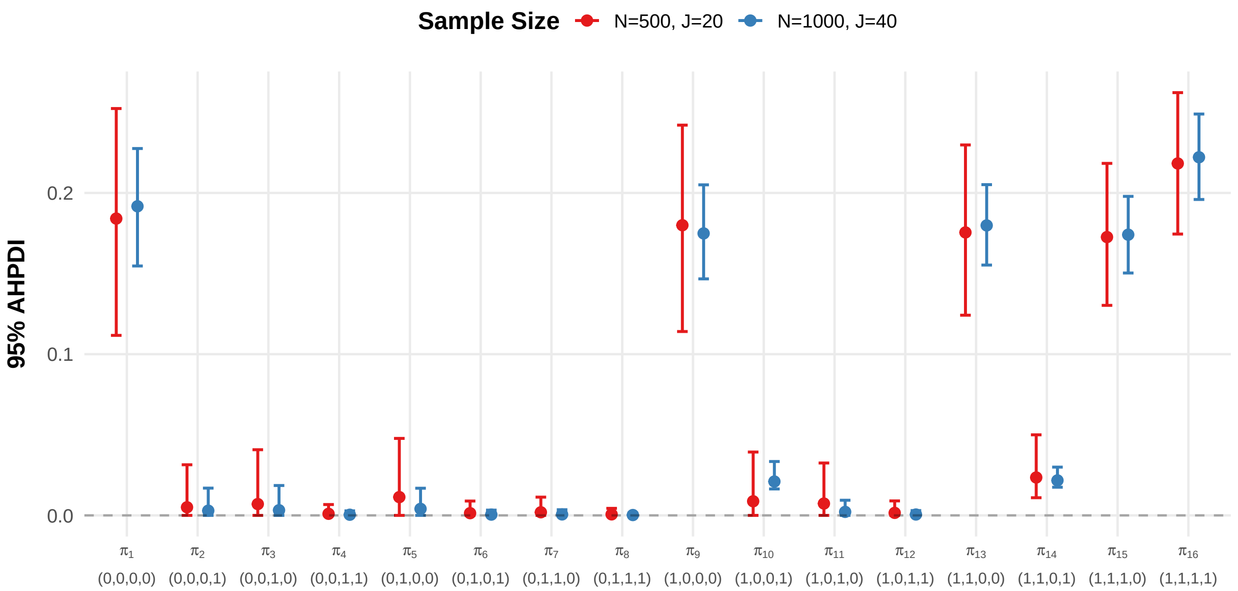

(a) G_1, Balanced

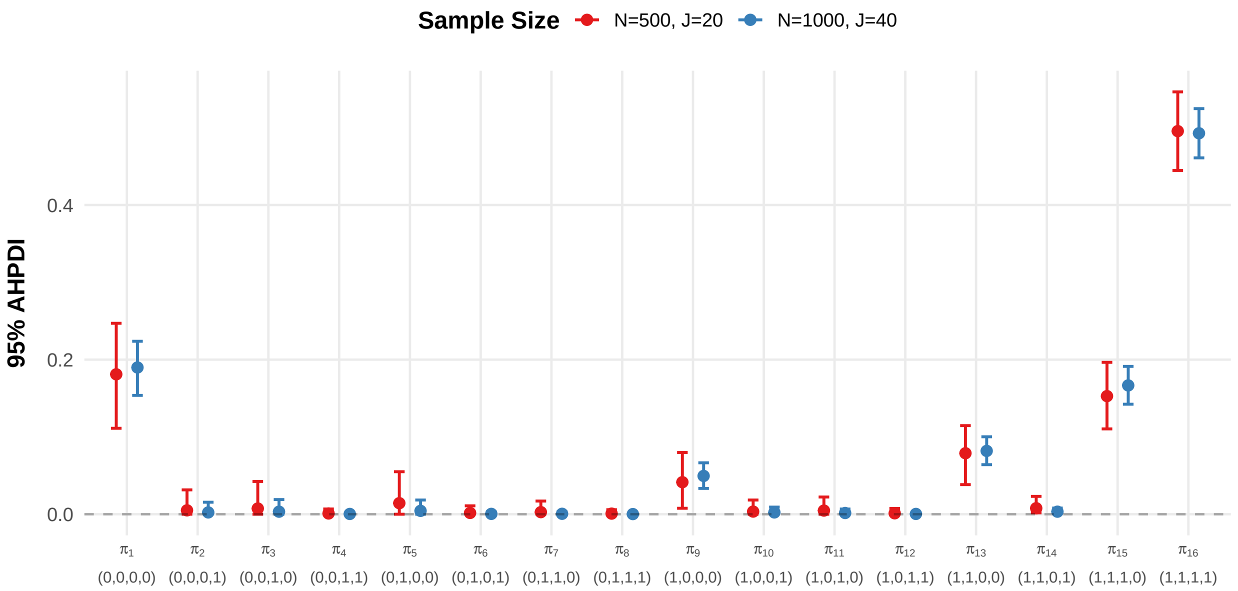

(b) G_1, Unbalanced

Figure 3: 95% AHPDI for the parameter $\boldsymbol{\pi}$ under hierarchy structure G_1.

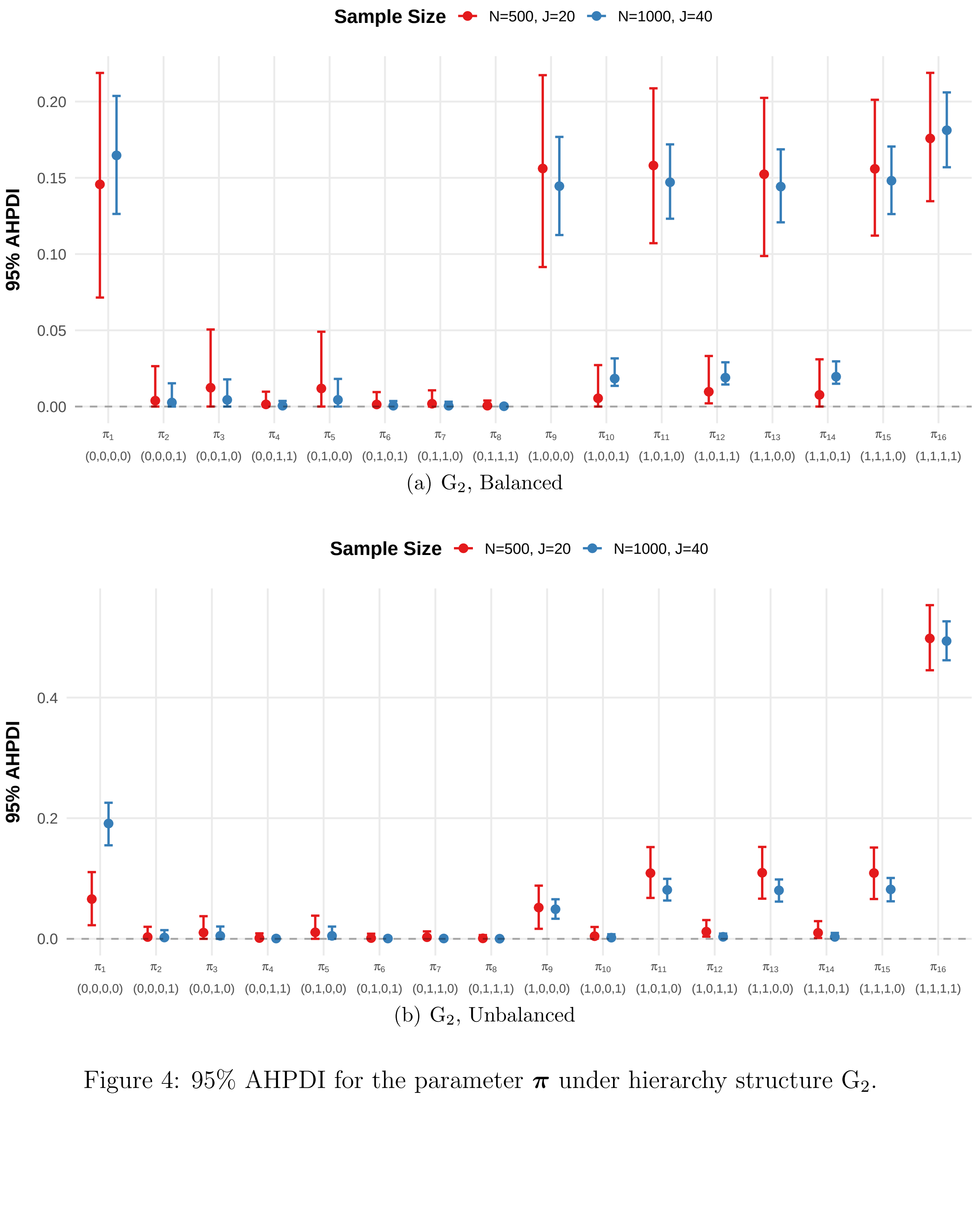

(a) G_2, Balanced

(b) G_2, Unbalanced

Figure 4: 95% AHPDI for the parameter $\boldsymbol{\pi}$ under hierarchy structure G_2.

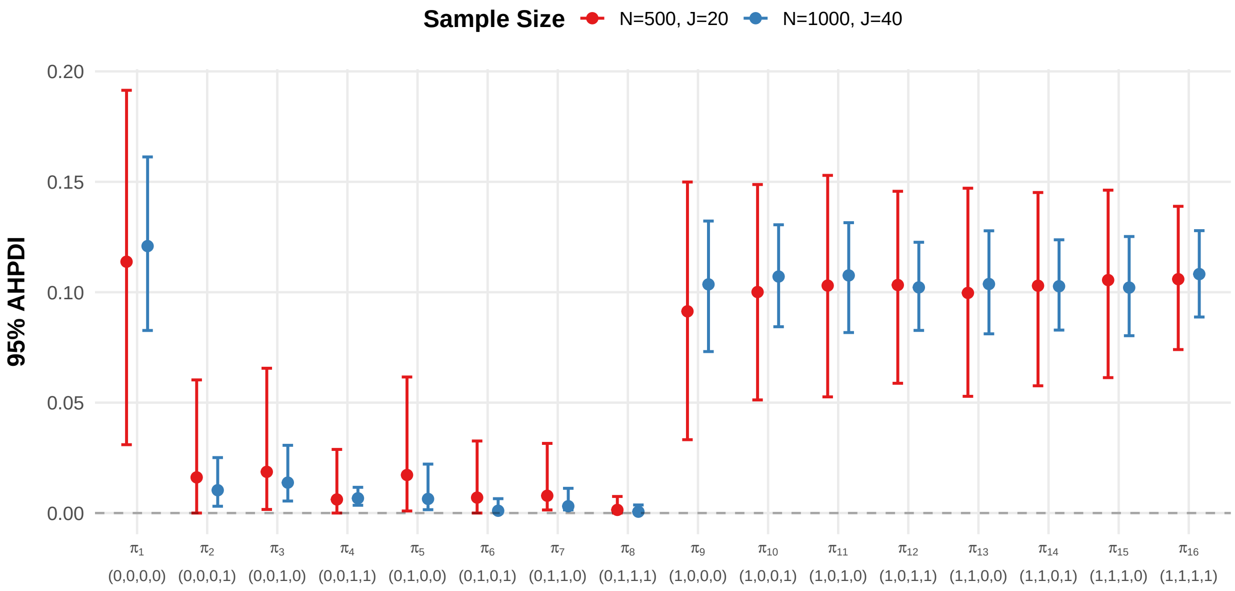

(a) G_3, Balanced

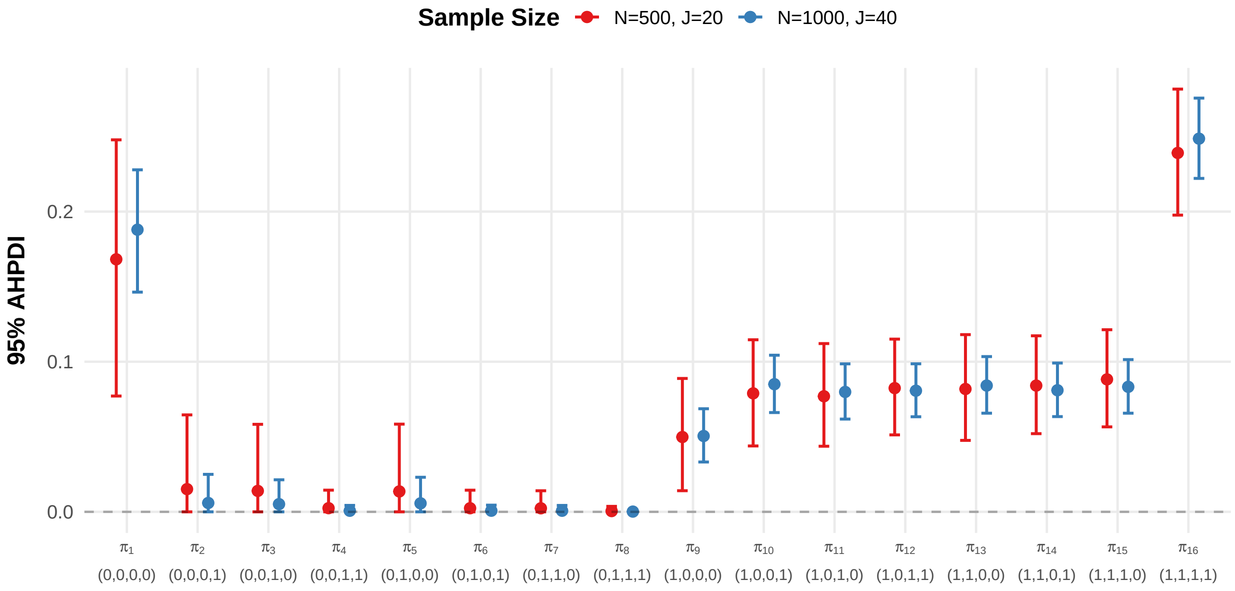

(b) G_3, Unbalanced

Figure 5: 95% AHPDI for the parameter $\boldsymbol{\pi}$ under hierarchy structure G_3.

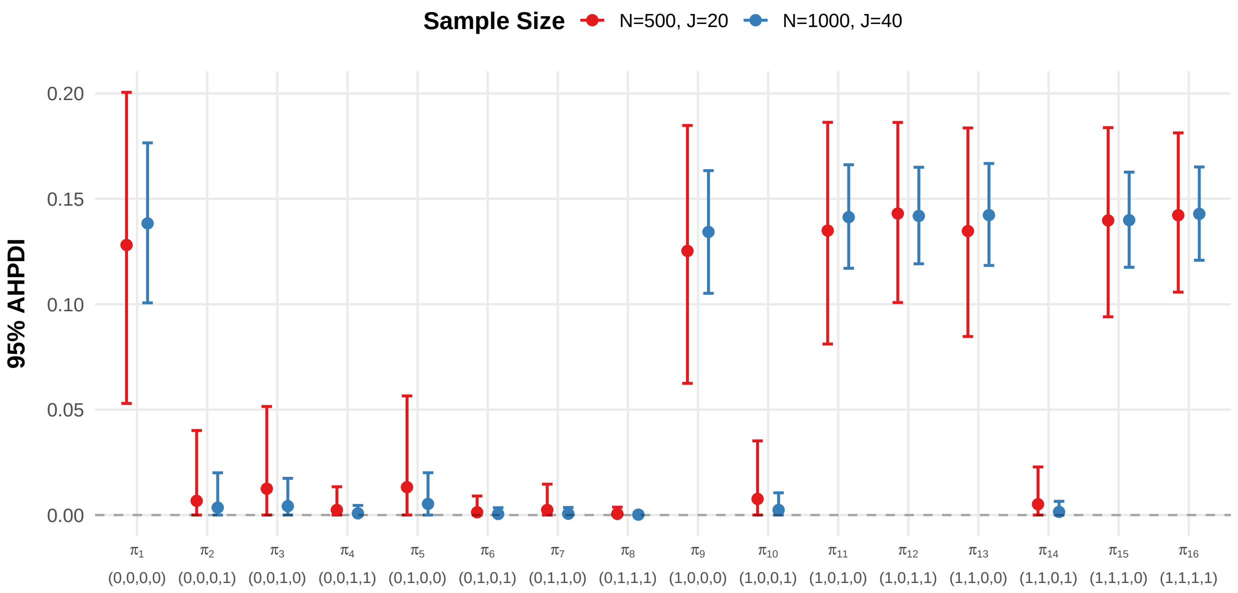

(a) G_4, Balanced

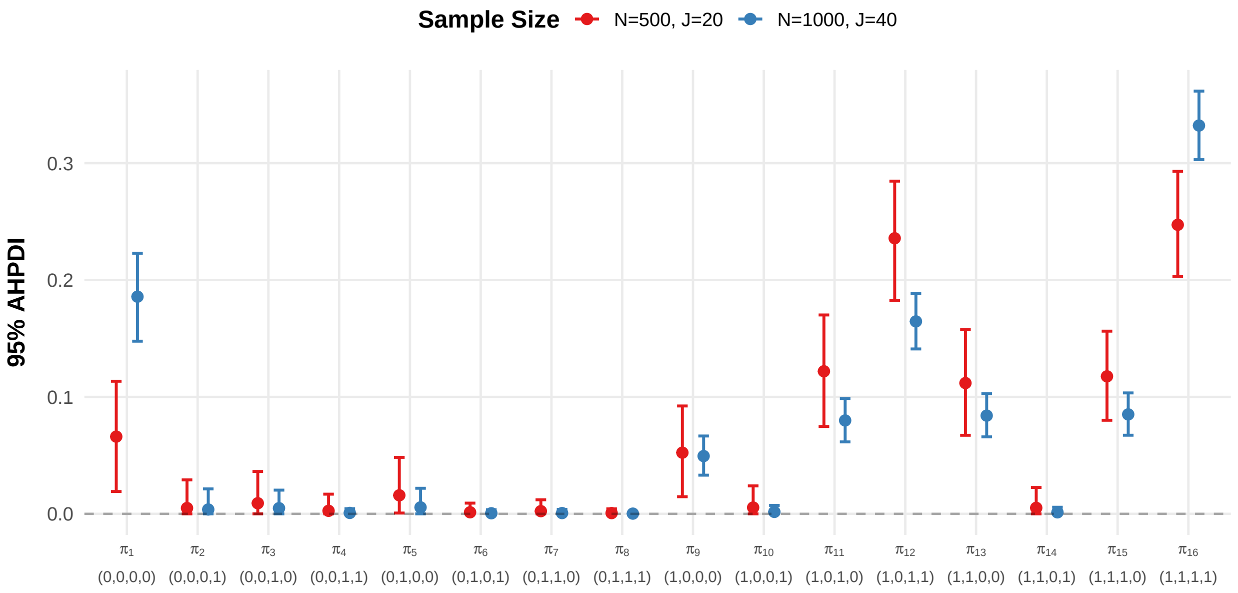

(b) G_4, Unbalanced

Figure 6: 95% AHPDI for the parameter $\boldsymbol{\pi}$ under the hierarchy structure G_4.
