# Supplementary figures and images for: Uncovering Latent Structures: A Bayesian Approach to Estimating Q-Matrix and Attribute Hierarchies in Cognitive Diagnostic Models
Source: Psychometrika. 2026 Feb 20;91(2):669–94. doi: 10.1017/psy.2026.10093 (PMC13294626; doi:10.1017/psy.2026.10093)

**Sample Size** ● N=500, J=20 ● N=1000, J=40

**95% AHPDI**

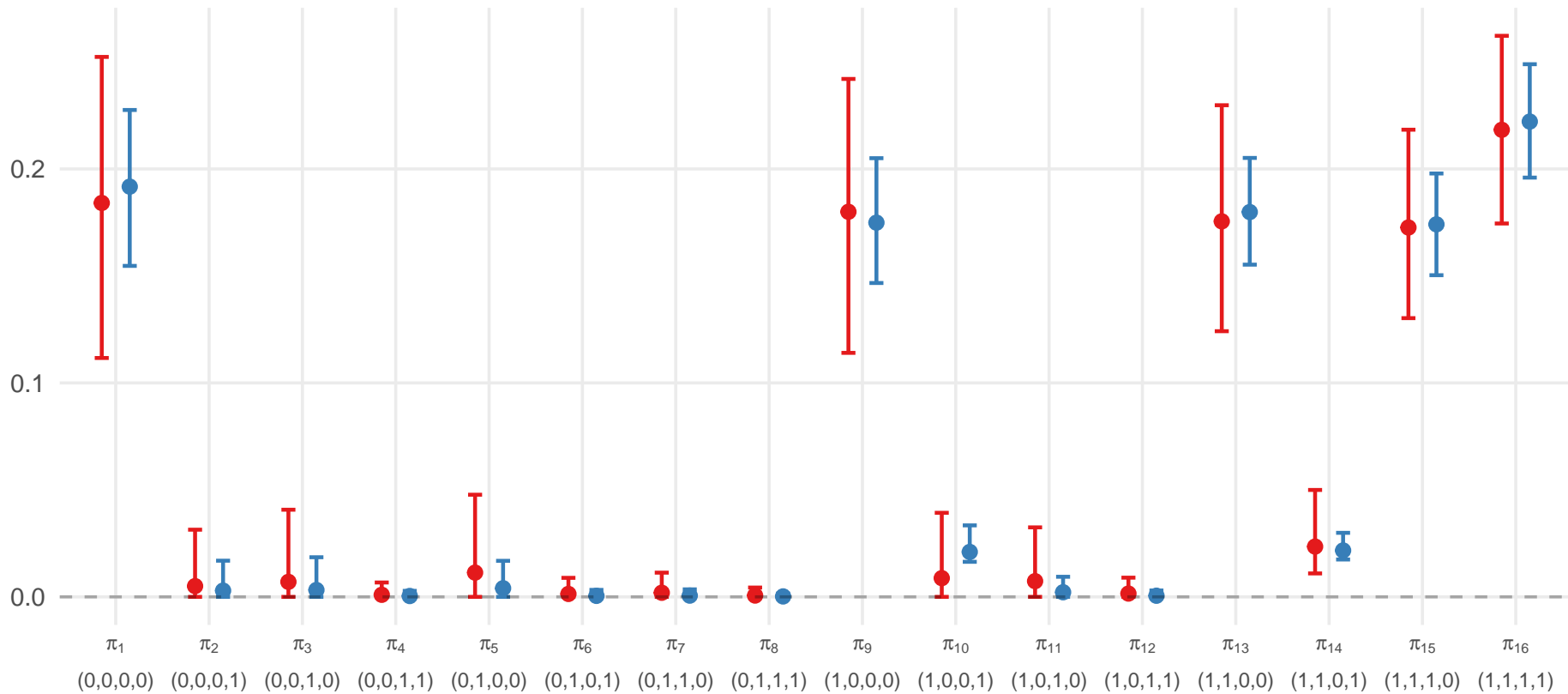

Supplement: Wang et al. supplementary material [file S0033312326100933sup001.zip › Appendix/plot/G1_balanced.pdf]

**g**

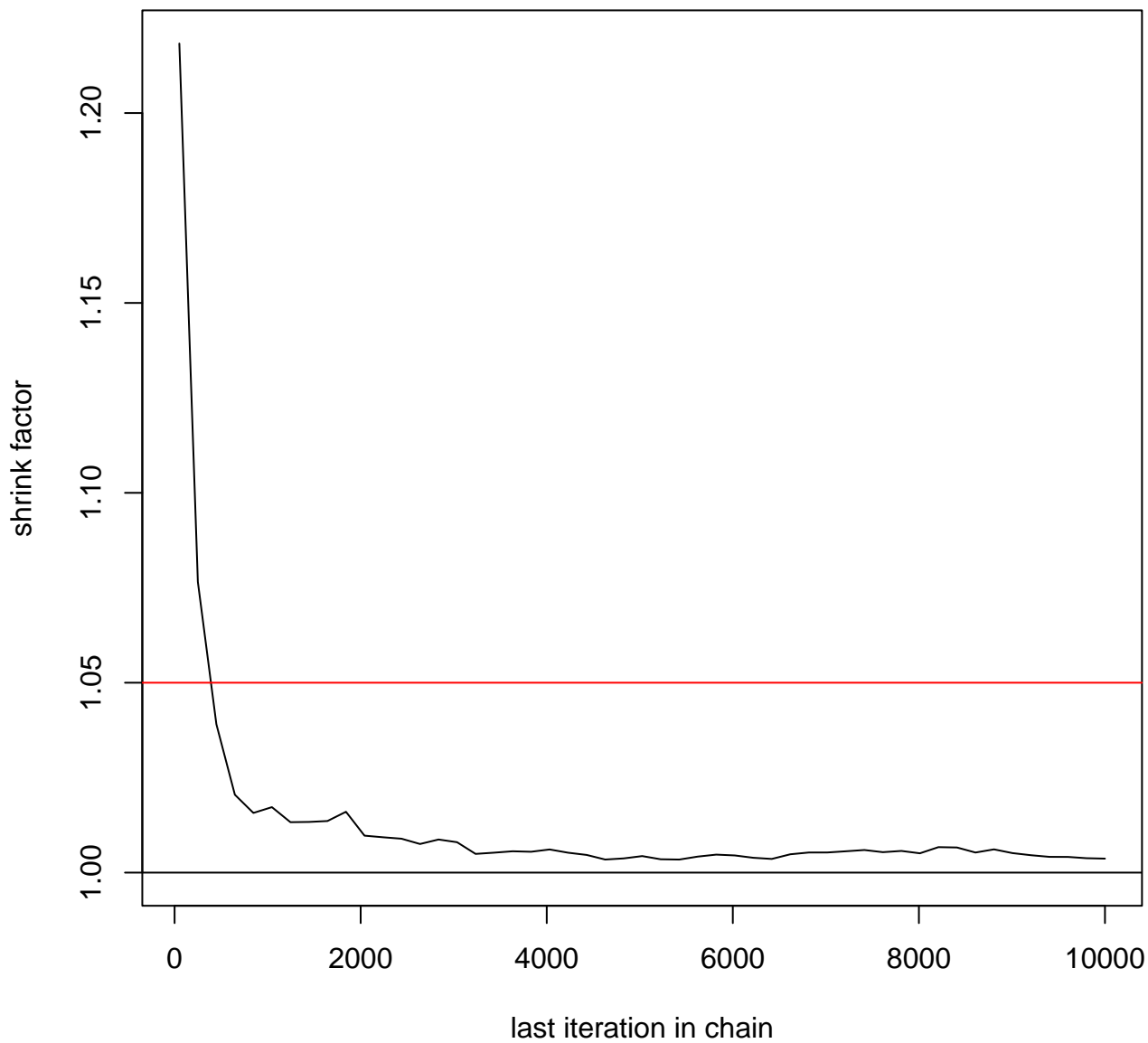

Supplement: Wang et al. supplementary material [file S0033312326100933sup001.zip › Appendix/plot/G1_N_500_J_20_sg_0.2_balanced_N1_256_g.pdf]

**S**

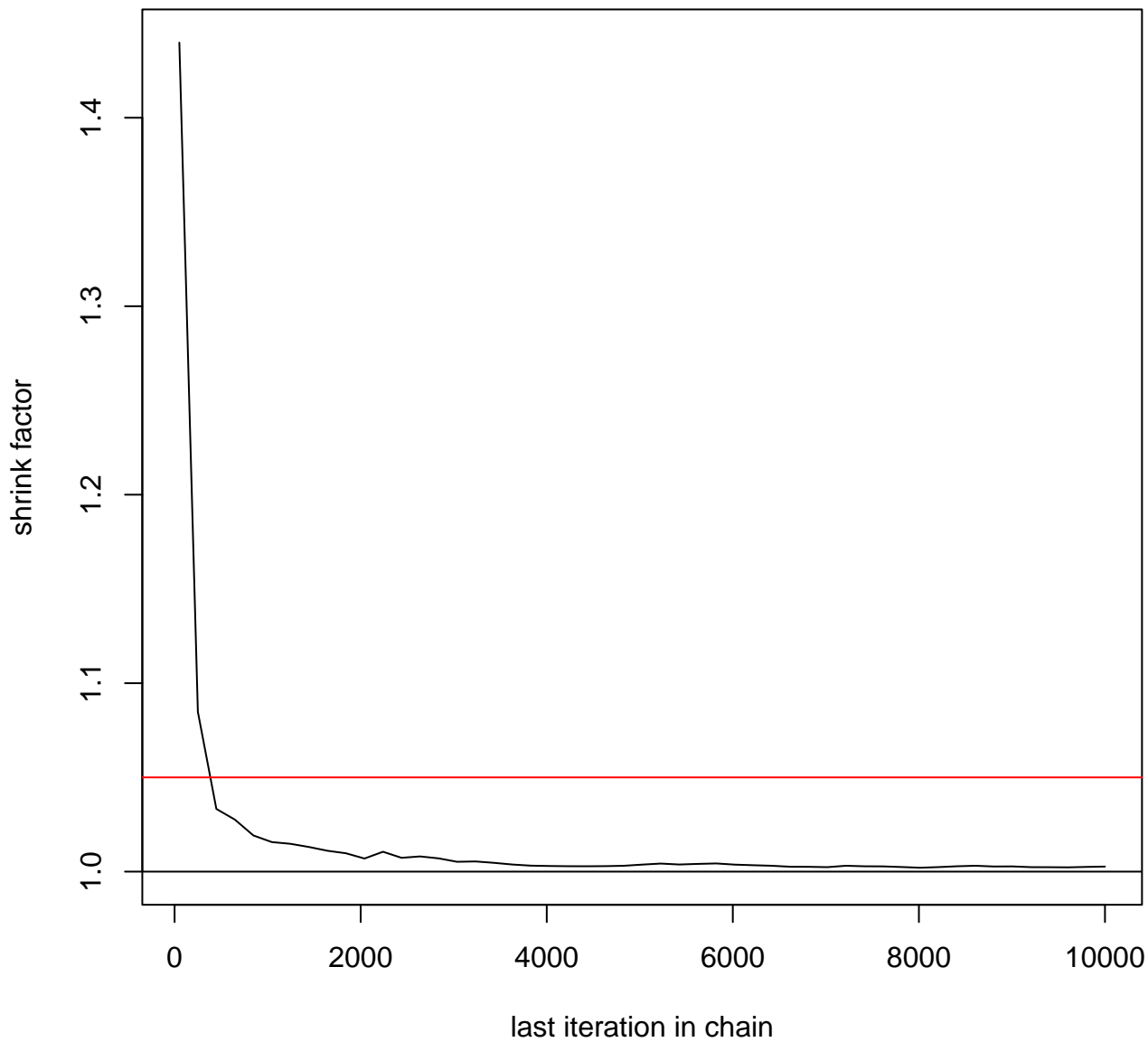

Supplement: Wang et al. supplementary material [file S0033312326100933sup001.zip › Appendix/plot/G1_N_500_J_20_sg_0.2_balanced_N1_256_s.pdf]

**g**

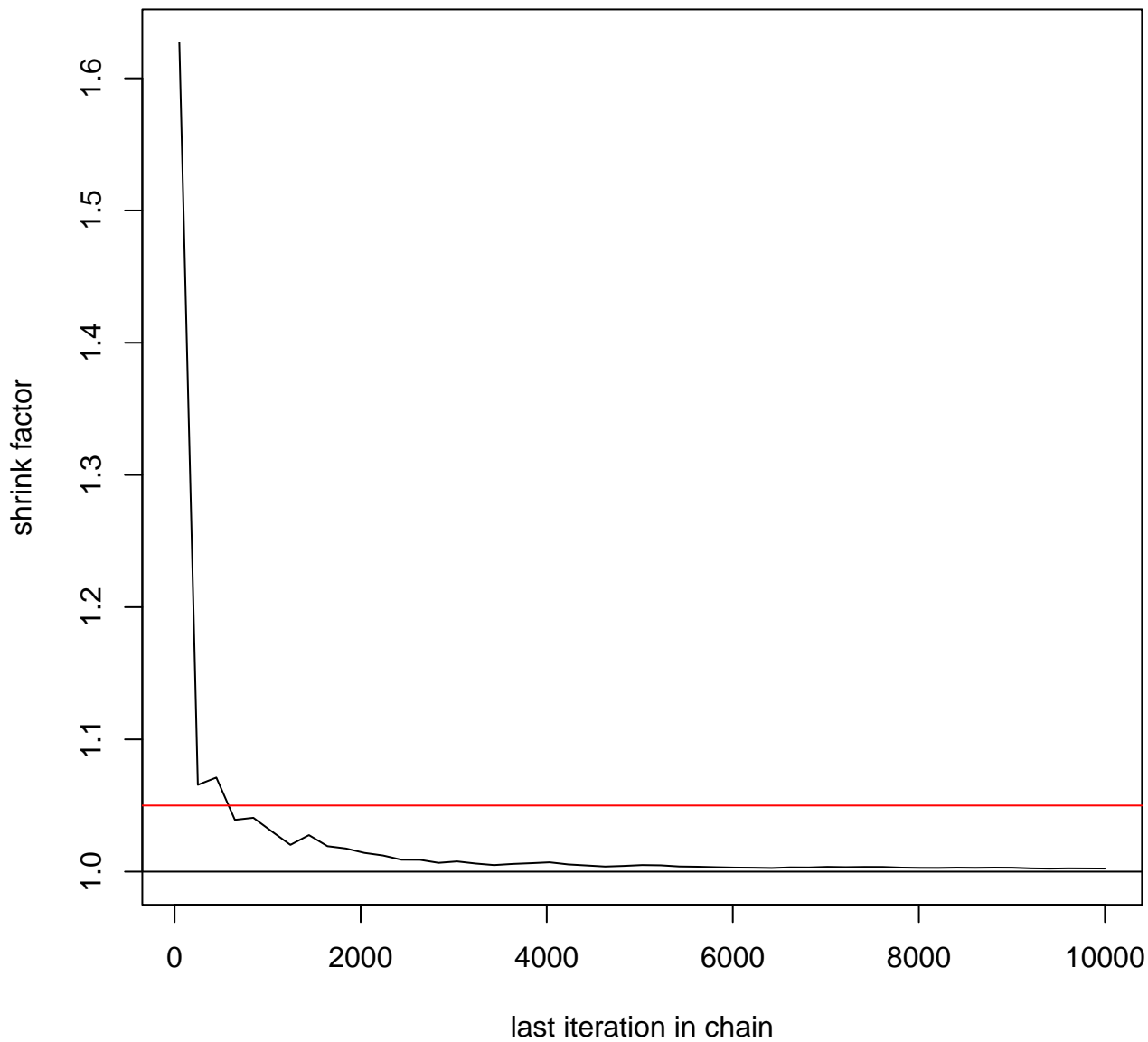

Supplement: Wang et al. supplementary material [file S0033312326100933sup001.zip › Appendix/plot/G1_N_500_J_20_sg_0.2_unbalanced_N1_256_g.pdf]

**S**

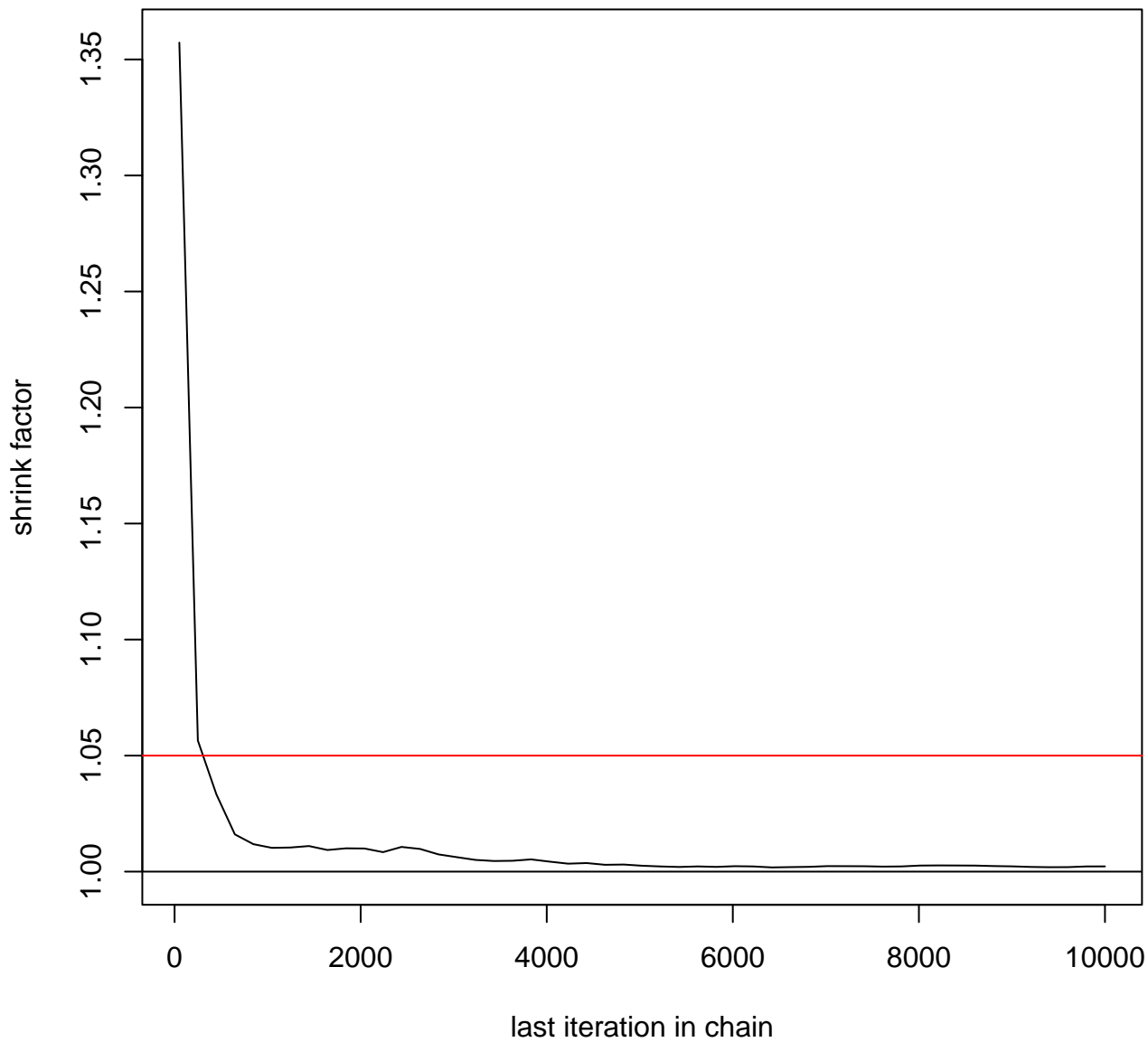

Supplement: Wang et al. supplementary material [file S0033312326100933sup001.zip › Appendix/plot/G1_N_500_J_20_sg_0.2_unbalanced_N1_256_s.pdf]

**Sample Size** ● N=500, J=20 ● N=1000, J=40

**95% AHPDI**

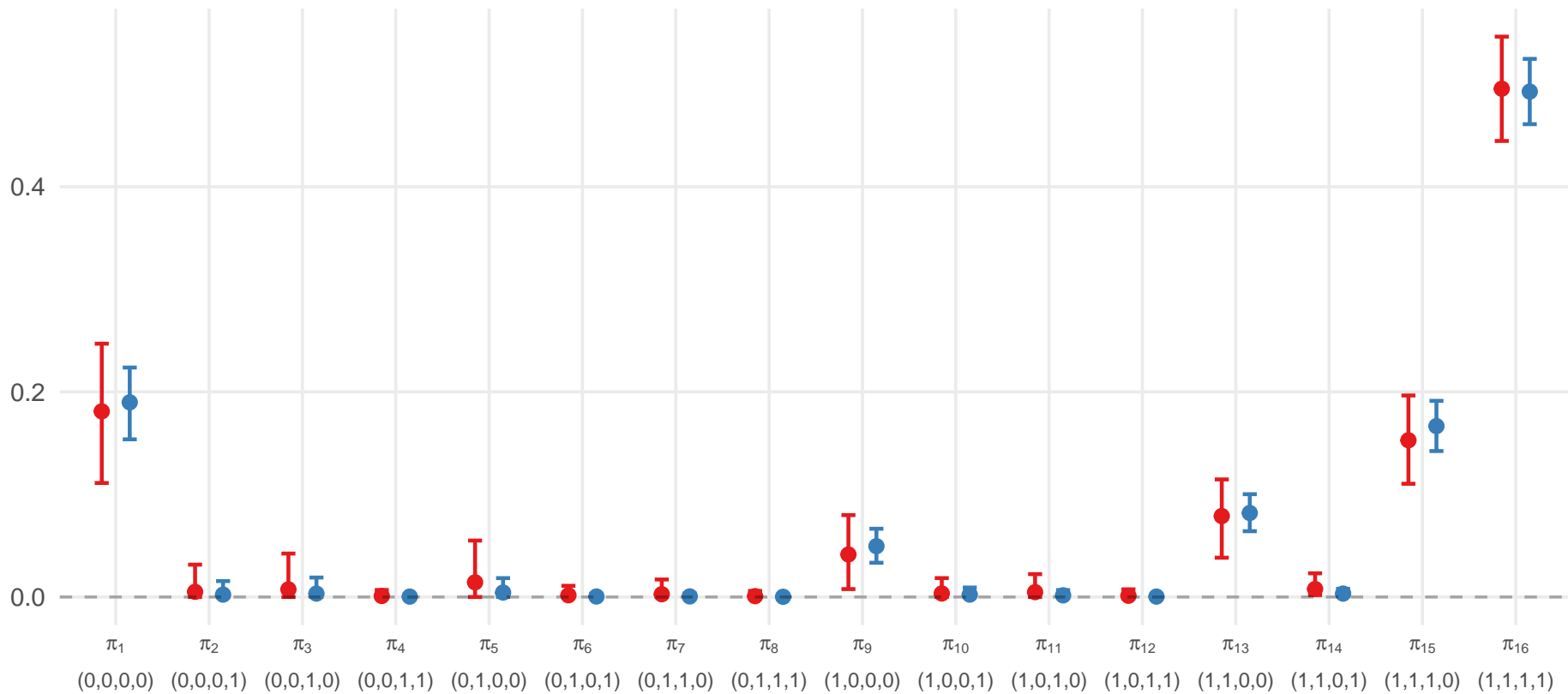

Supplement: Wang et al. supplementary material [file S0033312326100933sup001.zip › Appendix/plot/G1_unbalanced.pdf]

**Sample Size** ● N=500, J=20 ● N=1000, J=40

**95% AHPDI**

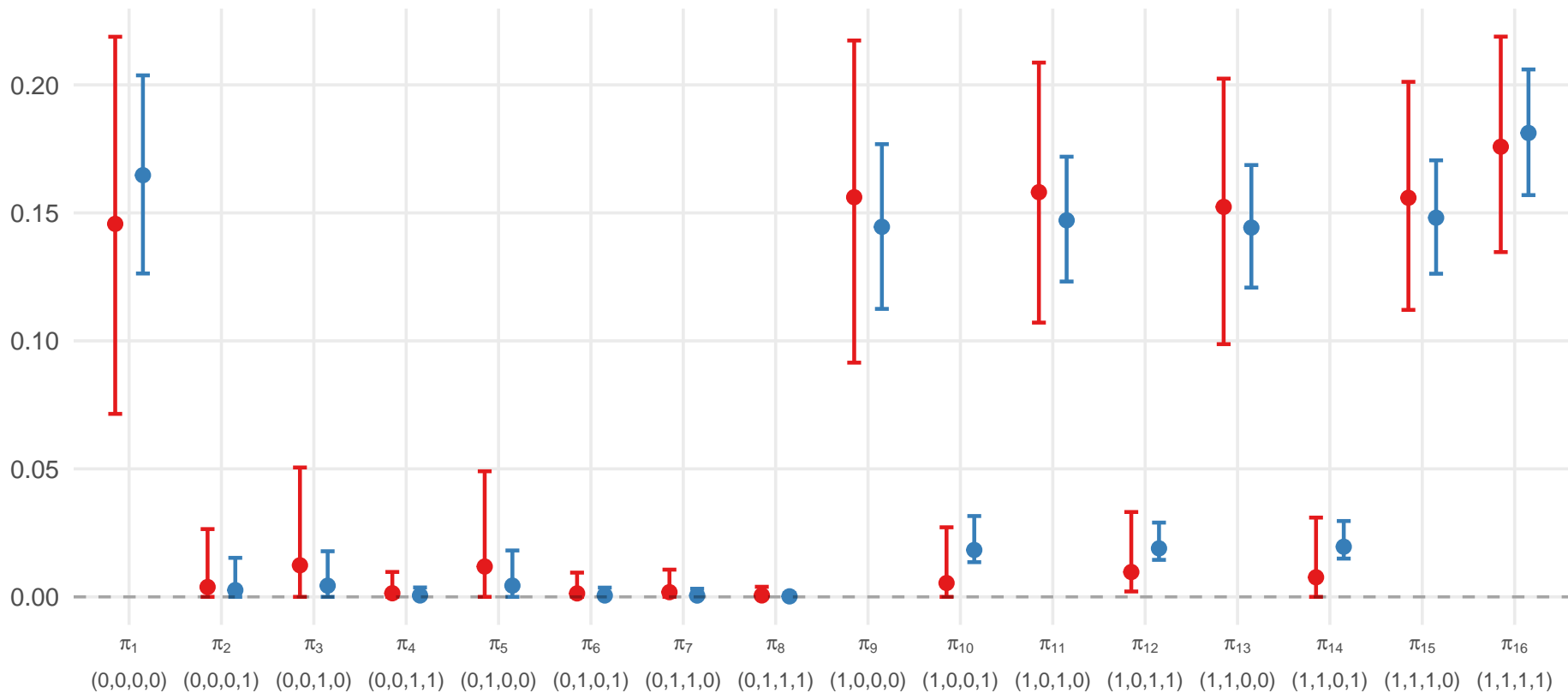

Supplement: Wang et al. supplementary material [file S0033312326100933sup001.zip › Appendix/plot/G2_balanced.pdf]

**g**

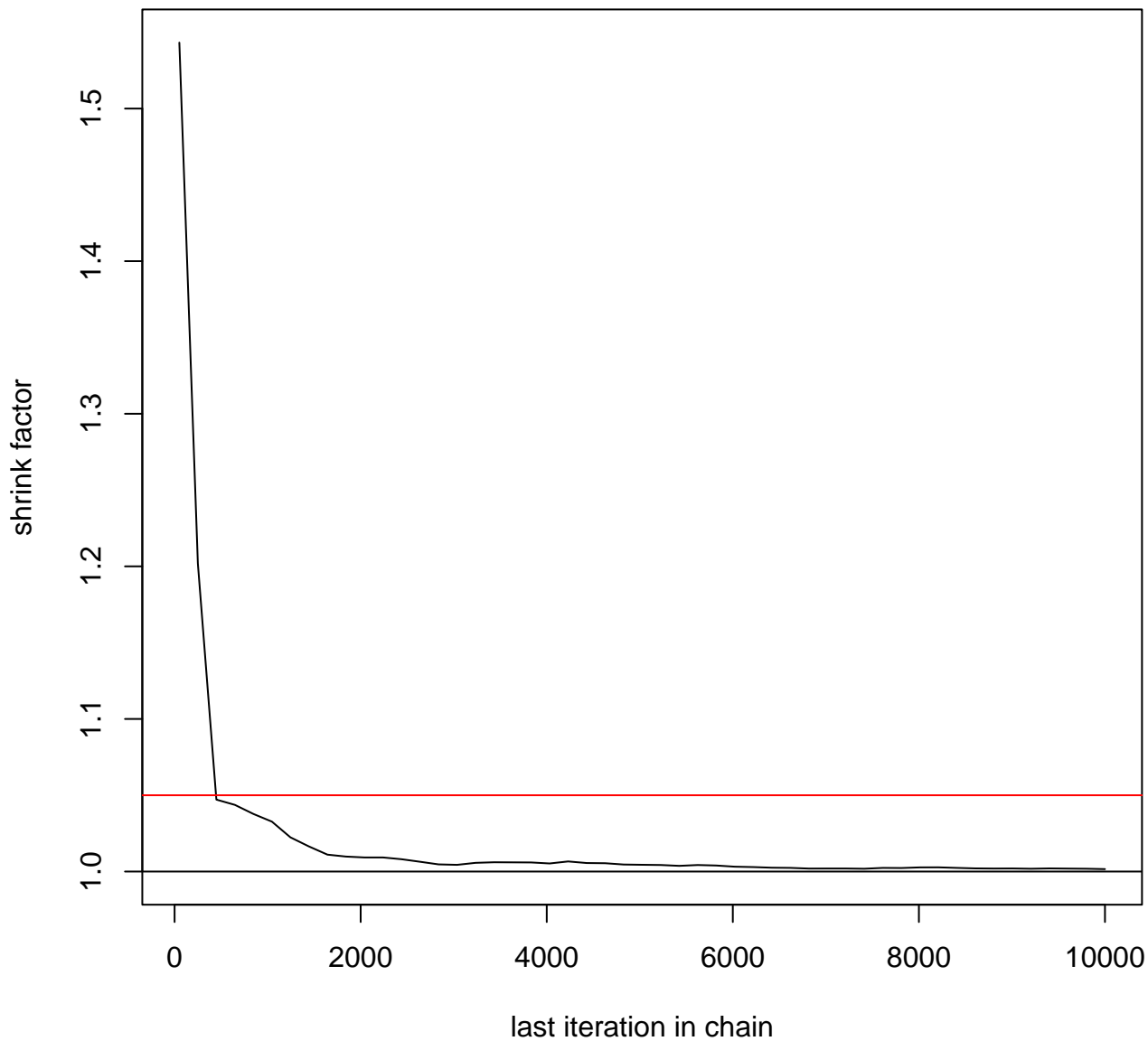

Supplement: Wang et al. supplementary material [file S0033312326100933sup001.zip › Appendix/plot/G2_N_500_J_20_sg_0.2_balanced_N1_256_g.pdf]

**S**

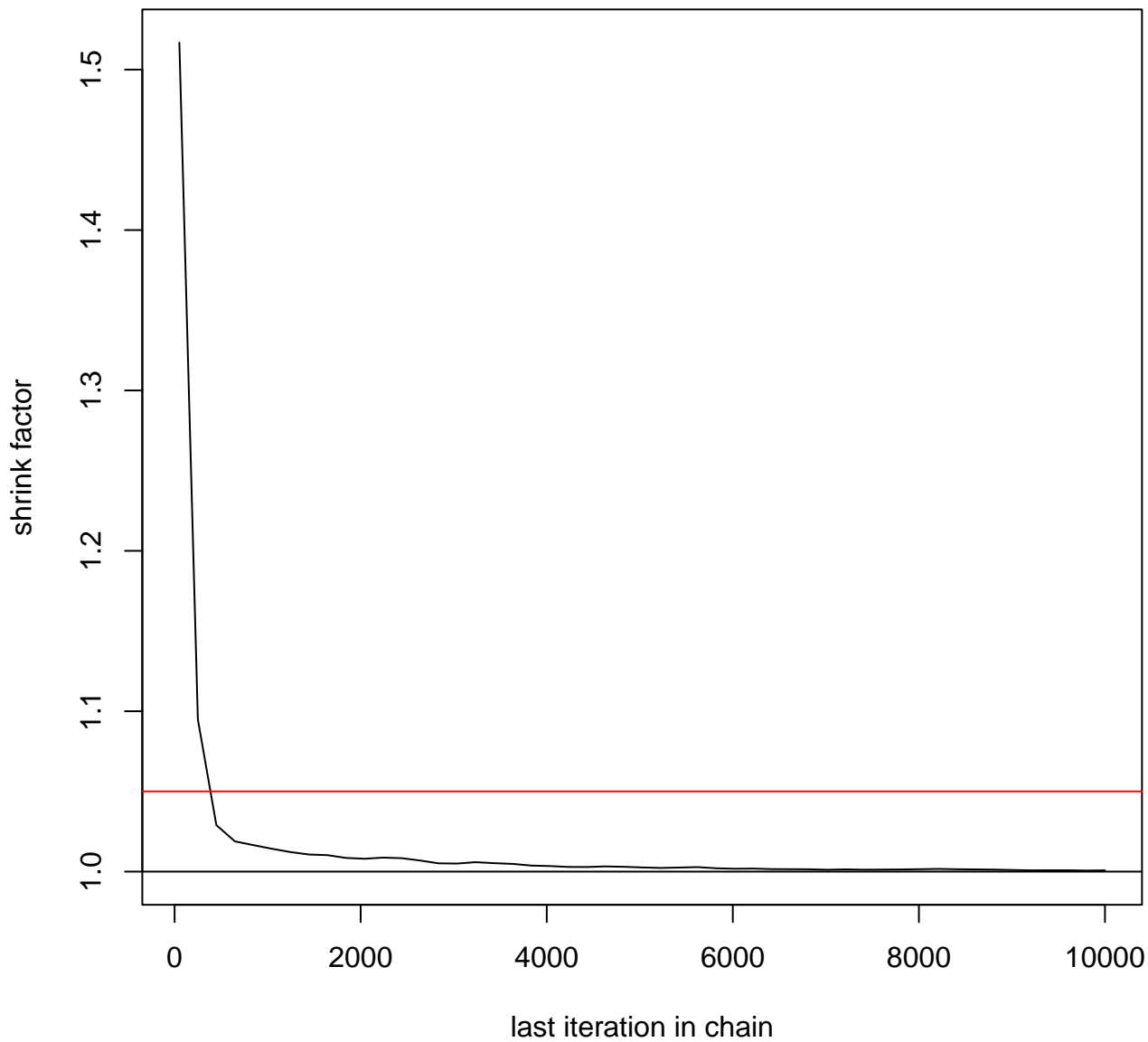

Supplement: Wang et al. supplementary material [file S0033312326100933sup001.zip › Appendix/plot/G2_N_500_J_20_sg_0.2_balanced_N1_256_s.pdf]

**g**

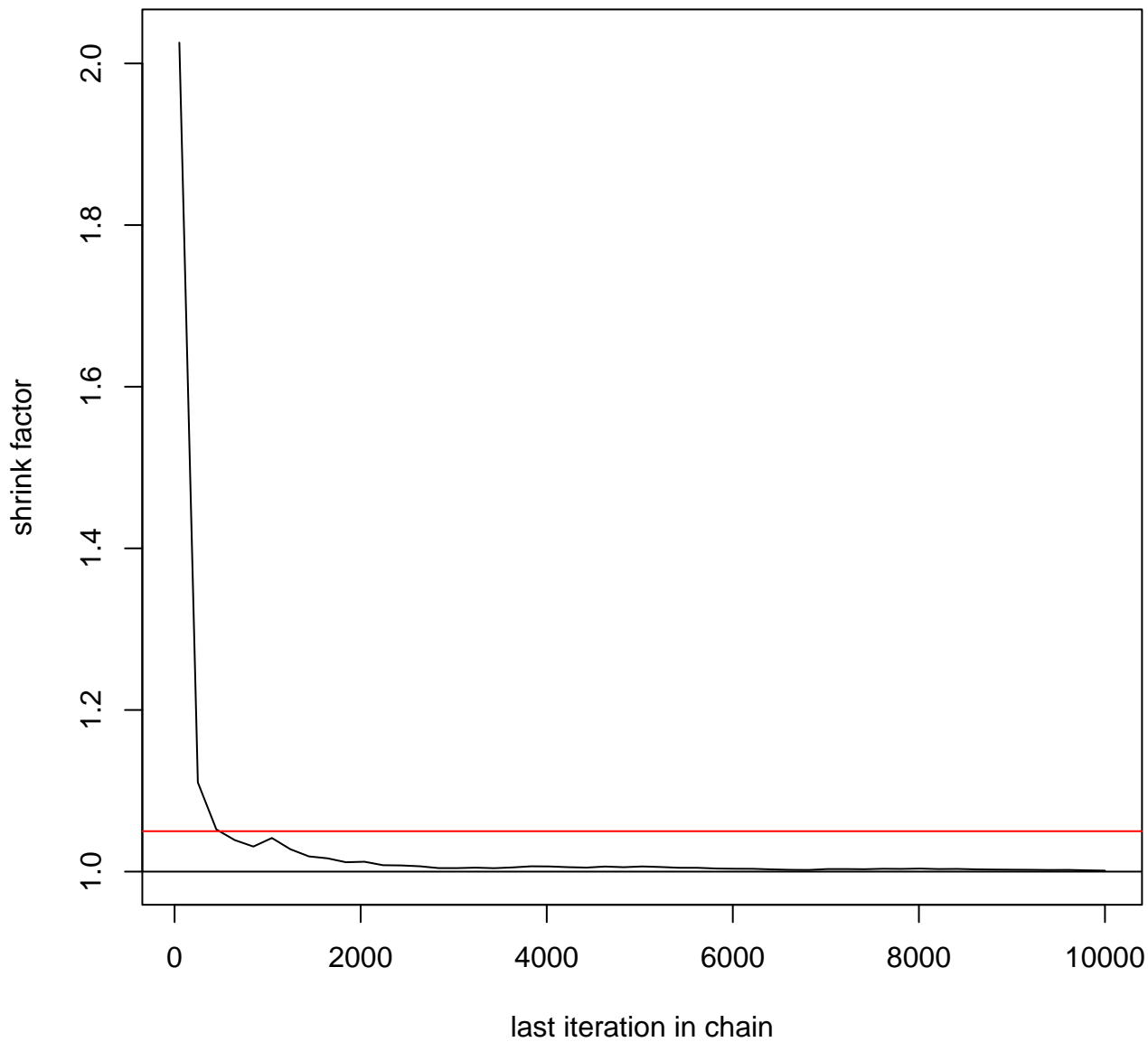

Supplement: Wang et al. supplementary material [file S0033312326100933sup001.zip › Appendix/plot/G2_N_500_J_20_sg_0.2_unbalanced_N1_256_g.pdf]

**S**

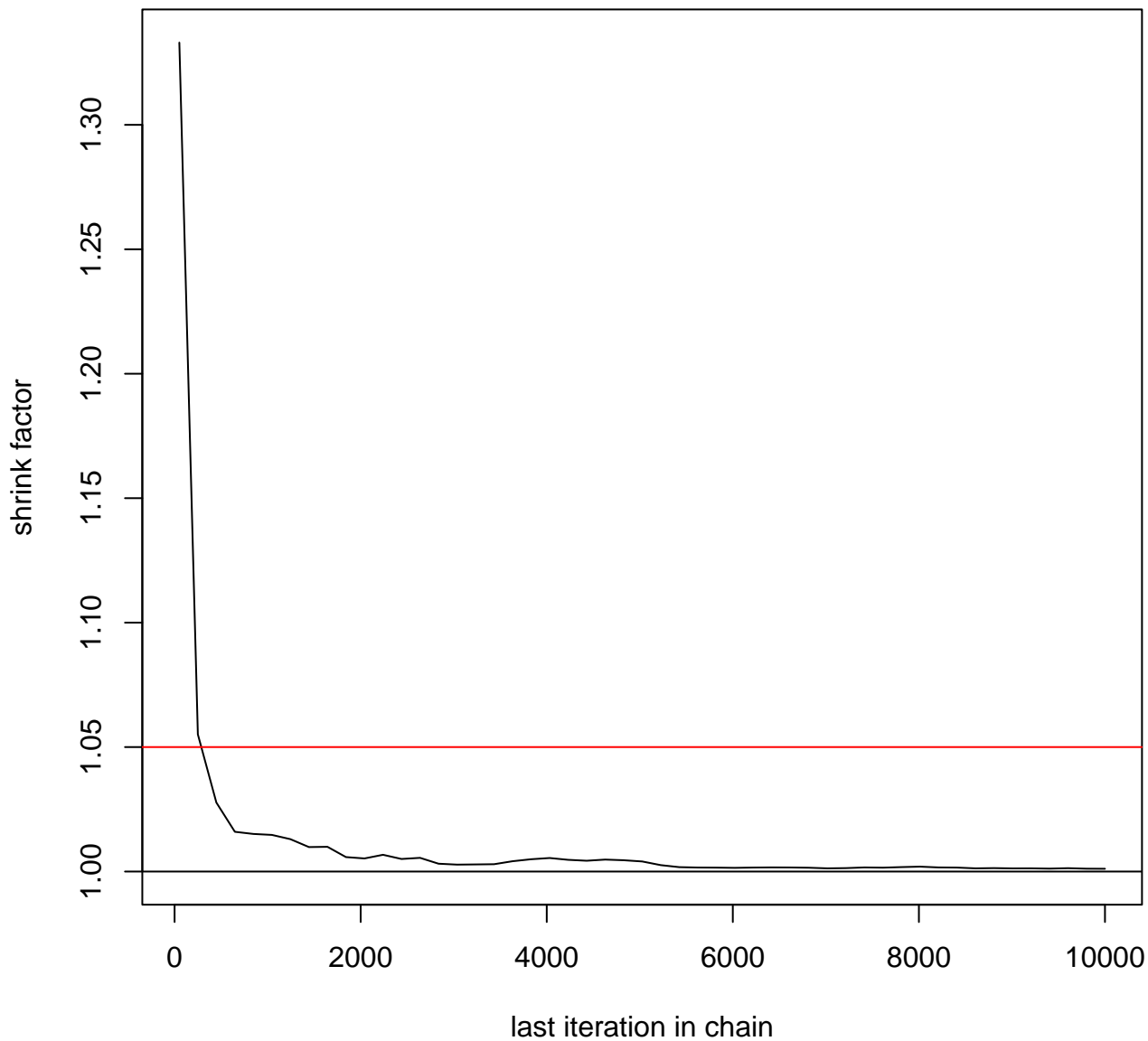

Supplement: Wang et al. supplementary material [file S0033312326100933sup001.zip › Appendix/plot/G2_N_500_J_20_sg_0.2_unbalanced_N1_256_s.pdf]

**Sample Size** ● N=500, J=20 ● N=1000, J=40

**95% AHPDI**

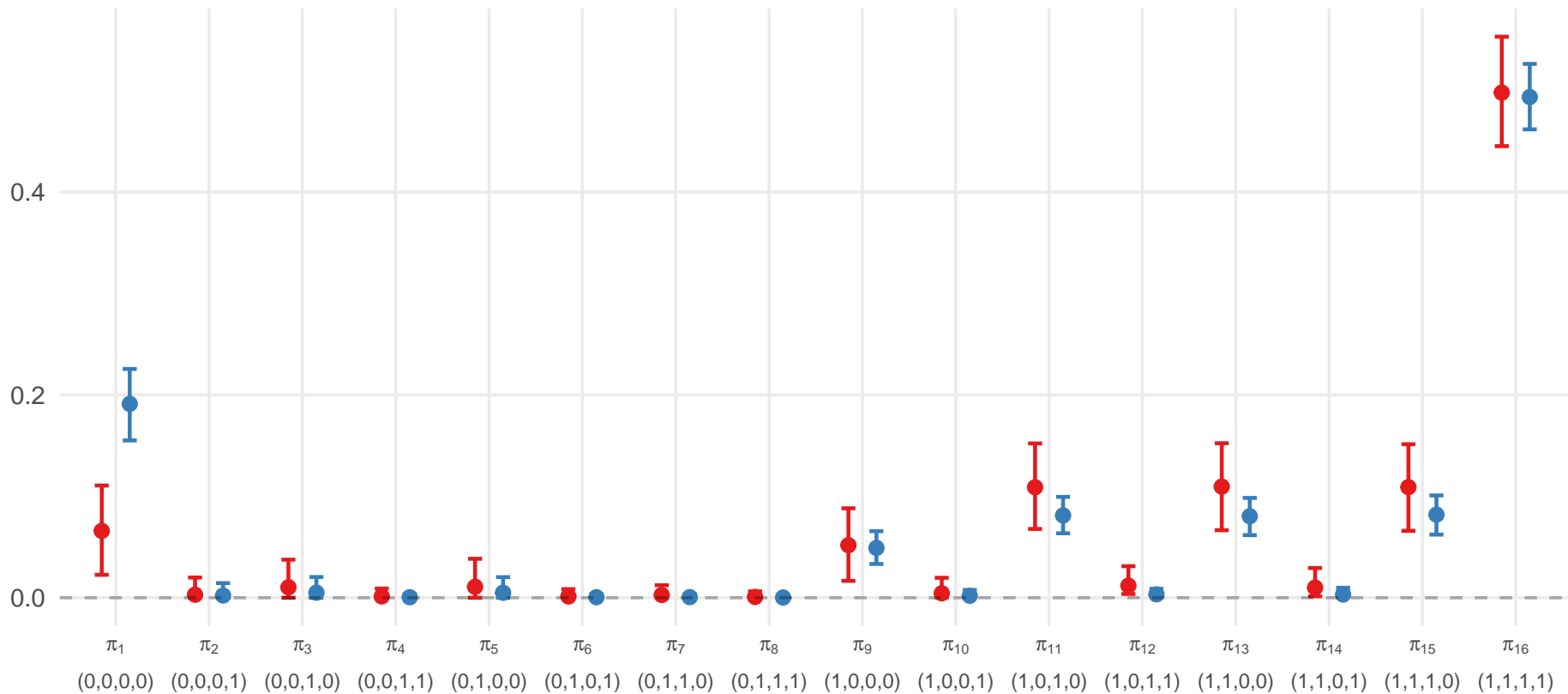

Supplement: Wang et al. supplementary material [file S0033312326100933sup001.zip › Appendix/plot/G2_unbalanced.pdf]

**Sample Size** ● N=500, J=20 ● N=1000, J=40

**95% AHPDI**

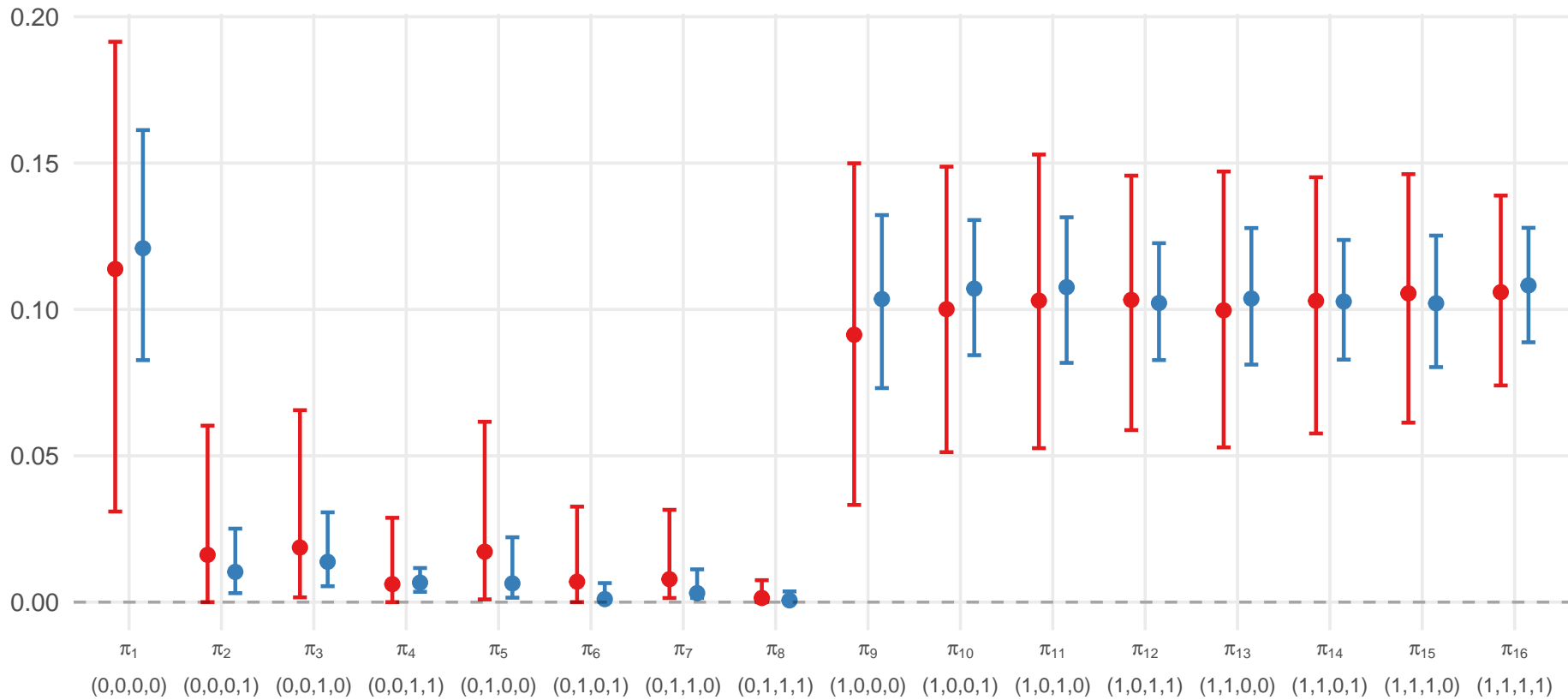

Supplement: Wang et al. supplementary material [file S0033312326100933sup001.zip › Appendix/plot/G3_balanced.pdf]

**g**

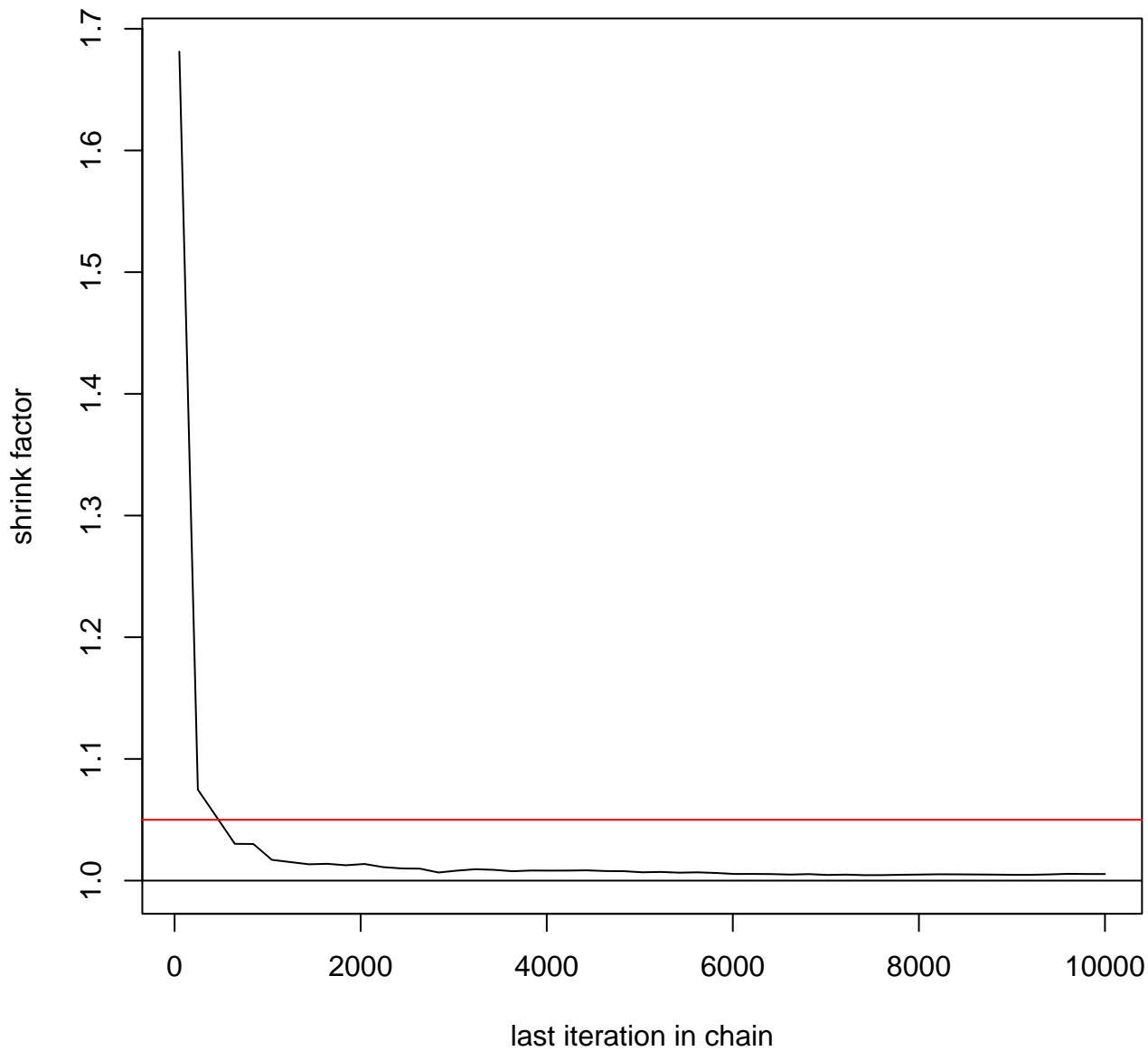

Supplement: Wang et al. supplementary material [file S0033312326100933sup001.zip › Appendix/plot/G3_N_1000_J_40_sg_0.2_balanced_N1_256_g.pdf]

**g**

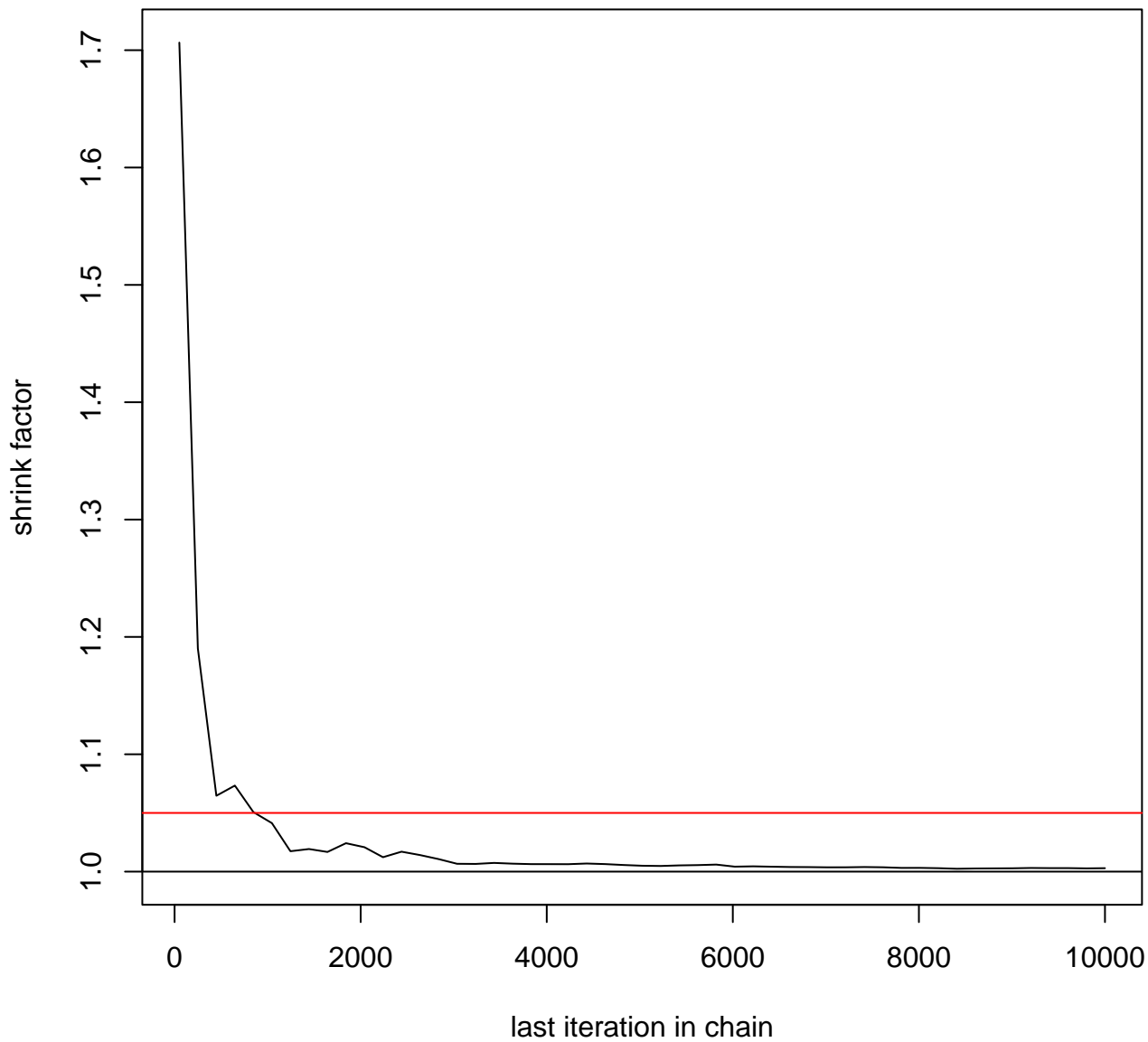

Supplement: Wang et al. supplementary material [file S0033312326100933sup001.zip › Appendix/plot/G3_N_500_J_20_sg_0.2_balanced_N1_256_g.pdf]

**S**

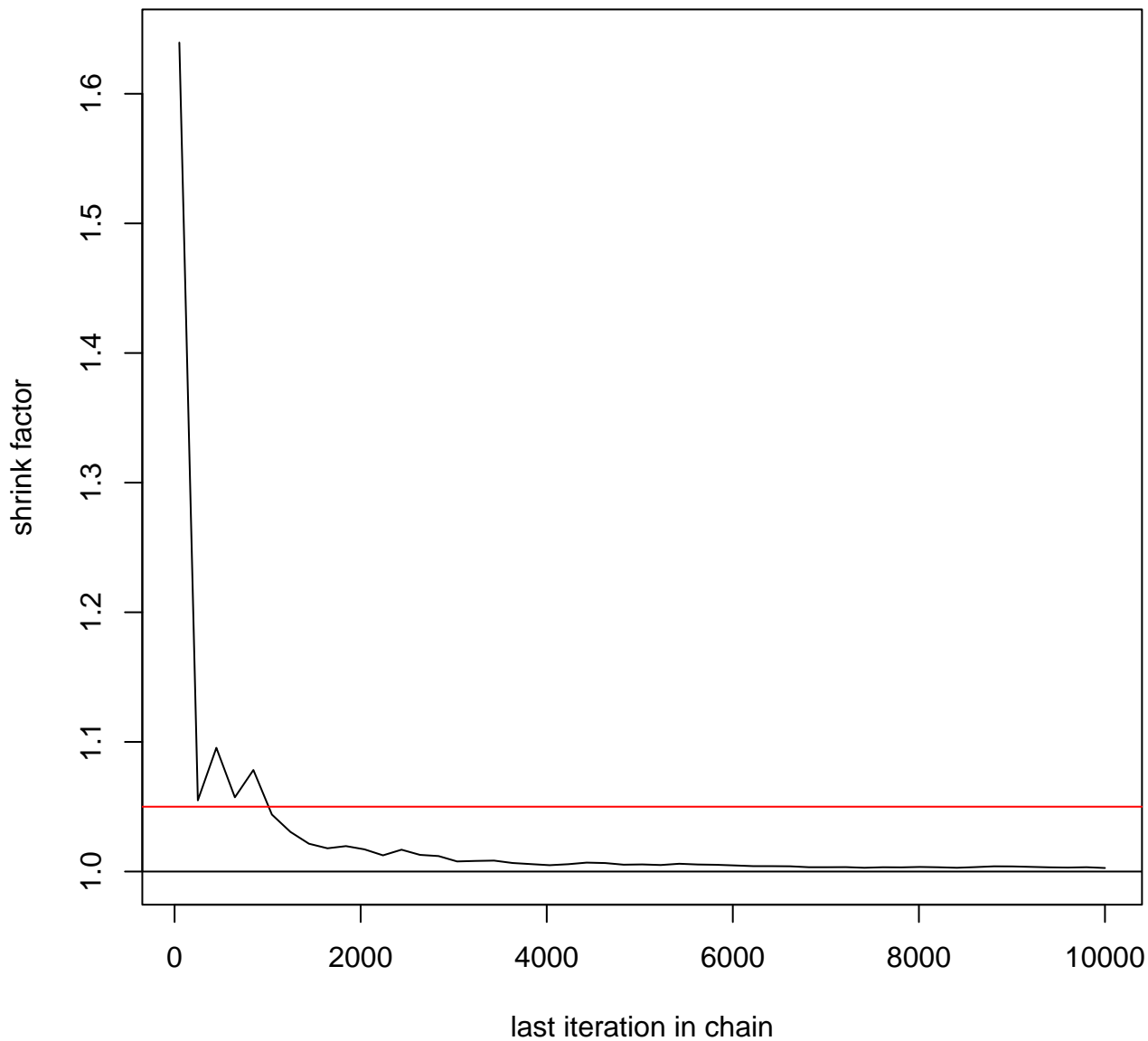

Supplement: Wang et al. supplementary material [file S0033312326100933sup001.zip › Appendix/plot/G3_N_500_J_20_sg_0.2_balanced_N1_256_s.pdf]

**g**

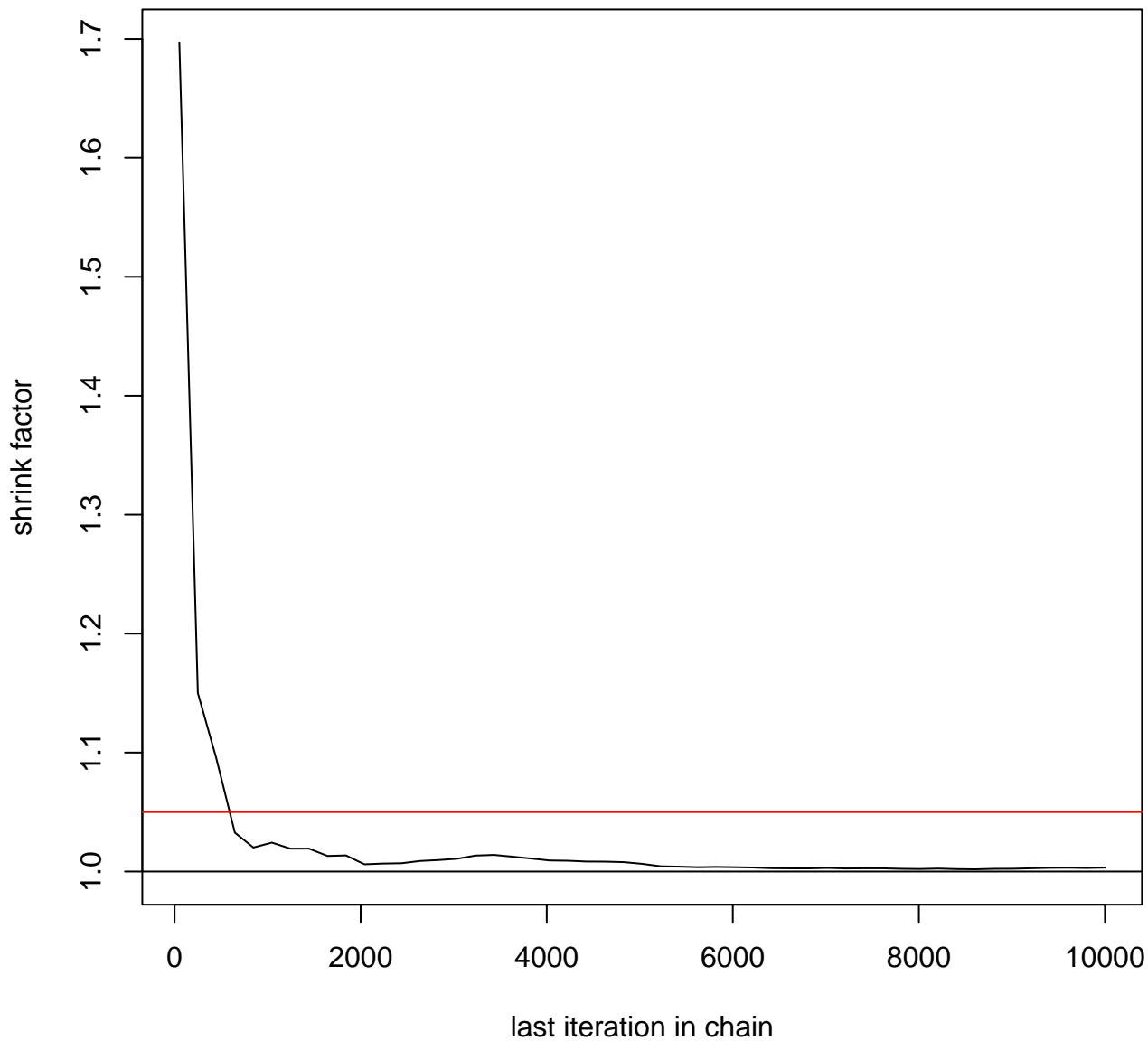

Supplement: Wang et al. supplementary material [file S0033312326100933sup001.zip › Appendix/plot/G3_N_500_J_20_sg_0.2_unbalanced_N1_256_g.pdf]

**S**

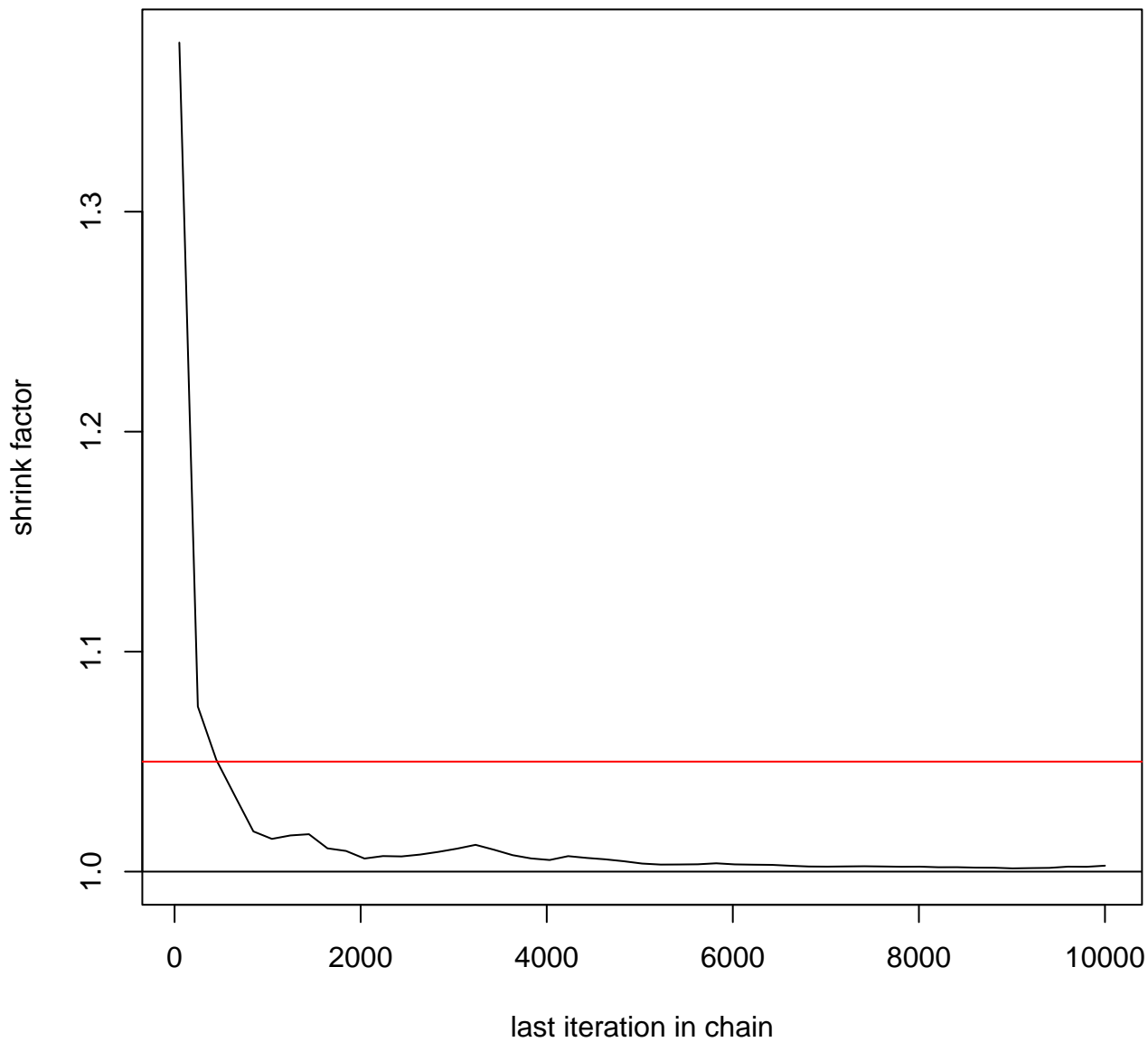

Supplement: Wang et al. supplementary material [file S0033312326100933sup001.zip › Appendix/plot/G3_N_500_J_20_sg_0.2_unbalanced_N1_256_s.pdf]

**Sample Size** ● N=500, J=20 ● N=1000, J=40

**95% AHPDI**

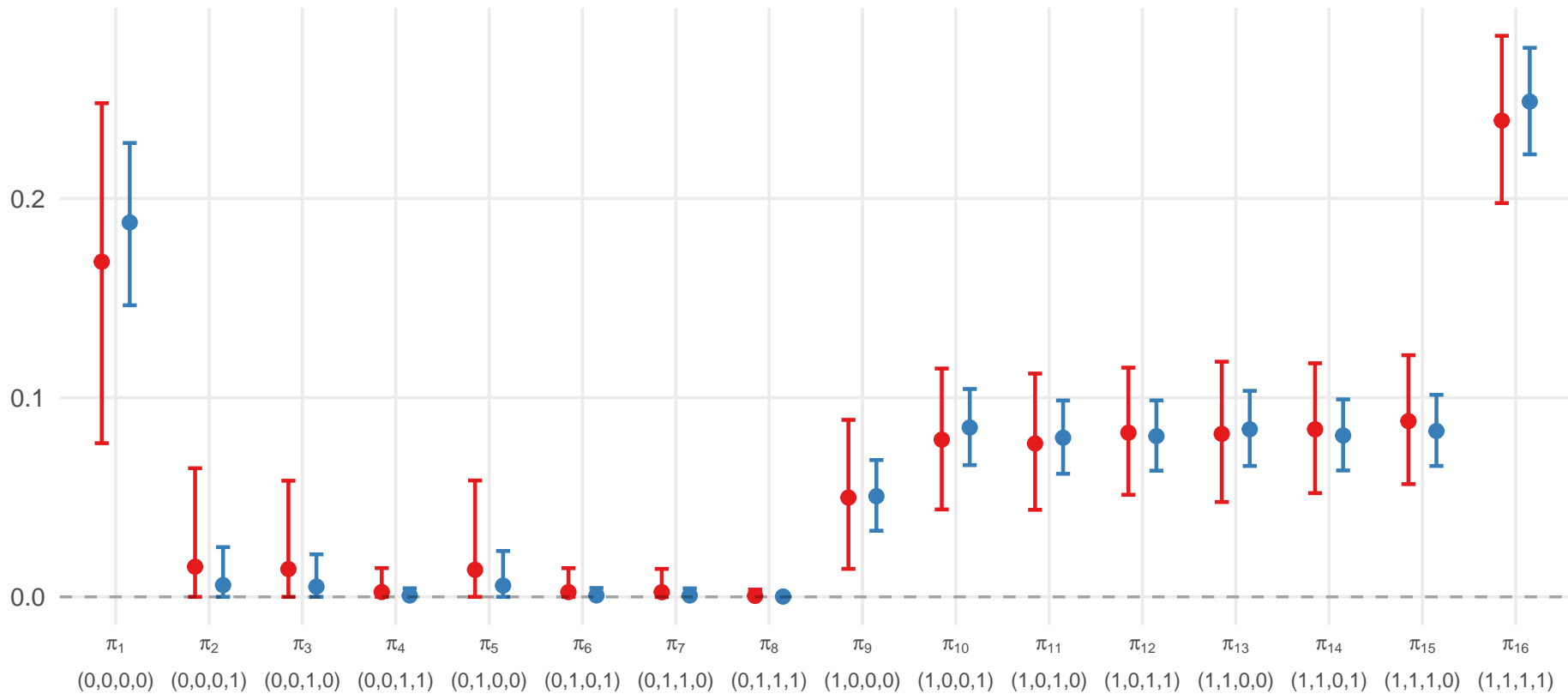

Supplement: Wang et al. supplementary material [file S0033312326100933sup001.zip › Appendix/plot/G3_unbalanced.pdf]

**Sample Size** ● N=500, J=20 ● N=1000, J=40

**95% AHPDI**

0.20  
0.15  
0.10  
0.05  
0.00

$\pi_1$   $\pi_2$   $\pi_3$   $\pi_4$   $\pi_5$   $\pi_6$   $\pi_7$   $\pi_8$   $\pi_9$   $\pi_{10}$   $\pi_{11}$   $\pi_{12}$   $\pi_{13}$   $\pi_{14}$   $\pi_{15}$   $\pi_{16}$   
(0,0,0,0) (0,0,0,1) (0,0,1,0) (0,0,1,1) (0,1,0,0) (0,1,0,1) (0,1,1,0) (0,1,1,1) (1,0,0,0) (1,0,0,1) (1,0,1,0) (1,0,1,1) (1,1,0,0) (1,1,0,1) (1,1,1,0) (1,1,1,1)

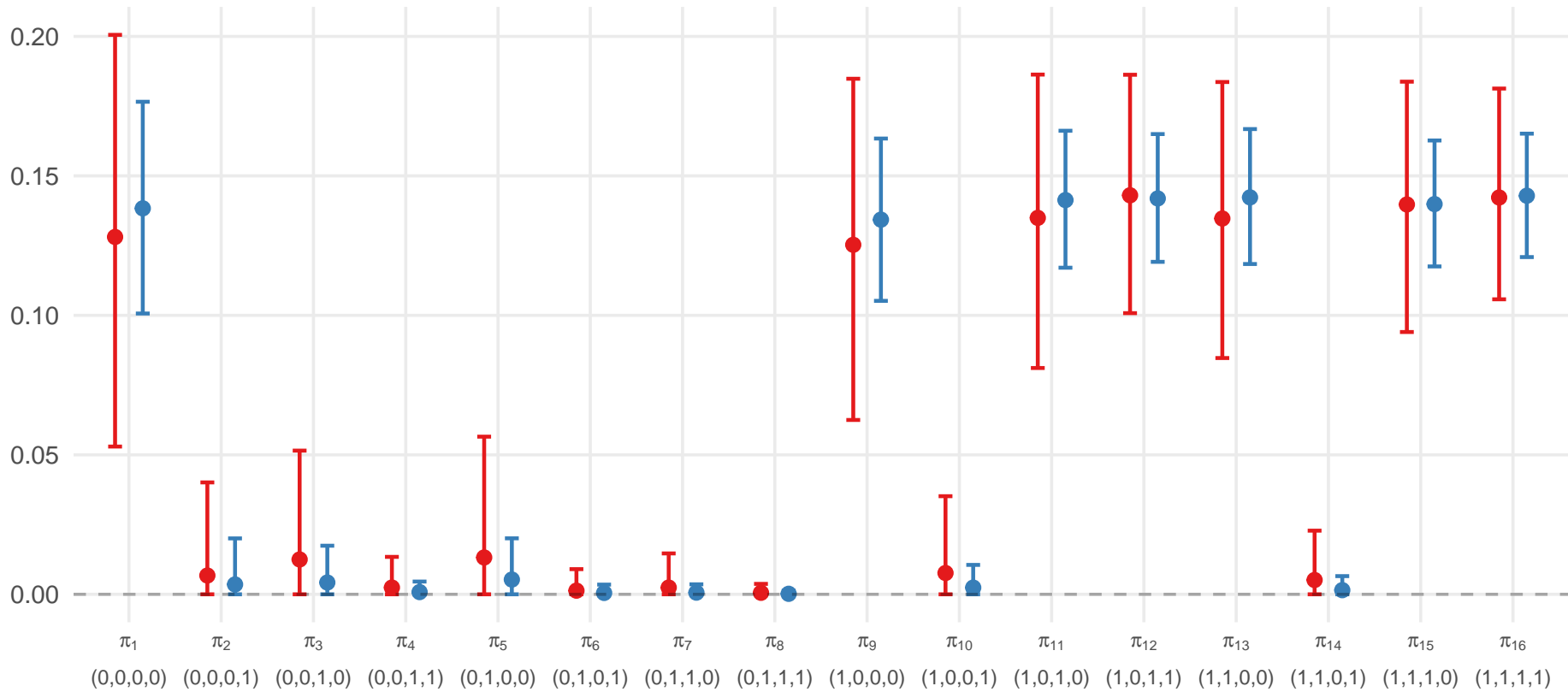

Supplement: Wang et al. supplementary material [file S0033312326100933sup001.zip › Appendix/plot/G4_balanced.pdf]

**g**

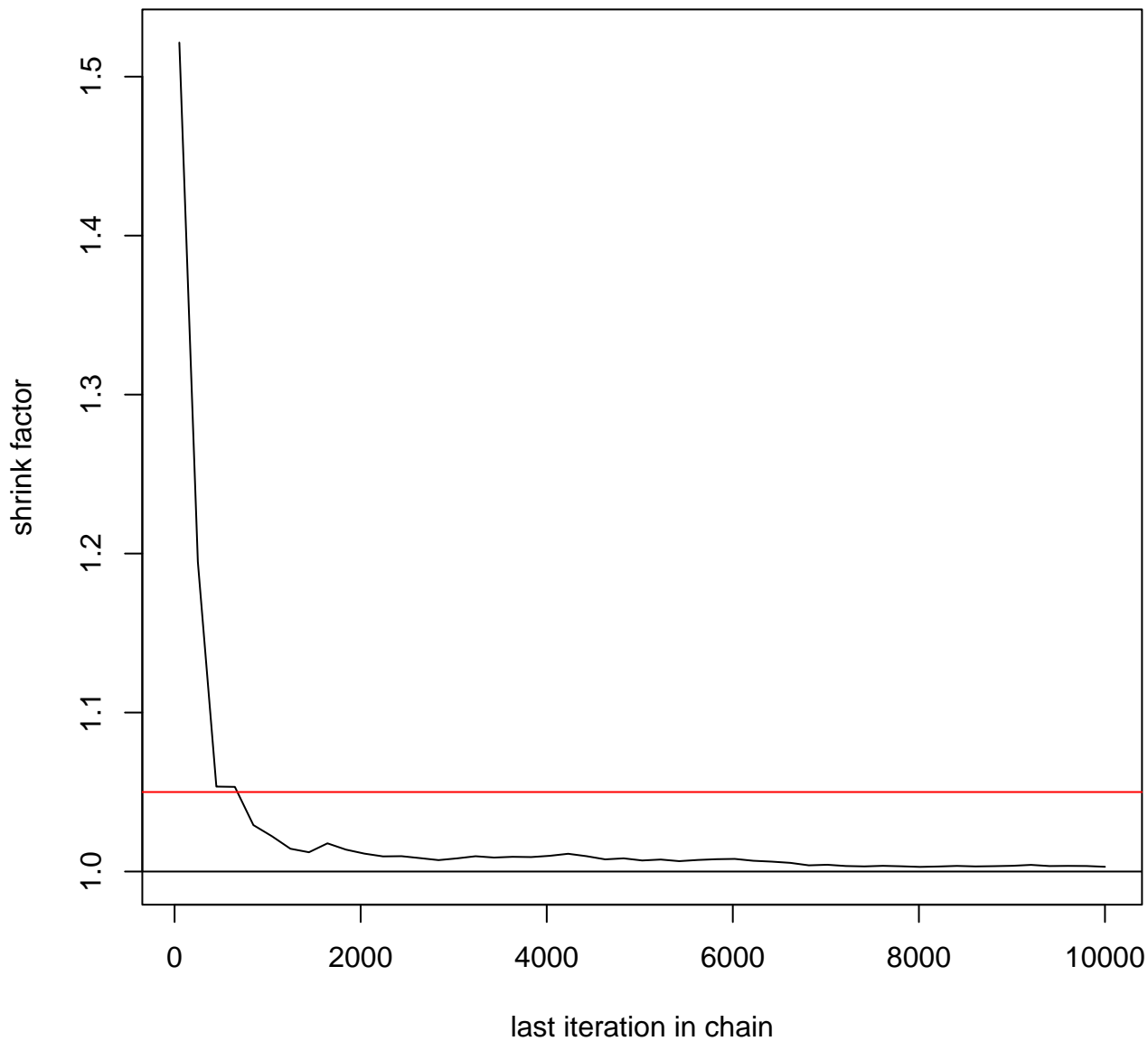

Supplement: Wang et al. supplementary material [file S0033312326100933sup001.zip › Appendix/plot/G4_N_500_J_20_sg_0.2_balanced_N1_256_g.pdf]

**S**

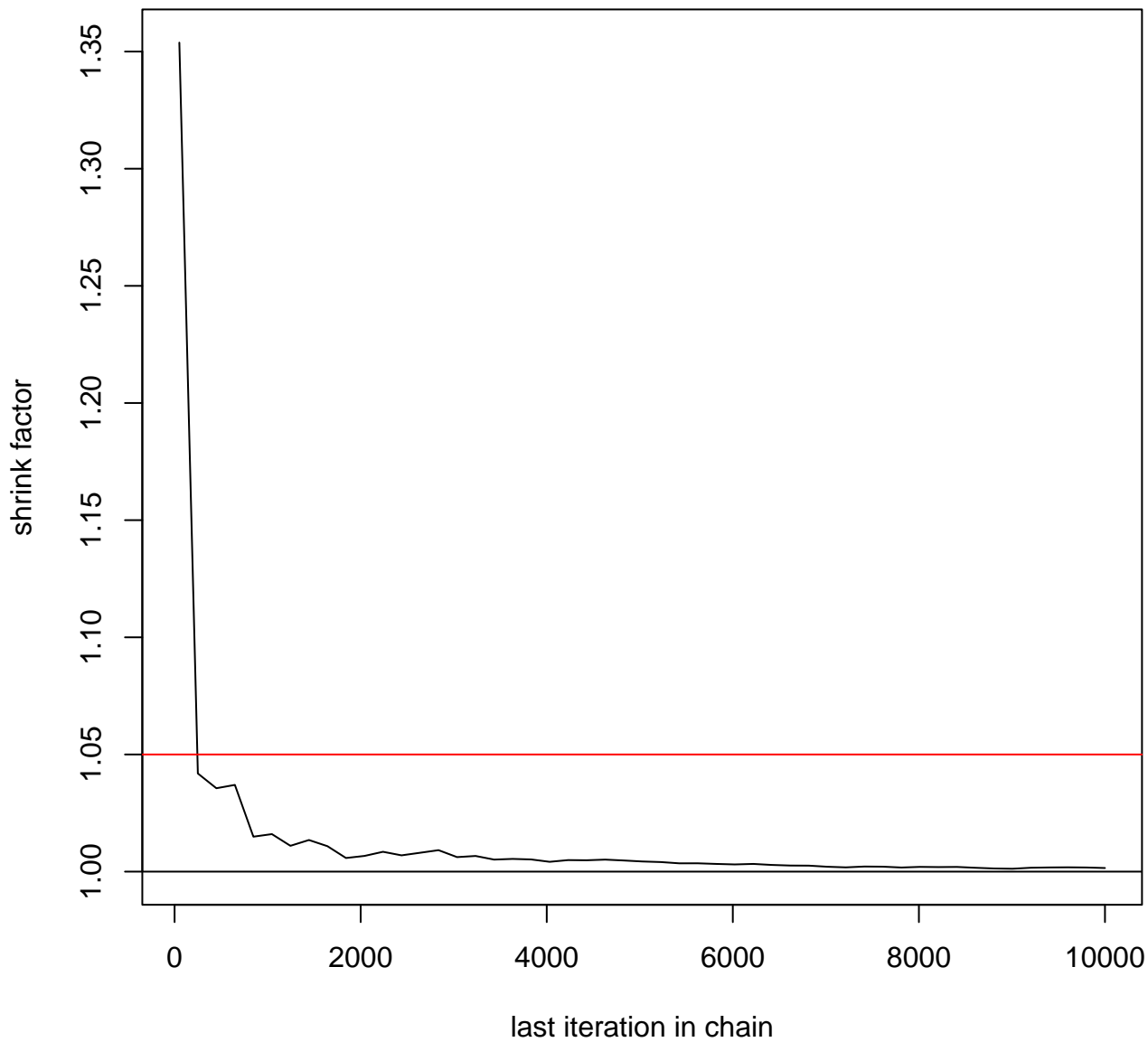

Supplement: Wang et al. supplementary material [file S0033312326100933sup001.zip › Appendix/plot/G4_N_500_J_20_sg_0.2_balanced_N1_256_s.pdf]

**g**

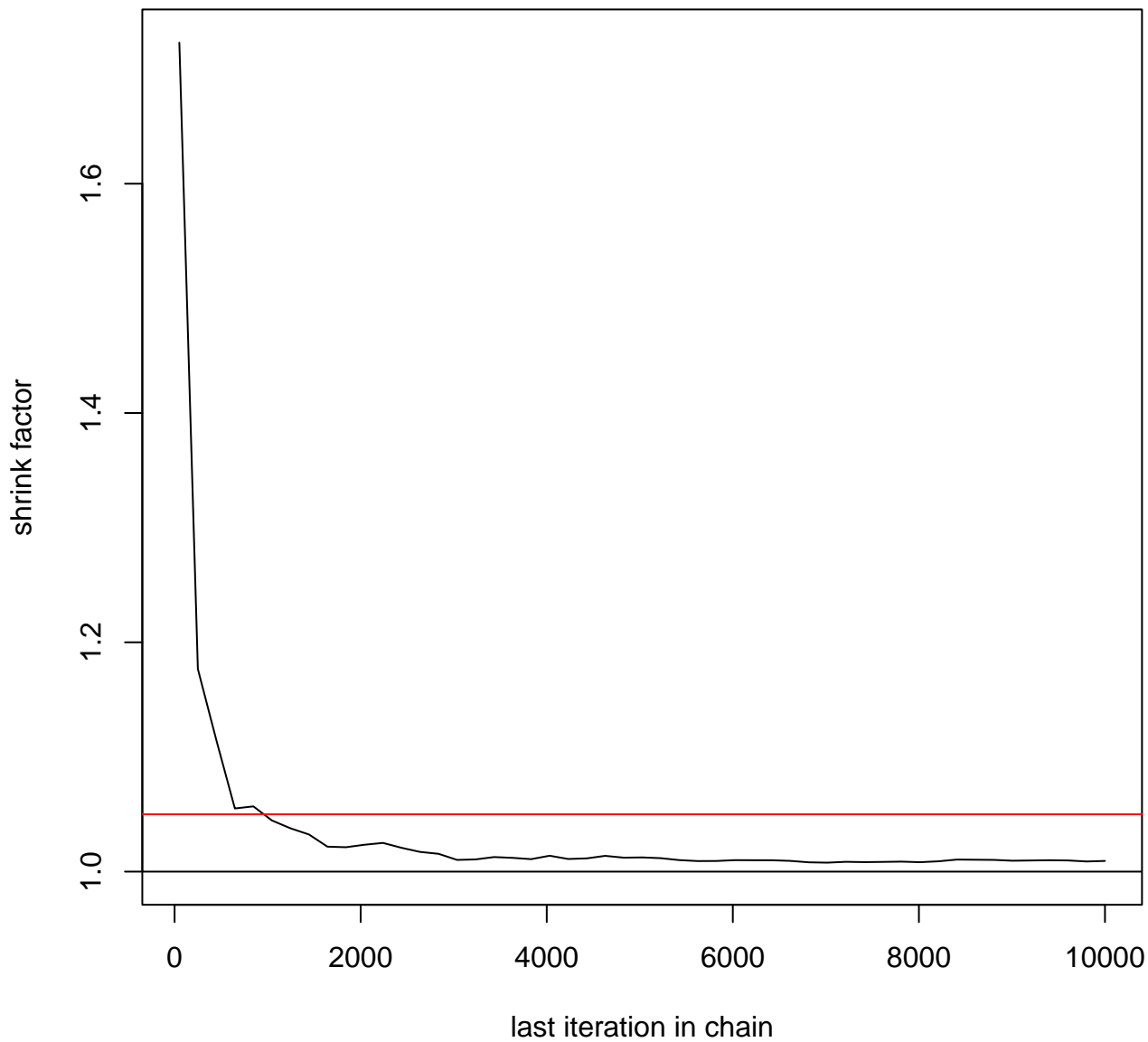

Supplement: Wang et al. supplementary material [file S0033312326100933sup001.zip › Appendix/plot/G4_N_500_J_20_sg_0.2_unbalanced_N1_256_g.pdf]

**S**

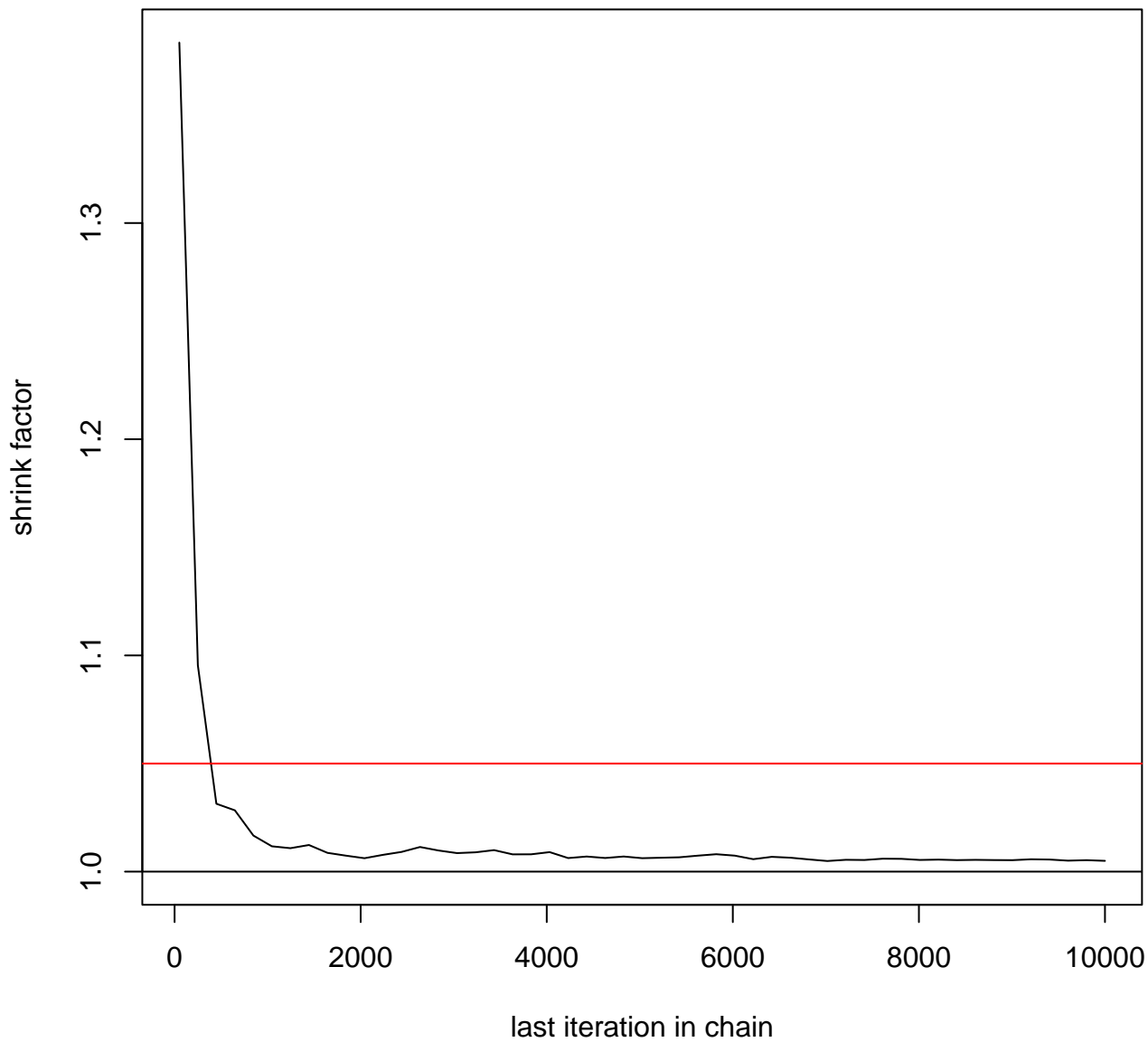

Supplement: Wang et al. supplementary material [file S0033312326100933sup001.zip › Appendix/plot/G4_N_500_J_20_sg_0.2_unbalanced_N1_256_s.pdf]

**Sample Size** ● N=500, J=20 ● N=1000, J=40

**95% AHPDI**

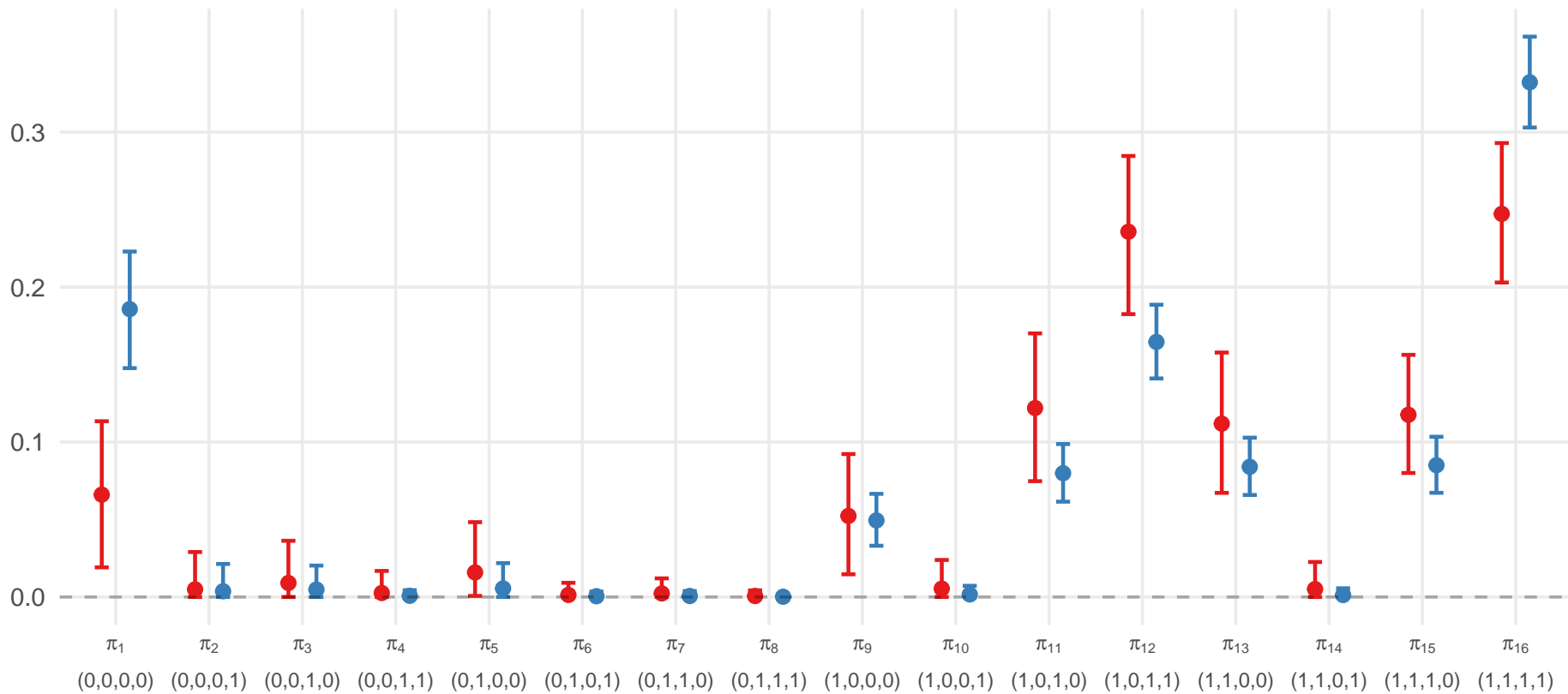

Supplement: Wang et al. supplementary material [file S0033312326100933sup001.zip › Appendix/plot/G4_unbalanced.pdf]
